# Mef2d potentiates type-2 immune responses and allergic lung inflammation Authors:

**DOI:** 10.1126/science.adl0370

**Published:** 2024-06-28

**Authors:** Aydan C.H. Szeto, Paula A. Clark, Ana C.F. Ferreira, Morgan Heycock, Emma L. Griffiths, Eric Jou, Jonathan Mannion, Shi-Lu Luan, Sophie Storrar, Martin D. Knolle, Patrycja Kozik, Helen E. Jolin, Padraic G. Fallon, Andrew N.J. McKenzie

**Affiliations:** 1 MRC Laboratory of Molecular Biology, Cambridge, CB2 0QH, United Kingdom; 4 Cambridge University Hospitals, Cambridge, CB2 0QQ, United Kingdom; 5 School of Medicine, Trinity College Dublin, Dublin, Ireland

## Abstract

Innate lymphoid cells (ILCs) and adaptive T lymphocytes promote tissue homeostasis and protective immune responses. Their production depends on the transcription factor GATA3, which is further elevated specifically in ILC2s and T helper 2 (T_H_2) cells to drive type-2 immunity during tissue repair, allergic disorders and anti-helminth immunity. The control of this crucial upregulation is poorly understood. Using CRISPR screens in ILCs we identified previously unappreciated Mef2d-mediated regulation of GATA3-dependent type-2 lymphocyte differentiation. Mef2d-deletion from ILC2s and/or T cells specifically protected against allergen lung challenge. Mef2d both repressed Regnase-1 endonuclease expression to enhance IL-33 receptor (ST2) production and IL-33 signaling, and acted downstream of calcium-mediated signaling to translocate NFAT1 to the nucleus to promote type-2 cytokine-mediated immunity.

Type-2 cytokine secretion profiles are characteristic of protective immunity to parasitic helminth infections and tissue repair following damage (1, 2). However, they also underlie inappropriate asthma and allergic responses (3, 4). Combinations of the type-2 interleukins (IL)-4, IL-5, IL-9 and IL-13 promote immune effector functions including antibody isotype switching to IgE, adaptive T helper 2 (T_H_2) cell polarization, eosinophilia, mast cell hyperplasia, goblet cell hyperplasia and tissue repair.

Group 2 innate lymphoid cells (ILC2s) and T_H_2 cells are the major type-2 cytokine-producing immune cell subsets. These related lymphocytes arise from shared common lymphoid progenitors (CLPs) in the bone marrow (5–7), but they respond and differentiate to distinct stimuli. ILC2s react rapidly to tissue damage, primarily to epithelium and stromal cell-derived initiator cytokines including IL-25, IL-33 and TSLP, which promote proliferation and cytokine expression (8). ILC2s also respond to other tissue restricted signals such as pro-inflammatory prostaglandins, leukotrienes and neuropeptides (9). T cells are, instead, activated by the T cell receptor (TCR) complex binding to specific antigen-derived peptides complexed with MHC molecules on antigen presenting cells.

Although activated differently, ILC precursors or naïve T cells both rely on the upregulation of the ‘master’ type-2 transcription factor GATA3 for differentiation into ILC2s or T_H_2 cells respectively. However, since GATA3 is also required for the development of all other ILC family members (10, 11) and all T cells (3), the dynamic changes in GATA3 regulation must be strictly controlled to maintain tonic GATA3 in naïve cells but promote GATA3 upregulation to support type-2 immunity. Thus, although GATA3 is considered the master regulator of T_H_2 cells and ILC2s, we still have an incomplete understanding of how high levels of type-2-permissive GATA3 expression are induced and sustained to support allergen and antigen-mediated type-2 immune responses. In addition, since GATA3 blocks the T_H_1/17 transcriptional program, it must also be maintained at lower levels during immune reactions to bacterial, viral and fungal infections. To address the fundamental question of how GATA3 is regulated in type-2 biology we generated multi-reporter mouse strains and optimized ILC-progenitor differentiation cultures to overcome the rarity of these cells and permit CRISPR-Cas9 screens for critical determinants of GATA3 and ILC2 development and function. Furthermore, to discriminate the *in vivo* roles and influence of candidate molecules in ILC2s and/or T_H_2 cells we developed and extensively verified a modular mouse model in which a Boolean logic approach, utilizing three site-specific DNA recombinases (SSRs) with intersectional expression patterns, which restricts cellular and molecular manipulation to ILC2s without affecting phenotypically similar T_H_2 cells or other ILC subsets.

## Results

### A CRISPR-Cas9 screen identifies regulators of GATA3 during ILC2 development

**Fig. 1:**
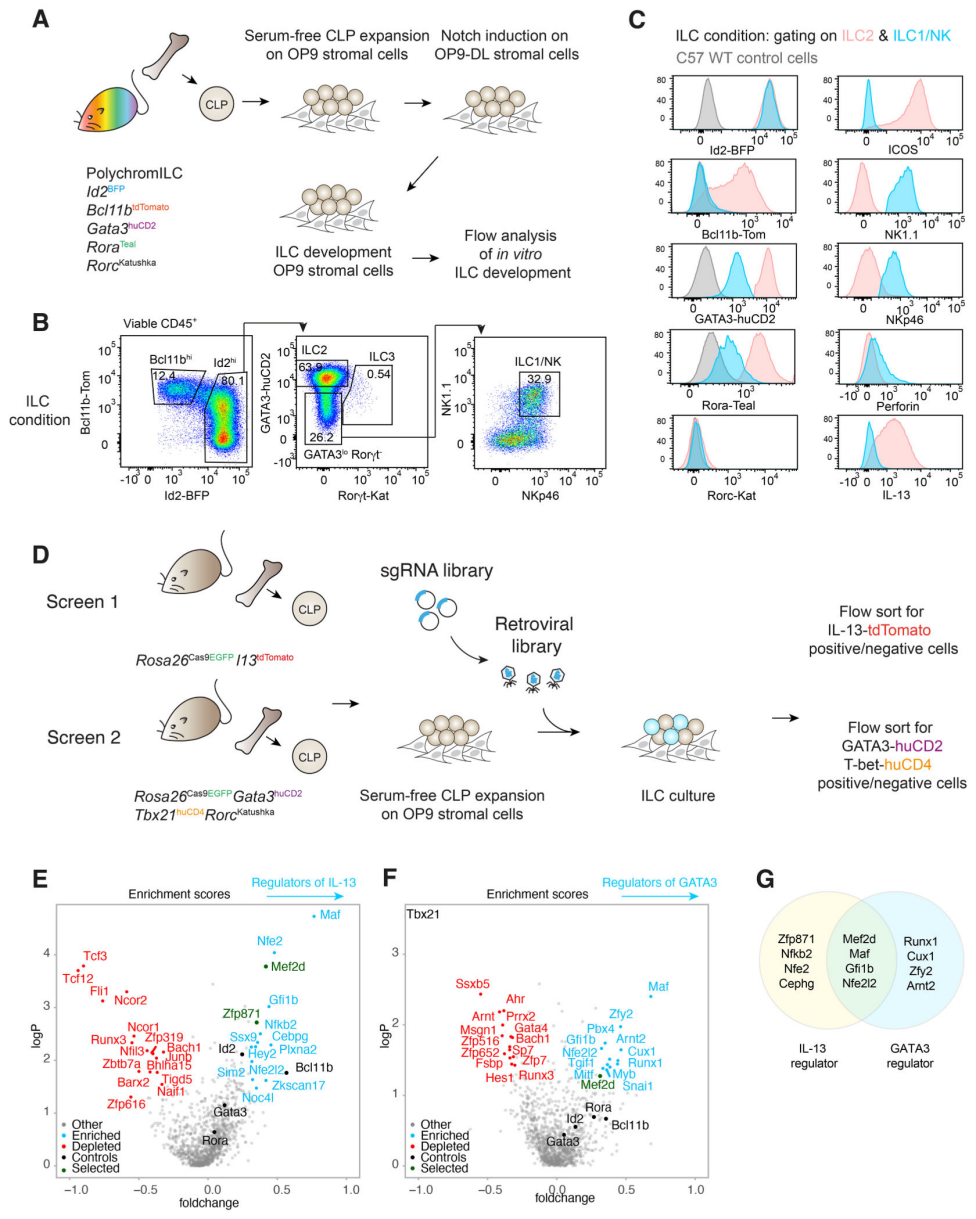
CRISPR-Cas9 screen for regulators of ILC development and function (A) Schematic of the optimised ILC culture protocol for CRISPR screening, validated using the 5x polychromILC mice. (B) Gating strategy and flow cytometric analysis of progeny cells following the ILC culture of sorted CLPs purified from 5x polychromILC mice. Data are representative of 2 independent experiments with n=3 biologically independent samples in each experiment. (C) Flow cytometric analysis of surface protein, transcription factor reporter protein and cytokine expression by ILC2s and ILC1/NK cells following the ILC culture as in (A) and (B). Flow plots are presented as histograms and the y-axis represents distribution normalized to mode. The lower level of GATA3 expression in ILC1/NK cells compared to ILC2s represented an opportunity to identify transcriptional regulators that control differentiative GATA3 expression during ILC development by comparing sgRNA distribution between GATA3 high versus low cells. Data are representative of 2 independent experiments with n=3 biologically independent samples in each experiment. (D) Schematic of the CRISPR-Cas9 screening protocol for the identification of *Gata3* and *Il13* regulators using the ILC culture. (E) Volcano plot showing known (black), positive (blue) and negative (red) regulators of *Il13* expression, represented as -log(p-value) versus fold change. Mef2d and Zfp871 are highlighted in green. Data are pooled from 2 independent screens. (F) Volcano plot showing known (black), positive (blue) and negative (red) regulators of *Gata3* expression, represented as -log(p-value) versus fold change. Mef2d is highlighted in green. Data are pooled from 2 independent screens. (G) Venn diagram summary of specific and shared regulators of *Il13* and *Gata3* expression identified from the CRISPR screens.

To identify regulators of GATA3 and IL-13 expression during ILC development and differentiation we performed CRISPR screens using transcription factor and cytokine reporter mice to provide CLPs which were *in vitro* expanded and transduced with a sgRNA library targeting 1131 transcription factors (TFs) (Fig. 1A-D, fig. S1A-D). We identified known positive regulators of ILC2 development including *Bcl11b, Id2, Gata3, Rora* and *Maf* (Fig. 1E and Data File S1) (6, 12–15). Candidate positive regulators of IL-13 included *Mef2d, Zfp871, Nfkb2, Nfe2* and *Gfi1b*, while negative regulators included *Runx3, Tcf12, Nfil3, Fli1* and *Zbtb7a* (Fig. 1E) (16, 17). Potential regulators of GATA3 included *Pbx4, Arnt2, Gfi1b, Runx1, Nfe2l2, Tgif1, Snai1, Zfy2 Mitf, Mef2d, Myb, Maf* and *Cux1* (Fig. 1F and fig. S1F and Data file S1). Selected candidates were validated (fig. S1E). Notably, *Mef2d* was identified in both screens (Fig. 1G), which suggested that myocyte-specific enhancer factor 2d (Mef2d) could play a previously unappreciated, role in promoting GATA3 and IL-13 expression in ILC2s.

### Mef2d promotes type-2 immune responses *in vivo*

Mef2d is a member of the Mef2a-d family of transcription factors which bind to A/T-rich DNA sequences and have dual functions in gene activation and repression during cellular development and differentiation in response to calcium-dependent signaling (18). Roles have been reported for Mef2d in regulating T cell apoptosis, IL-2 production by T cells and early B cell development (19–21).

To assess the importance of Mef2d in lymphocyte biology *in vivo* we generated mice in which ILCs, T cells and B cells are deficient in Mef2d by intercrossing *Il7r*^Cre^ and *Mef2d*^flox^ mice (*Il7r*^Cre^*Mef2d*^f/f^, herein *Mef2d*^IL7RKO^ mice) (fig. S2A and B). At homeostasis, *Mef2d*^IL7RKO^ mice harbored normal numbers of lung ILC2s that expressed lower levels of GATA3 compared to controls (fig. S2C), consistent with the identification of Mef2d as a regulator of GATA3 expression. Comprehensive phenotyping revealed equivalent lymphoid progenitor and peripheral cell populations at homeostasis in *Il7r*^Cre^ and *Mef2d*^IL7RKO^ mice (fig. S2D, fig. S3A and B).

**Fig. 2:**
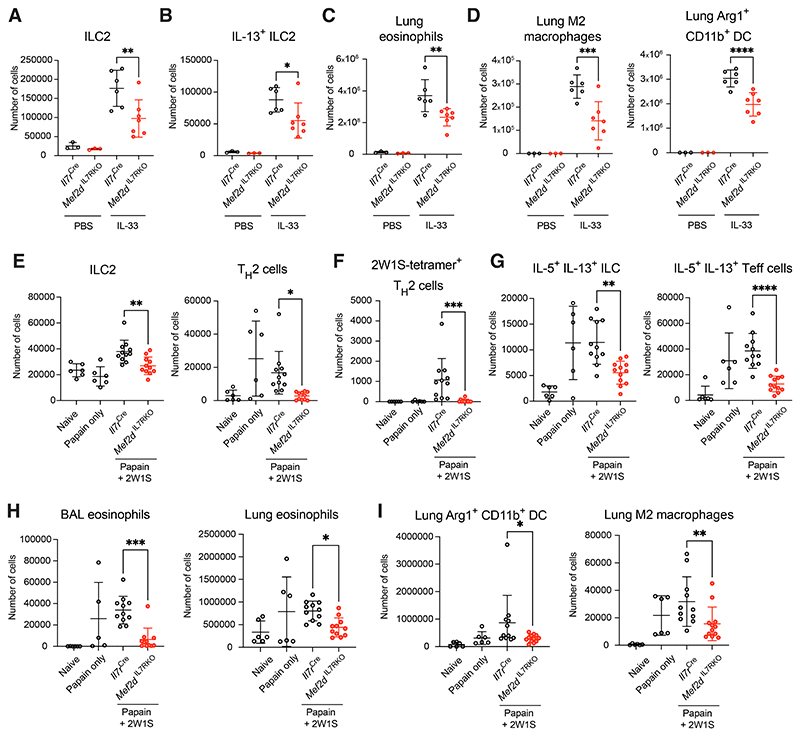
Mef2d expression in lymphocytes is required for optimal innate and adaptive type-2 immune responses (A) − (D) Quantification of lung cells from PBS or IL-33 treated *Il7r*^Cre^ or *Mef2d*^IL7RKO^ mice: (A) ILC2s, (B) IL-13-expressing ILC2s, (C) eosinophils, (D) M2 macrophages and Arg1^+^CD11b^+^ DCs. Data are pooled from 2 independent experiments and represent mean ± SD; n=3 mice (PBS groups) and n=6-7 mice (IL-33 groups); individual data point denotes biological replicates. (E) − (I) Quantification of lung cell from naïve, papain or papain+2W1S treated *Il7r*^Cre^- or *Mef2d*^IL7RKO^ mice: (E) number of ILC2s and T_H_2 cells, (F) number of 2W1S-specific T_H_2 cells, (G) number of IL-5^+^IL-13^+^ ILC and T effector cells, (H) number of BAL and lung eosinophils, (I) number of lung Arg1^+^CD11b^+^ DCs and M2 macrophage. Data are pooled from 2 independent experiments and represent mean ± SD; n=6 mice in naïve and papain only groups, n=11-12 mice in papain+2W1S-treated groups; individual data point denotes biological replicates. Significance in (A) − (I) was determined using one-way ANOVA with Dunett’s post-hoc test; *P<0.05; **P<0.01; ***P<0.001; ****P<0.0001.

*Mef2d*^IL7RKO^ mice and *Il7r*^Cre^ controls were treated intranasally with the cytokine IL-33 which increases ILC2 proliferation and type-2 cytokine expression (fig. S4A). IL-33-treated *Mef2d*^IL7RKO^ mice had fewer lung ILC2s (Fig. 2A, fig. S4B) and IL-13^+^ ILC2s (Fig. 2B, fig. S4C) than controls, while CD4^+^ and CD8^+^ T cells, and B cell numbers were not altered (fig. S5A). We also noted fewer IL-5-dependent eosinophils (Fig. 2C) and impaired IL-13-dependent differentiation of M2 macrophages and arginase-positive dendritic cells (DCs) (Fig. 2D). By contrast, expression of the type-1 cytokine IFN-γ by innate and adaptive lymphocytes was not impaired by Mef2d-deficiency (fig. S5B). Similarly, when *Mef2d*^IL7RKO^ mice were challenged intranasally with allergen extracts from the mold *Alternaria alternata* (fig. S5C), a clinically relevant allergen that elicits ILC2 activation and allergic immune responses, there were fewer ILC2s in their lungs as compared to controls (fig. S5D). This was associated with a reduction in bronchoalveolar lavage (BAL) eosinophils and lung M2 macrophages, and reduced expression of Arg1 by M2 macrophages and type-2 polarized CD11b^+^ DCs (fig. S5E), whereas type-1 and type-17 immunity were not affected (fig. S5F). Thus, Mef2d plays critical roles in regulating rapid innate type-2 immune reactions.

ILC2s have been reported to regulate adaptive type-2 immunity by promoting T_H_2 cell differentiation (22, 23). We sensitized and re-challenged *Mef2d*^IL7RKO^ and *Il7r*^Cre^ control mice with intranasal papain that, in combination with 2W1S peptide, initiates robust pulmonary type-2 inflammation and the development of 2W1S-specific T_H_2 cells (fig. S5G). After re-challenge, a timepoint at which adaptive immunity peaks, *Mef2d*^IL7RKO^ mice had fewer lung ILC2s and T_H_2 cells (Fig. 2E). Strikingly, within the T_H_2 cell compartment, the development of 2W1S-specific T_H_2 cells was almost completely abrogated in *Mef2d*^IL7RKO^ mice (Fig. 2F). We also observed a reduction in IL-5 and IL-13-expressing ILC2s and T effector cells (Fig. 2G), which was associated with decreased BAL and lung eosinophilia (Fig. 2H), and reductions in type 2-polarized CD11b^+^ DCs and M2 macrophages (Fig. 2I). IFN-γ production was unchanged in this model (fig. S5H). These results confirmed a role for lymphoid-derived Mef2d in regulating innate and adaptive type-2 immunity. In contrast, Mef2d appeared dispensable for type-1/17 immunity as the responses to *Citrobacter rodentium* seemed intact (fig. S5I and S5J).

### Intersectional recombinases enable ILC2-specific gene targeting

We next aimed to better examine the influence of Mef2d within ILC2s and T cells, and avoid collateral effects on other lymphocytes. However, there remain substantial challenges in assigning specific functions to precise ILC subsets. Indeed, as our understanding of the subtleties of ILC identity and function has grown, the tools available to dissect their roles and the key genes involved have struggled to keep pace. This is because of the challenge in identifying Cre-driver genes to [1] target each individual ILC subset without concurrently leading to collateral effects on other cell types including phenotypically similar T cell subsets (22–24), or [2] to facilitate ILC2-specific gene deletion (25). Indeed the recently reported Cre expression from an *Nmur1* gene promoter-containing BAC transgenic (26, 27) may risk collateral interference in non-haematopoietic cells (28), T cells (29–32) (fig. S6A − F) and eosinophils (33).

**Fig. 3:**
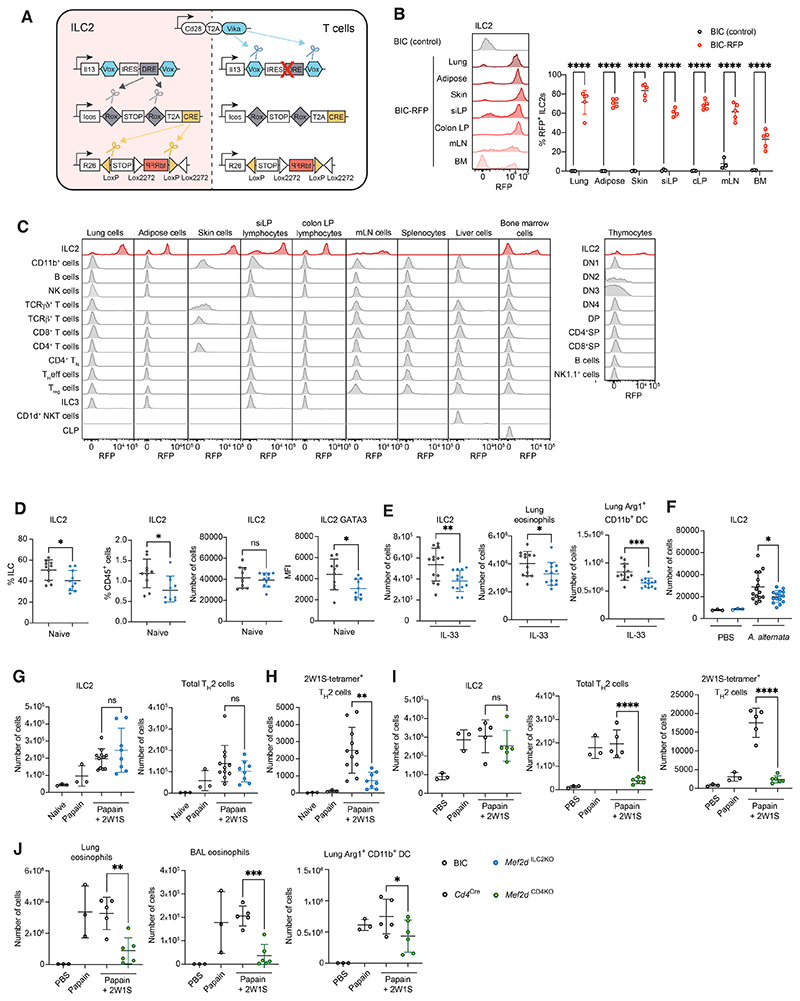
Development of Boolean mouse strains for ILC2-specific gene targeting define the roles of Mef2d in innate and adaptive type-2 immunity (A) Schematic of Boolean recombinase cascade with RFP readout. (B) Flow cytometric analysis of RFP expression by ILC2s in the lung (GATA3^+^ST2^+^), adipose (GATA3^+^ST2^+^), skin (integrinβ3^+^TCRγδ−), small intestinal lamina propria (GATA3^+^RORγt^−^), colonic lamina propria (GATA3^+^RORγt^−^), mesenteric lymph node (GATA3^+^RORγt^−^) and bone marrow (ST2^+^CD25^+^). (C) Flow cytometric analysis of RFP expression by immune cell populations from various tissues. Where a cell population is not present or was not investigated in a particular tissue a histogram is replaced by a flat line in the relevant panel. (B) and (C) Representative gating strategies for cell populations investigated shown in fig. S3 and fig. S10 (Skin) and fig. S13 (liver NKT cells). Data are representative of 2 independent experiments with n=5 biologically independent samples in each experiment; mean ± SD. (D) Quantification of lung ILC2s as a percentage of ILC, percentage of CD45^+^ cells, number, and ILC2 GATA3 MFI (mean fluorescence intensity) from BIC or *Mef2d*^ILC2KO^ mice at homeostasis. Data are representative of 2 independent experiments and represent mean ± SD; n=3 mice in experiment 1, n=10 in experiment 2 (depicted here). (E) Quantification of lung ILC2, eosinophils, and Arg1^+^CD11b^+^ DCs from IL-33 treated BIC or *Mef2d*^ILC2KO^ mice. Data are pooled from 2 independent experiments and represent mean ± SD; n=13 mice in each group. (F) Quantification of lung ILC2s from PBS or *A. Alternata*-treated BIC or *Mef2d*^ILC2KO^ mice. Data are pooled from 3 independent experiments with n=3 mice in PBS groups, n=15 mice in *A. Alternata* groups; mean ± SD. (G) − (H) Quantification of lung cells from naive, papain or papain+2W1S treated BIC or *Mef2d*^ILC2KO^ mice: (G) number of ILC2s and total T_H_2 cells, (H) number of 2W1S-specific T_H_2 cells. Data are pooled from 2 independent experiments and represent mean ± SD; n=3 in naïve and papain only groups, n=11 in papain+2W1S-treated control group, n=8 in papain+2W1S- treated *Mef2d*^ILC2KO^ group. (I) − (J) Quantification of lung cells from PBS, papain or papain+2W1S treated *Cd4*^Cre^ or *Mef2d*^CD4KO^ mice: (I) number of ILC2, total T_H_2 cells and 2W1S-specific T_H_2 cells, (J) number of lung eosinophils, BAL eosinophils and lung Arg1^+^CD11b^+^ DCs. Data are representative of 2 independent experiments and represent mean ± SD; n=3 in naïve and papain only groups, n=5-6 in papain+2W1S-treated groups. Significance was determined using unpaired two-sided t-test [(B) − (E)] or one-way ANOVA with Dunett’s post-hoc test [(F) − (J)]; ns, not significant; *P<0.05; **P<0.01; ***P<0.001; ****P<0.0001; individual data point denotes biological replicates.

Therefore, we implemented a Boolean approach in which we introduced three different DNA recombinases into the endogenous loci encoding ICOS, IL-13 and CD28 to target mature ILC2s, such that the expression of each recombinase is contingent on the promoter expression of each targeted allele (Fig. 3A and fig S7). Consequently, the Cre gene will only be expressed in cells that express *Icos* (*Icos*^Rox-STOP-RoxT2ACre^, hereafter referred to as *Icos*-Cre, fig. S8A and B) ‘AND’ have expressed *Il13* (*Il13*^Vox-IRES-Dre-Vox^, hereafter referred to as *Il13*-Dre, Fig. S8C and D), ‘AND NOT’ previously expressed *Cd28* (*Cd28*^T2AVika^, hereafter referred to as *Cd28*-Vika, fig. S8E and F) which is not expressed by ILC2s (Immgen and fig. S8G).

The individual mouse strains were inter-crossed to produce heterozygous Boolean-ILC2-Cre (BIC) mice (fig. S7 and S8H). Heterozygous BIC mice had normal numbers of homeostatic lymphoid progenitor (bone marrow and thymus) and peripheral populations (spleen, lymph node and lung) (fig. S9A); expressed normal CD28 and IL-13 levels and modestly decreased ICOS levels (fig. S9B); and mounted an equivalent type-2 effector program *in vitro* (fig. S9C & D) and *in vivo* (fig. S9E) compared to C57 controls. The *in vivo* efficacy of the system was validated by intercrossing BIC mice with *Rosa26*-tdRFP Cre-reporter mice (BIC-RFP) and assessing RFP expression across tissues and cell types. These analyses confirmed RFP-positivity as highly restricted to ILC2s (Fig. 3B and C, fig. S11A − I), and this was supported by unbiased tSNE analysis (S12A − C).

The *Cd28-*Vika allele protected CD4^+^ T cells cultured under strongly T_H_2 cell polarizing conditions from expressing RFP (fig. S13A) and *in vivo* (fig. S13B). Similar results were also observed for naïve liver RFP^+^ NKT cells (Fig. S13C and D). The rare numbers of RFP^+^ NKT cells in the liver and thymus (~1% of total thymic NKT cells) had a NKT1 phenotype, marked by T-bet expression (Fig. S13E), suggesting that they are not functional NKT2 cells and that Cre fate mapping likely represents a historical event during NKT development (34). Importantly, NKT and T_H_2 cells remain protected from Cre activity even in the context of strong type-2 stimuli, including intranasal papain challenge (fig. S14A − C) and *N. brasiliensis* infection (fig. S14D). By contrast, expression of RFP was similar in ILC2s irrespective of the presence of the *Cd28-*Vika allele (fig. S14A − C). Mice lacking the *Il13-*Dre allele (BIC-*Il13*^WT^-RFP) were negative for RFP^+^ cells (fig. S14A − B).

We intercrossed the BIC mice with iDTR mice (BIC-DTR mice, fig. S8H), in which Cre-mediated excision of a STOP cassette permits Diphtheria toxin receptor (DTR) expression, to assess the efficiency of the BIC mice to delete ILC2s temporally and specifically. Following DTx administration we observed remarkable ablation of ILC2s within the lung and small intestinal lamina propria of the BIC-DTR mice, as compared to the DTx-treated BIC controls, even in naïve mice (fig. S15A - C). Ablation was also efficient in the adipose tissue and skin (fig. S15D and E) and in the bone marrow (fig. S15F). We further investigated two models of type-2 skin inflammation (*Alternaria alternata* extract and calcipotriol) (fig. S16A-K) and two models of type-2 lung inflammation (IL-33 (fig. S17A-F) and papain (fig. S18A-E)). In all instances investigated, DTx-mediated ILC2 ablation was effective, and resulted in a parallel fall in the proportion of eosinophils, confirming the role previously attributed to ILC2s in regulating eosinophils through their IL-5 production (24, 25) and regulation of eotaxins (35). In addition, the papain model allowed us to evaluate the specific contribution of ILC2s to primary and secondary immune responses. Our results strongly support the critical role of ILC2s during primary immune sensitization in supporting optimal T_H_2 cell responses and the repression of type-1 inflammation (fig. S18B and C), whereas ILC2 activity during recall challenge after the primary sensitization was less important (fig. S18D).

### Mef2d regulates innate and adaptive type-2 immunity

Having validated their specificity and efficacy we intercrossed the BIC mice with the *Mef2d*^f/f^ mice to produce *Mef2d*^ILC2KO^ mice (fig. S19A). At homeostasis, there was a modest reduction of lung ILC2s as a percentage of ILCs and total CD45^+^ cells, although the number of lung ILC2s was unchanged in *Mef2d*^ILC2KO^ mice (Fig. 3D). Consistent with earlier results, *Mef2d*^ILC2KO^ mice harbored ILC2s with reduced GATA3 MFI (Fig. 3D). In the intranasal-IL-33 challenge model, *Mef2d*^ILC2KO^ mice developed lower levels of type-2 inflammation as evidenced by reduced numbers of lung ILC2s, eosinophils and Arg1-expressing CD11b^+^ DCs (Fig. 3E). In an *A. alternata* model, we also observed a reduction in lung ILC2 numbers in *Mef2d*^ILC2KO^ mice elicited by intranasal challenge (Fig. 3F), without major changes in downstream myeloid responses (fig. S19B) or type-1/17 cytokine expression (fig. S19C). When challenged with papain and 2W1S peptide to provoke adaptive immunity, *Mef2d*^ILC2KO^ mice developed similar numbers of ILC2s, total T_H_2 cells (Fig. 3G), and innate and adaptive type-1/17 lymphocytes (fig. S19D). However, we observed a striking reduction in 2W1S-specific T_H_2 cells in *Mef2d*^ILC2KO^ mice, mirroring the results we observed upon ILC2 depletion in BIC-DTR mice, highlighting the role of Mef2d in enabling ILC2s to support the generation of antigen-specific T_H_2 immunity (Fig. 3H).

The relative difference between the magnitude of the response in the *Mef2d*^ILC2KO^ mice and the *Mef2d*^IL7RKO^ mice also suggested a role for Mef2d in other lymphocytes in the papain model. To address the role of Mef2d in T cells we crossed *Mef2d*^f/f^ mice with *Cd4*^Cre^ mice to develop *Mef2d*^CD4KO^ mice (fig. S19E). Papain and 2W1S elicited a normal ILC2 response in *Mef2d*^CD4KO^ mice as would be anticipated, whereas total and 2W1S-specific T_H_2 cells were drastically reduced (Fig. 3I), leading to decreased eosinophilia in the lung and BAL fluid, and lung Arg1^+^ CD11b^+^ DC numbers (Fig. 3J). IFN-γ and IL-17A expression were not affected by CD4-specific Mef2d-deficiency (fig. S19F). These results highlight the prominence of T_H_2 cells over ILC2s in the papain + 2W1S antigen recall model in driving downstream type-2 effector myeloid responses, which require Mef2d expression in T_H_2 cells.

Collectively, our results using *Mef2d*^ILC2KO^ mice confirmed the contribution of Mef2d in promoting acute models of type-2 inflammation. Furthermore, they indicated that Mef2d-regulated ILC2s and T_H_2 cells work together to drive maximal responses during the generation of adaptive type-2 immunity. Our data showed that Mef2d is specifically required for driving optimum type-2 immunity, but dispensable for type-1/17 inflammation across multiple models.

### Mef2d regulates ILC2 responses to IL-33

**Fig. 4:**
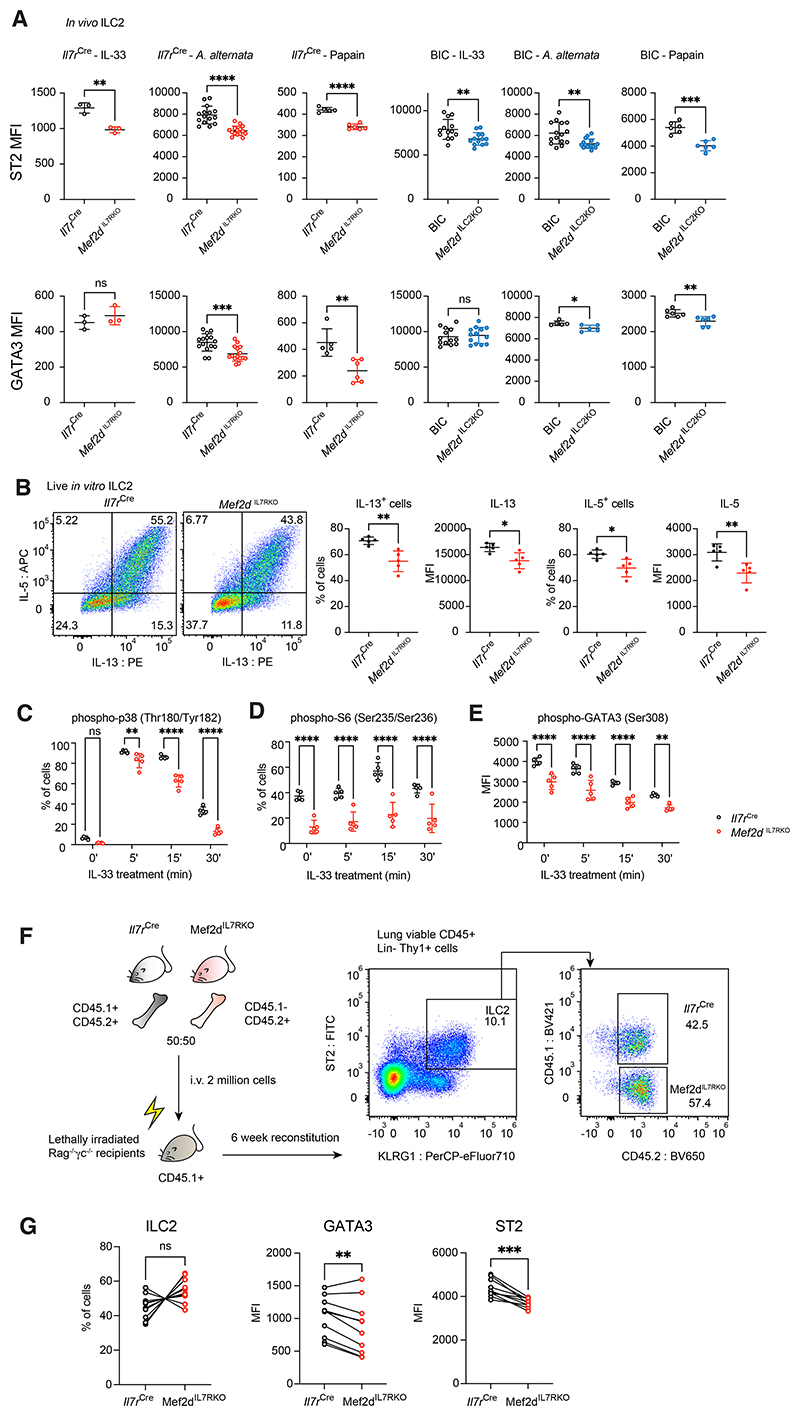
Mef2d sustains high ST2 expression and optimal IL-33-mediated ILC2 responses (A) Quantification of ILC2 ST2 and GATA3 MFI in various *in vivo* models from control or conditional Mef2d-deficient mice. Data are representative of 2-3 independent experiments with n=3-15 mice from different experiments as described in the legends of figures 2 & 3; mean ± SD. (B) Flow cytometric quantification of IL-13 and IL-5 expression following 3 days of culturing purified ILC2s in the presence of IL-33. Data are representative of 2 independent experiments with n=5 biologically independent samples in each experiment; mean ± SD. Gating strategy for scatter, singlets and live/dead cell exclusion shown in fig. S20A. (C) − (E) Flow cytometric quantification of (C) phospho-p38, (D) phospho-S6 and (E) phospho-GATA3 following IL-33 treatment for the indicated time. Data are representative of 2 independent experiments with n=5 biologically independent samples in each experiment; mean ± SD. (F) Schematic of the mixed bone marrow chimera experiment and representative gating strategy for the identification of *Il7r*^Cre^- or *Mef2d*^IL7RKO^-derived ILC2s in the recipients. (G) Quantification of the proportion of *Il7r*^Cre^- or *Mef2d*^IL7RKO^-derived ILC2s (left) and their GATA3 and ST2 MFI (right). Data are representative of 2 independent experiments with n=6-9 mice in each experiment; paired samples (*Il7r*^Cre^- or *Mef2d*^IL7RKO^-derived ILC2s from the same recipients) are connected by a line. Significance in (A) − (G) was determined using unpaired two-sided t-test; ns, not significant; *P<0.05; **P<0.01; ***P<0.001; ****P<0.0001; individual data point denotes biological replicates.

Across the majority of the *in vivo* experimental models tested Mef2d-deficiency associated with reduced expression of IL-33 receptor (ST2) and GATA3 by ILC2s (Fig. 4A). ST2 is GATA3-regulated and binds IL-33 which promotes ILC2 (36, 37) and T_H_2 cell (38–40) proliferation and type-2 cytokine expression, and can synergize with co-stimulators such as leukotrienes to enhance ILC2 responses (41). *In vitro*, the deficit in ST2 expression on ILC2s from *Mef2d*^IL7RKO^ mice resulted in impaired IL-13 and IL-5 production in response to IL-33 stimulation (Fig. 4B, fig. S20A), although proliferation was normal (fig. S20B). This defect in Mef2d-deficient ILC2s was associated with a marked reduction in the activation of downstream signaling molecules including phospho-p38 (Fig. 4C), phospho-S6 (Fig. 4D) and phospho-GATA3 (Fig. 4E), which are known downstream mediators in the IL-33/ST2-elicited signaling cascade (42). Indeed, perturbation of GATA3 expression in ILC2s from *Gata3*^fl/fl^-CreER^T2^ mice indicates that a feedback loop exists in which GATA3 is required for ST2 expression and IL-33 signaling is required for GATA3 activation via phosphorylation (10) and this can promote type-2 cytokine gene regulation. To investigate if Mef2d-deficient ILC2s harbored a cell-intrinsic defect with respect to GATA3 and ST2 expression, mixed bone marrow experiments were performed in which congenically marked control bone marrow cells on the *Il7r*^Cre^ background (CD45.1/2) provide a source of Mef2d-sufficient T cells and ILC2s which develop alongside Mef2d-deficient lymphocytes (including ILC2s) (CD45.2) in the same host recipient (CD45.1) (Fig. 4F). Pairwise comparison of control versus Mef2d-deficient ILC2s in the same mice revealed no bias in the proportions of *Il7r*^Cre^ or Mef2d^IL7RKO^ ILC2s (Fig. 4G), but there was a cell-intrinsic defect in GATA3 and ST2 expression (Fig. 4G). These results confirmed that the absence of Mef2d leads to reduced ST2 levels which impair the IL-33-elicited downstream signaling pathway, including type-2 cytokine production (fig. S20C).

### Mef2d potentiates divergent ILC2 tissue phenotypes

Lung ILC2s are strongly regulated by the GATA3/ST2 axis. However, intestinal ILC2s exhibit a divergent gene expression program resulting in the preferential expression of IL-25R over ST2, and a prominent response to Tuft cell-derived IL-25 (43). Furthermore, other intestinal ILC subsets (e.g. ILC3) express intermediate GATA3 levels. To investigate whether Mef2d also regulates intestinal ILC gene expression programs we phenotyped intestinal ILCs and found that Mef2d-deficiency did not affect the expression of the ILC subset-specific master transcription factors (GATA3, RORγt and Eomes), or IL-25R on intestinal ILC2s (fig. S20D). Conversely, ST2 expression by ILC2s from multiple tissues including lung, adipose and bone marrow was reproducibly reduced by Mef2d-deficiency, either driven by *Il7r*^Cre^ or BIC (fig. S20E). Collectively, our data point to a role for Mef2d in regulating the GATA3/ST2 axis specifically in ST2-expressing ILC2s, whereas intestinal IL25R-expressing ILC2s were less dependent upon Mef2d.

### Mef2d regulates GATA3 and ST2 expression by repressing the negative regulator Regnase-1

**Fig. 5:**
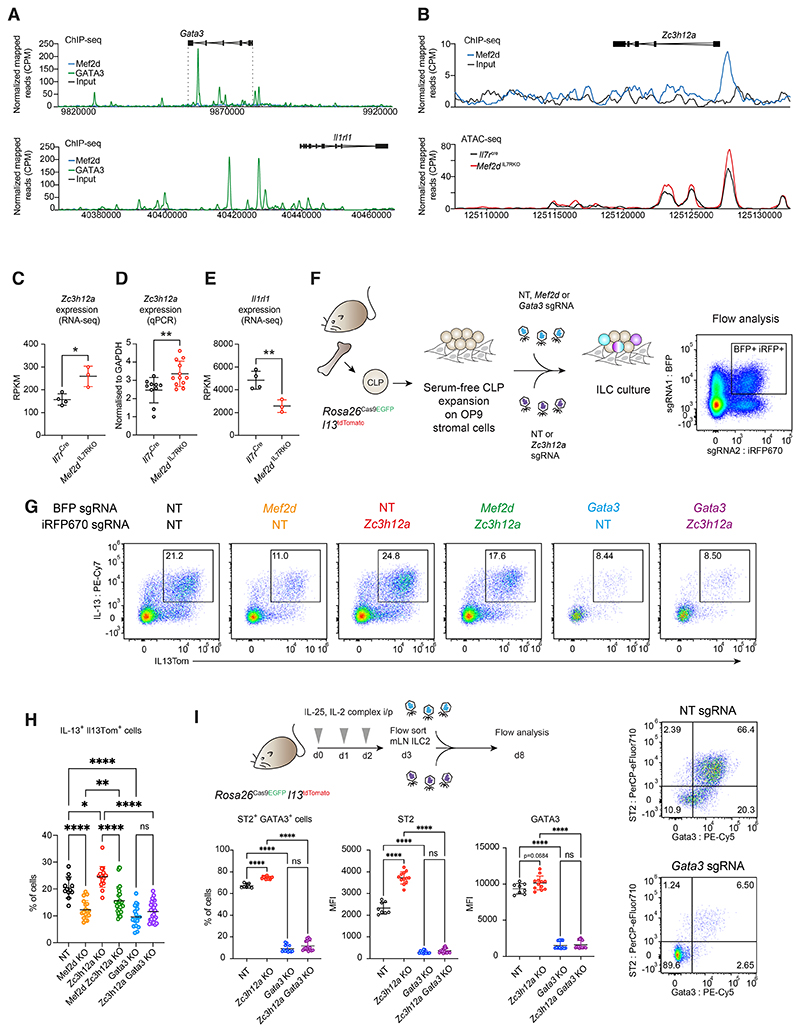
Mef2d represses Regnase-1 transcription to maintain GATA3, ST2, and IL-13 expression. (A) Representative binding profiles of Mef2d and GATA3 in ILC2s at the *Gata3* (top) and *Il1rl1* (bottom) loci. Data representative of 2 biological replicates. (B) Representative binding profiles of Mef2d in ILC2s and ATAC-seq track in *Il7r*^Cre^ or *Mef2d*^IL7RKO^ ILC2s at the *Zc3h12a* locus. Data representative of 2 biological replicates. (C) *Zc3h12a* gene expression (from RNA-seq analysis) in *Il7r*^Cre^ or *Mef2d*^IL7RKO^ ILC2s. Mean ± SD; individual data point denotes biological replicates. (D) *Zc3h12a* gene expression (from qPCR) in *Il7r*^Cre^ or *Mef2d*^IL7RKO^ ILC2s. Mean ± SD; individual data point denotes biological replicates. (E) *Il1rl1* gene expression (from RNA-seq analysis) in *Il7r*^Cre^ or *Mef2d*^IL7RKO^ ILC2s. Mean ± SD; individual data point denotes biological replicates. (F) & (G) Schematic of the experimental procedure to produce single or double *Mef2d, Gata3* and *Zc3h12a* CRISPR-targeted cells in the ILC culture assay by using sgRNA-encoding retroviruses carrying different fluorescent protein reporters and (G) representative flow cytometric plots to identify double CRISPR-KO ILCs. (H) Flow cytometric quantification of the proportion of Il13Tom and IL-13 protein expressing cells transduced with the indicated CRISPR sgRNA as in cells gated in (F) & (G). Data are representative of 2 independent experiments with n=2 biologically independent samples in each experiment and 3 different sgRNAs targeting each gene; individual data point denotes a unique combination of sgRNA pairs; mean ± SD. (I) Schematic of the experimental procedure to produce single or double *Gata3* and *Zc3h12a* CRISPR-targeted ILC2s and representative flow cytometric plots to identify double CRISPR-KO ILC2s. Flow cytometric quantification of ST2, IL-13Tom and GATA3 MFI of ILC2s transduced with the indicated CRISPR sgRNA. Data are representative of 2 independent experiments with n=2 biologically independent samples in each experiment and 3 different sgRNAs targeting each gene; individual data point denotes a unique combination of sgRNA pairs; mean ± SD. Significance was determined using unpaired two-sided t-test [(C) − (E)] or one-way ANOVA with Tukey’s post-hoc test [(H) & (I)]; ns, not significant; *P<0.05; **P<0.01; ***P<0.001; ****P<0.0001.

To investigate how Mef2d regulates GATA3, ST2 and IL-13 expression we performed and cross-referenced RNA-seq gene expression and chromatin immunoprecipitation and sequencing (ChIP-seq) analyses on primary ILC2s purified from control or conditional Mef2d-deficient mice. We identified 1071 upregulated and 863 downregulated genes (fig. S21A) with an enrichment of genes associated with immune-related pathways (fig. S21B). Interestingly, these included a subset of genes which are normally suppressed in ILC2s, e.g. T cell related genes (*Cd3e, Cd3d, Cd247, Il2*), indicative of a repressive role for Mef2d in the regulation of these transcripts (fig. S21B). Mef2d ChIP-seq peaks in ILC2s were enriched for the Mef2 consensus sequence (fig. S21C). Mef2d binding sites were associated with genes regulating asthma and T cell regulation (fig. S21D), and ~20% were located in the vicinity of transcription start sites (TSS) (fig. S21E). Notably, Mef2d did not bind to the *Gata3* or *Il1rl1* loci, indicating that Mef2d does not directly modulate their transcription (Fig. 5A). However, Mef2d bound to the *Zc3h12a* locus (Fig. 5B) which encodes Regnase-1 (also known as Mcpip1) an endoribonuclease that degrades specific mRNA target sequences thereby regulating ILC2 activation (44), GATA3 mRNA degradation and T_H_2 cell-driven inflammation (45). ATAC- seq revealed that the *Zc3h12a* locus was accessible in ILC2s, and that accessibility increased in Mef2d-deficient ILC2s (Fig. 5B). Furthermore, Mef2d-deficiency in ILC2s also resulted in an increase in *Zc3h12a* mRNA expression (Fig. 5C and D).

Stabilization of Regnase-1 in ILC2s, through mutations that block Regnase-1 degradation, results in a reduction in type-2 cytokine expression due to Regnase-1 degrading *Il1rl1* mRNA leading to reduced expression of ST2 and compromised IL-33 signaling (46). Indeed, Mef2d-deficient ILC2s have reduced *Il1rl1* transcripts (Fig. 5E) as well as impaired ST2 protein expression *in vivo* and *in vitro* (see above). Collectively, our data point towards a repressive role for Mef2d in regulating *Zc3h12a* locus accessibility and constraining Regnase-1 expression, which is important for allowing increased production of ST2 and type-2 effector molecules.

To address the relationship between Mef2d, Regnase-1 and GATA3 in type-2 gene regulation, we performed double gene deletion in the same cell during ILC differentiation *ex vivo* (Fig. 5F & G). While individual ablation of Mef2d resulted in a fall in the proportion of IL-13-expressing ILC2s, the single ablation of Regnase-1 increased the percentage of IL-13^+^ ILC2s. Of note, co-deletion of Mef2d and Regnase-1 resulted in a partial rescue of IL-13 producing ILC2s as compared to Mef2d deletion alone (Fig. 5H). These data suggest that both Regnase-1-dependent and -independent pathways downstream of Mef2d exist to regulate ILC2s and IL-13 expression. Finally, co-deletion of GATA3 and Regnase-1 completely reversed the induction effect of Regnase-1 single knockout, down to the level indistinguishable to GATA3 single deficiency, suggesting that the Regnase-1-mediated effects are entirely GATA3-dependent (Fig. 5H).

Similarly, *ex vivo* knockdown of Regnase-1 in *in vivo* stimulated ILC2s resulted in increased ST2 expression and a modest induction of GATA3, which were reversed by co-deletion of GATA3 (Fig. 5I). These data support a role for Mef2d in repressing *Zc3h12a* gene transcription thereby enabling optimal GATA3 and ST2-mediated induction of type-2 cytokine secretion by supporting a feedback loop (54). Notably, we had previously identified Regnase-1 as the strongest negative regulator of T_H_2 cell differentiation in genome-wide screens for IL-13 production (47).

Mef2d-deficient T_H_2 cells elicited in the papain/2W1S model also expressed reduced ST2 and GATA3 (fig. S21F). Like ILC2s, *Zc3h12a* CRISPR-KO increased GATA3 and IL-13Tom expression in T_H_2 cells (fig. S21G) highlighting a shared role for Mef2d in supporting high-level GATA3 expression in innate and adaptive type-2 lymphocytes. MEF2D is also expressed by human ILC2s (fig. S21H) and correlated positively with *GATA3* transcripts (fig. S21I), suggesting that, like mouse ILC2s, *MEF2D* is part of a gene program which includes *GATA3*. Together, our data demonstrate that Mef2d regulation of *Zc3h12a* can modulate GATA3-mediated ILC2 and T_H_2 function. However, they also suggest the existence of an additional Mef2d-dependent function which acts independently to regulate ILC2 function.

### Mef2d is required for calcium-dependent ligand-mediated ILC2 activation and cytokine production

Interestingly, Mef2 proteins are also regulators of calcium signalling. The combination of cytokine (e.g. IL-25 or IL-33) and calcium signalling (downstream of Neuromedin U (NmU) and cysteinyl leukotrienes) in ILC2s can act synergistically to promote ILC2-mediated immune responses (41). These ligands bind to G protein-coupled receptors (GPCRs) which mobilize Ca^2+^ signaling and nuclear factor of activated T cells (NFAT) activation (mirroring the TCR signal in T cells). Consequently, we investigated whether the link between Mef2d and calcium-dependent signaling (48) could explain how co-stimulatory factors such as leukotriene C4 (LTC4) (41, 49) and NmU (50–52), in combination with IL-33 or IL-25, act synergistically to potentiate ILC2 responses. Interestingly, both LTC4 and NmU induce calcium-mediated signaling pathways that mirror the calcium-induced co-stimulation which is activated during TCR or signaling via NFAT in adaptive lymphocytes (41, 49–52), but which are not primarily activated by IL-33, IL-25 or TSLP receptor-induced signaling. Ca^2+^-mediated activation of Mef2d-dependent transcription requires calcineurin to dephosphorylate NFAT1 (encoded by *Nfatc2*) which promotes the formation of Mef2d/NFAT1 transcriptional complexes (53), with NFAT1 reported to be bound to Mef2d upon nuclear translocation (54). Importantly, NFAT1 is a key regulator of GATA3 and type-2 cytokines (55).

**Fig. 6:**
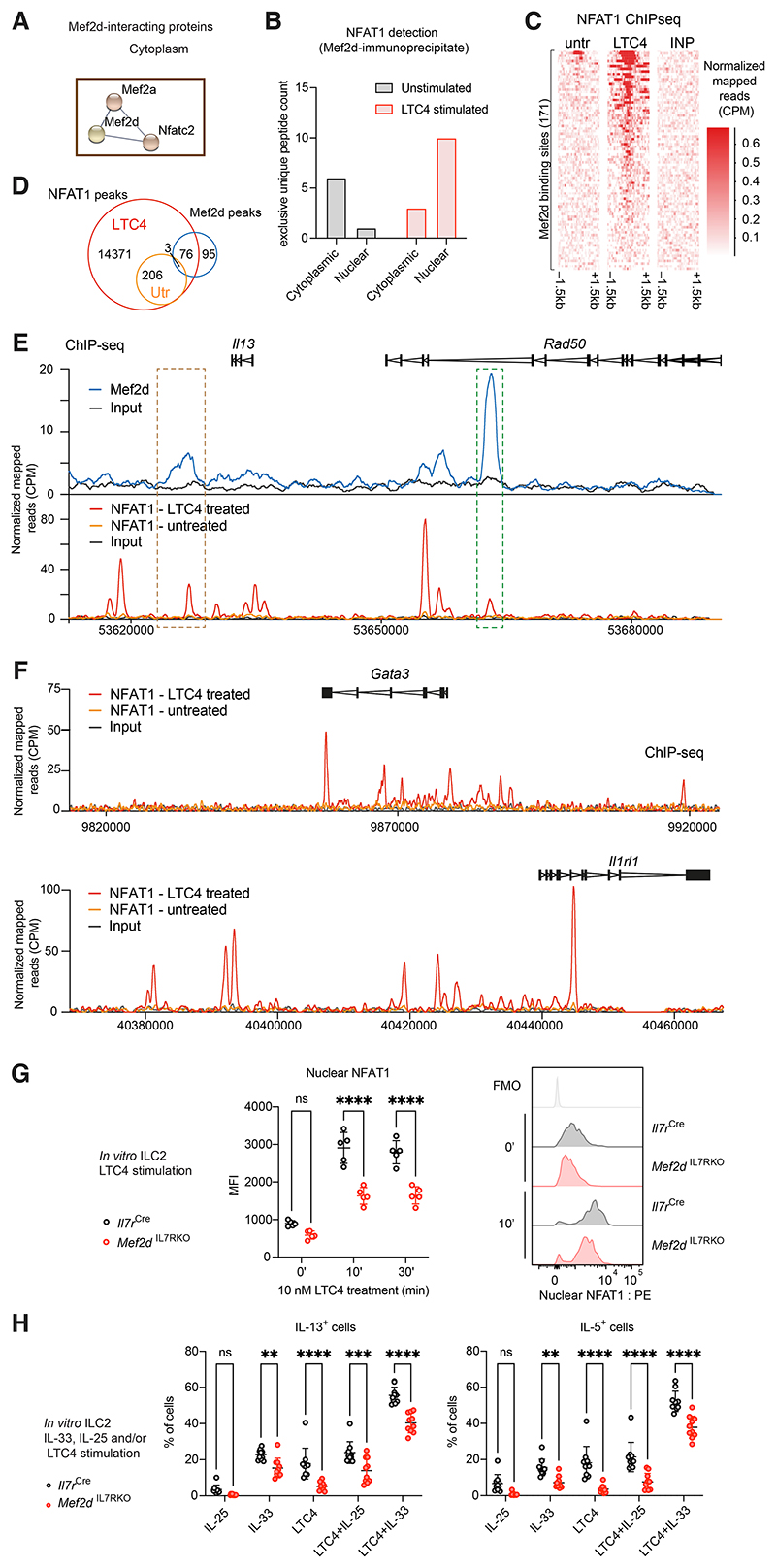
A Mef2d-NFAT1 complex regulates calcium response and synergistic ILC2 cytokine production (A) Identification of NFAT1 and Mef2a as Mef2d-interacting proteins using mass spectrometry of proteins co-immunoprecipitated with anti-Mef2d antibody from ILC2 lysate. The full network of identified interacting proteins is shown in fig. S22. (B) Mass spectrometry quantification of NFAT1 exclusive unique peptide count in Mef2d-immunoprecipitate in the cytoplasm and nucleus of ILC2s before and after LTC4 stimulation. (C) Heatmap representation of NFAT1 binding in ILC2s, with and without LTC4 stimulation, around the centre (±1.5 kb) of Mef2d peaks in ILC2s, ordered according to the LTC4 treated sample. (D) Venn diagram showing the overlap between Mef2d and NFAT1 (LTC4 treated and untreated) ChIP-seq peaks in ILC2s. Peak list was generated using two biological replicates. (E) Representative binding profiles of Mef2d and NFAT1 (LTC4 treated and untreated) in ILC2s at the *Zc3h12a* locus (top) and around the type-2 cytokine LCR region (bottom). Data representative of 2 biological replicates. (F) Representative binding profiles of NFAT1 (LTC4 treated and untreated) in ILC2s at the *Gata3* (top) and *Il1rl1* (bottom) loci. Data representative of 2 biological replicates. (G) Flow cytometric analysis of nuclear NFAT1 MFI of cultured ILC2s following LTC4 treatment at the indicated timepoints. Data are representative of 2 independent experiments with n=5 biologically independent samples in each experiment; mean ± SD. Gating strategy for scatter, singlets and live/dead cell exclusion shown in fig. S23G. (H) Flow cytometric quantification of ILC2 production of IL-13 and IL-5 following stimulation with the indicated molecules. Data are representative of 2 independent experiments with n=5 biologically independent samples in experiment 1 and n=10 biologically independent samples in experiment 2 (depicted here); mean ± SD. Significance in (G) & (H) was determined using unpaired two-sided t-test; ns, not significant; **P<0.01; ***P<0.001; ****P<0.0001; individual data point denotes biological replicates.

Analysis of Mef2d-interacting proteins by immunoprecipitation and mass spectrometry identified NFAT1 and Mef2a in the cytoplasm of ILC2s (Fig. 6A and fig. S22A). LTC4 is a major pro-inflammatory mediator in asthma which signals through CysLT1R via calcium-dependent activation of NFAT (41). To confirm that the Mef2d-NFAT1 cytoplasmic interaction was relevant in the context of calcium signaling, we repeated the Mef2d IP-mass spectrometry analysis following LTC4 stimulation of ILC2s and observed increased Mef2d-NFAT1 interaction in the nucleus (Fig. 6B).

Using NFAT1 ChIP-seq analysis we determined that nuclear NFAT1 binding was robustly induced in ILC2s following LTC4 stimulation (fig. S22B) resulting in enrichment of NFAT motif (fig. S22C). NFAT1-binding peaks were associated with gene pathways involved in T cell regulation/differentiation and asthma (fig. S22D), and more than 30% were localized close to TSS (fig. S22E). Interestingly, cross-referencing NFAT1 and Mef2d ChIP-seq datasets revealed that around 50% of Mef2d peaks were co-bound with NFAT1 upon LTC4 stimulation (Fig. 6C and D). These included the *Rad50* intronic type-2 cytokine locus control region (LCR) and downstream of the *Il13* gene (Fig. 6E). ChIP-seq analyses of Mef2d and NFAT1 binding in T_H_2 cells revealed striking similarities with their ILC2 counterparts (fig. S23A − F), indicative of potentially shared pathways for type-2 gene regulation.

However, NFAT1 also bound to the *Gata3* and *Il1rl1* genes (Fig. 6F and fig. S23F), whereas Mef2d did not (Fig. 5A), indicating additional indirect pathways by which Mef2d may regulate T_H_2 cell and ILC2 differentiation. Indeed, it has been proposed that the association of Mef2d with NFAT1 facilitates its shuttling to the nucleus in response to calcium signaling (53). Therefore, we assessed if the absence of Mef2d altered NFAT1 recruitment to the nucleus of ILC2s and found that the nuclear influx of NFAT1 was markedly reduced in the nuclei of ILC2s from *Mef2d*^IL7RKO^ mice (Fig. 6G, fig. S23G). This deficit in NFAT1 nuclear localization was not due to defective NFAT1 expression, since total NFAT1 protein levels were equivalent between control and Mef2d-deficient ILC2s (fig. S23H). These data are consistent with the requirement for Mef2d in promoting nuclear translocation of NFAT1.

To determine whether Mef2d-dependent NFAT1 nuclear localization was important for ILC2 function we stimulated ILC2s with IL-25 or IL-33, and/or LTC4, and assessed their individual and synergistic induction of IL-13 and IL-5. We found that Mef2d-deficient ILC2s were impaired in their production of IL-5 and IL-13 in response to LTC4 stimulation alone, as well as to LTC4 in combination with IL-33 or IL-25 (Fig. 6H), indicating that Mef2d is required for calcium-induced type-2 cytokine production from ILC2s. Collectively, these data indicate that Mef2d associates with NFAT1 and is required for optimal NFAT1 accumulation in the nucleus and downstream cytokine production following calcium-dependent signaling. Thus, Mef2d regulates both cytokine and calcium-mediated signaling pathways to promote ILC2 function (fig. S24).

## Discussion

ILC2s display gene expression profiles which are adapted to their tissue microenvironments (43) and must be able to process a plethora of signals from their surroundings to help maintain immune homeostasis and respond to injury and infection (56). To identify transcriptional regulators of ILC2 development and function we performed an unbiased CRISPR-Cas9 screen, which has proven challenging in the past due to their rarity. We identified genes with previously unappreciated roles in ILC2 function. These included *Mef2d, Zfp871* and *Nfkb2*. Our screens revealed that Mef2d-deficiency reduced both IL-13 production and GATA3 expression by ILC2s. In contrast, deficiency of *Nfkb2* or *Zfp871* impaired IL-13 but not GATA3 expression. These results suggested that Mef2d acts as an upstream modulator of the type-2 ‘master regulator’ GATA3 in ILC2 differentiation and IL-13 production. Although a number of regulators of IL-13 have been characterized over the years (1, 2), upstream pathways that lead to GATA3 upregulation are less well-characterized, perhaps due to the essential role of GATA3 in defining type-2 lymphocyte identity and the multifaceted functions of GATA3 in upstream lymphocyte development. Low levels of GATA3, induced by Notch signaling, are required to repress B cell fate to initiate thymopoiesis and ILC development (1, 10). In naïve T cells, further upregulation of GATA3 necessitates TCR-, IL-2/STAT5- and IL-4/STAT6-driven pathways to promote T_H_2 over T_H_1/17 fates (2). In contrast, the signals that induce GATA3 expression during ILC development are still unknown, although the IL-33/ST2/p38/phospho-GATA3 signaling axis has been shown to induce further GATA3 upregulation in established ILC2s (42). Furthermore, how enhanced levels of GATA3 are attained specifically in ILC2s relative to other ILCs is still poorly understood. Our results reveal a key role for Mef2d in inducing and sustaining the high levels of GATA3 required for optimum function in type-2 lymphocytes. Indeed, deleting Mef2d in all lymphocytes demonstrated that its absence leads to highly impaired type-2 immunity *in vivo*, but did not alter the maintenance of type-1/17 cytokine-expressing cells which only requires low levels of GATA3. The Mef2d-dependent deficit in type-2 immunity was due to shortfalls in both ILC2- and T_H_2 cell-driven responses. Notably, the effects on T_H_2-dependent phenotypes (using CD4-Cre), are similar to the impairments observed in mice with conditional deletion of GATA3 in *Tnfrsf4*(OX40)-Cre positive cells (57) but possibly not as severe as those reported in Lck-Cre positive cells following GATA3 deletion (58). Indeed, other signaling pathways including STAT6 are known to regulate type-2-permissive GATA3 expression in T cells, and it is possible that other candidate regulators identified in our screens may also contribute to GATA3 expression in ILC2s. However, our results clearly demonstrate a critical role for Mef2d in modulating both T_H_2 cell and ILC2 biology.

To separate the roles of Mef2d within the interwoven ILC2- and T_H_2-dependent immune paths *in vivo* we created BIC mice. To avoid collateral effects in related cells we successfully optimized a cascade of three SSRs to highly restrict Cre expression to the rare ILC2 subset. The BIC mice demonstrate that with careful selection of recombinase driver loci it is possible to engineer multiple levels of gene expression control into the mouse genome to create previously unachievable cell-specificity for synthetic gene-circuit control of gene manipulation *in vivo*. The BIC mice enabled us to mark and flexibly ablate ILC2s at steady-state and during immunological challenge with validation in lung and skin disease models. Further, temporal depletion of ILC2s allowed us to confirm that ILC2s are essential for the launch of T_H_2 cell responses, even in the presence of dendritic cells (22, 23), and demonstrate that Mef2d expression by ILC2s was essential to this process.

Previously, Mef2d has been shown to play roles as both a transcriptional activator and suppressor, but its role in regulating the functions of type-2 lymphocytes has not been explored. Our data pointed away from Mef2d directly binding to the *Gata3* locus, but instead, suggested an indirect mechanism for it promoting GATA3 expression. Indeed, Mef2d bound and repressed the expression of Regnase-1, an RNA-binding protein with known functions as an endonuclease in the degradation of mRNA encoding immunoregulatory molecules including *Gata3* and *Il1rl1* transcripts (44–46, 59–61). We observed impaired expression of ST2 (*Il1rl1*) when Mef2d was deleted in ILC2s and was therefore not available to repress Regnase-1 production. This resulted in impaired responses to IL-33 stimulation including decreased p38 activation and GATA3 phosphorylation which are known to be critical for ILC2 function (42). Our findings are in line with studies using Regnase-1-deficient mice (45), and mice in which Regnase-1 has been mutated (*Regnase-1*^AA/AA^ mice) to reduce its clearance via the IkB complex-mediated degradation (46), which have demonstrated roles for Regnase-1 in suppressing ILC2 and T_H_2 cell biology. In Regnase-1-deficient ILC2s and T_H_2 cells there is an enrichment in *Gata3* transcripts, which in T cells is due to the RNase domain of Regnase-1 (45). This correlated with increases in type-2 responses (46), and in the ILC2 study, pulmonary fibrosis (59). In *Regnase-1*^AA/AA^ mice the impaired decay of Regnase-1 results in its accumulation which leads to increased degradation of *Il1rl1* transcripts (59). Thus, our results identified a previously unappreciated role for Mef2d in repressing Regnase-1 expression, thereby preventing Regnase-1-mediated degradation of *Gata3* and *Il1rl1* mRNA and promoting a type-2 immune program through the promotion of ST2-mediated IL-33 signaling and the accumulation of GATA3. Interestingly, we found that in the intestine, where IL-25-responsive ILC2s predominate, the Mef2d/Regnase-1/GATA3/ST2/IL-33 was not critical, supporting the previously proposed tissue specialization of microenvironment-modified ILC2s (43). Our data suggest that Mef2d preferentially drives the ST2^+^ phenotype associated with lung ILC2s, thereby permitting their rapid response to the alarmin IL-33 during allergen exposure, and promoting a Mef2d-dependent signaling loop. By contrast, our results suggest that the unique microenvironment of the intestinal lamina propria may provide distinctive alternative signals, for example those derived from the microbiota and intestinal stroma, which modulate GATA3 expression in gut ILC2s and which appear less reliant on the Mef2d feedback loop. Indeed, it is possible that additional candidates identified in our CRISPR screen may be involved in alternatively modulating GATA3 in ILC2s from other tissue microenvironments.

Interestingly, Mef2 proteins can also act as calcium-dependent regulators of cell differentiation and function through the modulation of NFAT transcription factors (48, 53, 62, 63). Indeed, parallels have been drawn between TCR-induced calcium signaling in T cells and GPCR-Gαq-induced calcium signaling in ILC2s, both of which stimulate cytokine production through NFAT activation and nuclear localization (41). Despite the key roles of calcium-inducing mediators in ILC2 effector function, regulators of this pathway have not been extensively studied beyond the canonical calcium signaling proteins. Here we determined that Mef2d binds to NFAT1 permitting efficient localization of NFAT1 to the nucleus where it can promote transcription of genes including *Gata3, Il1rl1* and the type-2 cytokine gene cluster (55, 64). In ILC2s this pathway lies downstream of potent ILC2-stimulators including LTC4 (41, 49) and NmU (50–52) which synergize with cytokine activation to potentiate type-2 immune responses, mirroring calcium-mediated signaling pathways downstream of the TCR in T cells.

Our preliminary analyses suggested a potentially conserved pathway of Mef2d-mediated GATA3 expression in human ILC2s. Further analyses are warranted to elucidate the effect of Mef2d inhibition on GATA3 and type-2 gene expression in human cells, and to explore the therapeutic value of Mef2d inhibitors. Our study has highlighted a critical role for Mef2d in licensing type-2-permissive high level GATA3 expression, via Regnase-1 inhibition, in both ILC2s and T_H_2 cells to support optimal type-2 immunity *in vivo*. We have further demonstrated that Mef2d in ILC2s acts prominently in both the IL-33 cytokine-stimulated pathway and the LTC4-induced calcium dependent pathway which converge to control ILC2 proliferation and cytokine production. These pathways combine in positive feedback loops to reinforce type-2 immune responses. Thus, by combining CRISPR-screens with sophisticated Boolean mouse models we have successfully defined and characterized candidate genes and their inter-related roles in the regulation of closely related immune cell subsets at previously unachievable resolution. Whilst we have applied the BIC line to definitively establish the critical role of ILC2s in promoting the initiation of T_H_2-driven adaptive immunity, the validation of this intersectional SSR approach has broad value for investigators with the growing complexity of cell sub-types that are being defined especially with the advent of single cell analysis technologies.

## Materials and methods

### Mice

*Rosa26*^Cas9EGFP^ (JAX 026179) (65), *Il13*^tdTom^ (66), *Ilr7*^Cre (67)^, *Cd4*^Cre^ (Taconic, model #4196), 5x polychromILC mice, *Rora*^Teal^, *Bcl11b*^tdTom^ and *Id2*^BFP^, *Gata3*^hCD2^, *Rorc*^Kat^ (68), *Tbx21*^hCD4^ (69) mice were on the C57BL/6 background. C57BL/6 controls were bred in-house. *Mef2d*^fl^ mice were provided by the RIKEN BRC through the National BioResource Project of the MEXT/AMED, Japan. ROSA-tdRFP mice (MGI allele: Gt(ROSA)26Sor^tm1Hjf^ (70)) were a kind gift of Hans Jörg Fehling. ROSA26iDTR mice (MGI allele: C57BL/6-Gt(ROSA)26Sortm1(HBEGF)Awai/J) were from The Jackson Laboratory (007900). CD45.1 *Rag2*^-/-^*Il2rgc*^-/-^ mice were a gift from James Di Santo. Detailed information on the generation of *Icos*^Cre^, *Il13*^Dre^ and *Cd28*^Vika^ alleles are provided in the Supplementary Materials and Table S1. All mice were maintained in the Medical Research Council ARES animal facility under specific pathogen-free conditions, at 19-23°C, 45-65% humidity, with a 12-h light-dark cycle. In individual experiments, mice were matched for age (6-12 weeks), sex and background strain and all experiments undertaken in this study were done so with the approval of the Laboratory of Molecular Biology Animal Welfare and Ethical Review Body (AWERB) and of the UK Home Office. Mice were euthanised by gradual exposure to CO2 followed by either cervical dislocation or exsanguination.

### *In vivo* stimulation

In the IL-33-elicited lung inflammation model, mice were anesthetized by isoflurane inhalation followed by the intranasal injection of 250 ng recombinant mouse (rm)IL-33 (BioLegend, #580508) on days 0, 1 and 2 then sacrificed for analysis on day 3.

In the *A. alternata* extract-elicited lung inflammation model, mice were anesthetized by isoflurane inhalation followed by the intranasal injection of 10 mg *A. alternata* extract (Stallergenes Greer, #My1) on days 0, 1 and 2 then sacrificed for analysis on day 3.

In the *Alternaria alternata* skin inflammation model mice were treated by intradermal injection of 10 mg of *A. alternata* extract (Stallergenes Greer, #My1) in 10 ml of PBS into the right ear and 10 ml of PBS only into the left ear on days 0, 1 and 2 and then sacrificed for analysis on day 3.

In the recall challenges with either Papain only or Papain and 2W1S-antigen mice were anesthetized by isoflurane inhalation followed by the intranasal injection of either with 12.5 mg papain (Sigma-Aldrich, #76216) or 2W1S peptide (50 mg, Designer Bioscience) in combination with 12.5 mg papain (Sigma-Aldrich, #76216) in 40 ml PBS on days 0 and 14. Mice were sacrificed for analysis on day 19.

For calcipotriol treatment, mice were topically applied with nmol calcipotriol (20 mL) on the right ear, and 20 ml ethanol vehicle control on the left ear each day. Dosing was performed for 3 consecutive days, followed by 2 days rest, and then for another 3 consecutive days. Ear thickness measurements were taken on the day following the last dose.

For the helminth infection model mice were inoculated subcutaneously with 500 viable third-stage *N. brasiliensis* larvae on day 0 and mice were sacrificed for analysis on day 8.

In the *Citrobacter rodentium* infection model mice were inoculated with 10^9^ CFU of *C.rodentium* by oral gavage on day 0 and mice were sacrificed for analysis on day 5.

To mediate ILC2 ablation using Diptheria toxin (DTx) the toxin (20 ng/g body weight, Sigma) was administered daily for 3 or 4 consecutive days intraperitonally (as indicated in specific treatment schematics). In the case of stimulation experiments DTx treatment was started one day prior to the respective treatment.

To induce sufficient numbers of ILC2s for *in vitro* expansion, mice were injected intraperitoneally with 1 mg rmIL-25 (Janssen) and rmIL-2 (BioLegend, #575406) complexed with a-IL-2 antibody (2B Scientific, Clone JES6-1A12, #BE0043) on days 0, 1 and 2, then mesenteric lymph nodes were harvested on day 4 to purify ILC2s by flow cytometry.

### Adoptive cell transfer

Cultured ILC2 (defined as GATA3^+^ Tbet^-^) and NK/ILC1 (defined as GATA3^-^ Tbet^+^) from the ILC culture were purified by flow cytometry and implanted via tail vein injection into sublethally irradiated (450 rad) *Rag2*^-/-^ *Il2rgc*^-/-^ recipients. Analysis of donor cell progeny was performed 2 weeks after cell transfer.

### Tissue preparation

Cell suspensions from spleen, lymph nodes, liver and thymus tissue were obtained by passing the tissues through a 70-mm strainer. Lung tissue was predigested with 750 U ml^−1^ collagenase I (Gibco) and 0.3 mg ml^−1^ DNaseI (Sigma-Aldrich) before obtaining a single-cell suspension. Bone marrow was removed from femurs and tibiae by flushing with PBS, 2% FCS or by centrifuging briefly at 6,000*g*. For bone marrow, lung, liver and spleen cell suspensions, red blood cells were removed by incubating with RBC lysis solution (140 mM NH_4_Cl, 17 mM Tris, pH 7.2). Lung and liver lymphocytes were further enriched by centrifugation in 30% or 40% Percoll respectively at 800*g* (GE Healthcare).

For preparation of siLP and cLP lymphocytes, intestinal contents were removed by the application of gentle pressure along the length of the intestine. Intestines were opened longitudinally, cut into 3 cm long pieces and washed briefly by vortexing in PBS + 10 mM HEPES (PBS/HEPES). Epithelial cells were removed by incubation with RPMI supplemented with 2% FCS, 1 mM dithiothreitol and 5 mM EDTA for 2 x 20 mins at 37°C with shaking (200 rpm). Intestinal pieces were washed with PBS/HEPES and incubated, with shaking, at 37°C with RPMI + 2% FCS, 0.125 KU/ml DNaseI (Sigma-Aldrich) and 62.5 □g/ml Liberase TL (Roche) until no large pieces of intestine remained. Cells were then passed through a 70 □m strainer, pelleted and separated over a 40%:80% gradient of Percoll at 600 x g for 20 minutes. siLP and cLP lymphocytes were isolated from the interface and prepared for flow cytometric analysis. Unless stated otherwise, small intestine lamina propria (siLP) and colonic lamina propria (cLP) include associated Peyer’s patches.

Cell suspensions from adipose tissue were obtained by mechanical dissociation in RPMI-1640, and digested with collagenase I (Life Technologies), DNase I (Roche) at 37°C whilst shaking. Initial wash steps were performed with PBS 3% FCS warmed to 37°C and centrifugation steps (400 x g) were performed at room temperature to allow separation of the cell pellet from the fat.

Skin cell suspensions were obtained from the ear. Hair was removed from the ears using depilatory cream which was wiped away with tissue after four minutes. Ears were then washed three times in PBS 3% FCS before separating the dorsal and ventral halves. The skin pieces were then minced using scissors in RPMI containing 10% FCS, 0.4mg/ml Liberase TM and 60 ng/ml DNaseI and incubated with mixing for 30 minutes at 37°C. Digested tissue was then passed through a 70 □m strainer and centrifuged at 800 x g and skin lymphocytes were further enriched by centrifugation in 30% Percoll at 900 x g (GE Healthcare).

### Flow cytometry

Single-cell suspensions were incubated with fluorochrome- or biotin-conjugated antibodies (full list in Supplementary Table S2) in the presence of anti-CD16/CD32 (Fc block, clone 2.4G2) and a cell viability dye. ‘Lineage’ staining for each experiment is defined in the relevant figure legends. Analysis was performed on an LSRFortessa system (BD Biosciences) with FACSDiva software (version 6.2, BD Biosciences) or an ID7000 spectral cytometer (Sony Biotechnology). For cell sorting, an iCyt Synergy system (70-μm nozzle, Sony Biotechnology) was used. Intracellular cytokine staining was performed using BD Cytofix/Cytoperm Plus reagents (BD Biosciences) following pre-culture with RPMI, supplemented with 50 ng ml-1 phorbol 12-myristate 13-acetate (PMA), 500 ng ml-1 ionomycin and Protein Transport Inhibitor Cocktail (eBioscience), for 4 h at 37°C. In in vitro experiments measuring IL-25/IL-33/LTC4-induced cytokine expression, ILC2s were cultured with the indicated molecules in RPMI supplemented with Protein Transport Inhibitor Cocktail for 4 h at 37oC before surface staining and fixation with the BD Cytofix/Cytoperm Plus reagent and intracellular cytokine staining. Intracellular TF staining was performed using Foxp3 Staining kit reagents (eBioscience). In some experiments where samples were simultaneously stained for intracellular cytokine and TF, the Foxp3 staining kit was used. Fixation of samples containing fluorescent proteins was performed with 2% PFA at room temperature for 45 minutes. Intracellular phospho-protein staining was performed by fixation with 2% PFA for 15 min, overnight permeabilization with 90% methanol at -20oC, followed by incubation with fluorochrome antibodies diluted in 2% BSA PBS. In cell trace violet dilution experiments, purified ILC2s were labelled with cell trace violet (Invitrogen, #C34557) according to the manufacturer’s instructions prior to culture. Flow cytometric analysis of nuclear Nfat1 was performed as described previously (71). Briefly, cells were lysed to remove cytoplasmic membrane followed by nuclei fixation and staining with PE-conjugated anti-Nfat1 antibody. Data were analyzed with FlowJo software (version 10). Mean fluorescence intensity (MFI) values presented and compared within data plots are from the same experiment, and not compared between different experiments.

### sgRNA cloning into retroviral expression vector

MSCV-pU6-(BbsI)-CcdB-(BbsI)-Pgk-Puro-T2A-BFP was a gift from Ralf Kuehn (Addgene plasmid # 86457; http://n2t.net/addgene:86457; RRID: Addgene_86457) (72). Custom sgRNA libraries were synthesised by Twist Bioscience as described previously (73). sgRNA libraries were cloned into the retroviral vector by Gibson assembly. sgRNA library representation was verified by next generation sequencing to contain > 90% perfectly matching sgRNAs, < 0.5% undetected sgRNAs and a skew ratio of less than 10. sgRNA oligo pairs were purchased from Sigma-Aldrich. Individual CRISPR sequences were inserted into the retroviral vector by ligation (NEB T4 DNA ligase). Sequences of individual sgRNA-expressing constructs were confirmed by Sanger sequencing.

### Retroviral production

Platinum-E retroviral packaging cells (Cell biolabs, #RV-101) were maintained in DMEM, 10% FCS with penicillin-streptomycin, supplemented with puromycin (1 mg ml^-1^) and blasticidin (10 mg ml^-1^). On the day before transfection, 3 million cells were seeded in a 100 mm culture dish in 10 ml of media without antibiotics. Cells were transfected at 70% confluency using Fugene HD Transfection Reagent (Promega). For each 100 mm culture dish, 950 ml OPTI-MEM (GIBCO) was mixed with 11 mg pCl-Eco, 22 mg library plasmid and 99 ml Fugene HD. The transfection mixture was incubated for 10 min at room temperature prior to addition. At 18 h post-transfection, the media was replaced with 10 ml fresh media, and viral supernatant was harvested at 48 and 72 h post-transfection. Cells were removed by filtering through a 0.45 mm syringe filter.

### ILC culture for CRISPR screening

OP9 and OP9-DL cells were obtained from the Sunnybrook Research Institute (74) and maintained in IMDM supplemented with 20% FCS with 1% penicillin-streptomycin, 50 mM 2-mercaptoethanol and 0.1% non-essential amino acid (complete IMDM). Prior to co-culture with lymphocytes, OP9 and OP9-DL cells were incubated with 4 mg/mL mitomycin C for 2 h, washed, and seeded at a density of 1 million cells per 96-well plate and allowed to adhere. Bone marrow common lymphoid progenitors (CLPs) were sorted as live CD45^+^ Lin^-^ IL-7Ra^+^ Flt3^+^ Ly6D^-^ cells from *Rosa26*^Cas9EGFP^ x *Il13*^tdTom^ mice or *Rosa26*^Cas9EGFP^ x *Gata3*^hCD2^ x *Tbx21*^hCD4^ x *Rorc*^Kat^ mice. For CLP expansion, purified CLPs were co-cultured with OP9 cells for 6 days in serum-free IMDM supplemented with 25 ng/mL rmFlt3L (BioLegend, #550702) and 0.1 ng/mL rmIL-7 as described previously (75). At day 6, expanded CLPs were collected and flow sorted for viable CD45^+^ CD19^-^ cells and mixed with retroviruses and spinoculated on retronectin-coated plates (Takara, 4 mg/cm, non-TC-treated plate) at 37oC for 1 h. Cells were incubated further for 3 h at 37oC before being transferred to co-culture with OP9-DL cells, in complete IMDM supplemented with 10 ng/mL rmIL-7 for the next 6 days. At day 12, cells and collected and transferred to OP9 cells in complete IMDM supplemented with 25 ng/mL rmIL-7 and rmSCF (BioLegend, #579702) for the next 6 days. At day 18, GFP+ BFP+ cells were sorted into populations of interest discernible by reporter protein expression. In a typical screen, 200,000 CLPs were purified from the femur, tibia and ilia of 20 mice at d0, which would expand to 5-10 million cultured CLPs at d6 prior to transduction. Cells would continue to proliferate during the ILC culture until day 18, at which point 4 million cells from each population were flow purified according to the reporter allele expression for sgRNA analyses.

### Genomic extraction and sequencing library preparation

Genomic DNA from sorted cells were extracted using the QIAGEN DNeasy Blood & Tissue Kits following the manufacturer’s protocol, with the exception of DNA elution in water instead of buffer AE. sgRNA-insert was first PCR-amplified using Herculase II Fusion DNA polymerase (Agilent) with primers (Forward) AATGGACTATCATATGCTTACCGTAACTTGAAAGTATTTCG and (Reverse) CTTTAGTTTGTATGTCTGTTGCTATTATGTCTACTATTCTTTCC, using up to 2 mg genomic DNA per 50 ml reaction. Equal volumes from each reaction were pooled and used for a further PCR amplification step to attach Illumina sequencing adaptors and Illumina P7 barcodes, using Herculase II Fusion DNA polymerase. The 330 bp library was gel purified and quantified using KAPA library quantification kit (Roche). Libraries were pooled and sequenced with a HiSeq 4000 at the CRUK Cambridge NGS facility.

### Analysis of CRISPR screen results

20 nt sgRNA sequences were trimmed from backbone sequences using Cutadapt (version 1.4.1) (5’ GACGAAACACCG, 3’ GTTTTAGAGCTA). sgRNA sequences were aligned to reference sgRNA libraries using Bowtie2 (version 1.2.3). sgRNAs with counts less than 20 (genome-wide screens) or 50 (all other screens) in either of the populations were excluded from the analysis. The stat.wilcox function from the caRpools package (version 0.83) was applied to each screen separately using R (v4.1.1). The function was modified to return the non-adjusted p-values. The stat.wilcox function collapses the sgRNAs to genes returning an enrichment score and a p-value for each gene. NT sgRNAs were used as a reference population. To combine data from screen replicates, the mean of enrichment score for each gene was calculated, and Fisher’s method was used to combine the p-values.

### Western Blot

Protein lysates in RIPA buffer were denatured by boiling at 95°C for 5 min in 1X NuPage LDS sample buffer (#NP0008) supplemented with 1% 2-mercaptoethanol. Proteins were resolved with Novex Tris-Glycine gels and transferred to PVDF membranes. Membranes were sequentially blocked with 5% BSA in PBST, incubated with primary and HRP-conjugated secondary antibodies and ECL western blotting detection reagent (GE Healthcare #RPN2106). Mef2d antibody was purchased from BD (#610774, 1:5000 using 5% BSA as blocking buffer).

### *In vitro* mouse T_H_2 cell culture

For differentiation assays splenic naïve CD4^+^ T cells were sorted as CD4^+^ CD44^lo^ CD62L^hi^ CD25^−^. Cells were maintained in RPMI1640, 10% FCS with penicillin-streptomycin and 2-mercaptoethanol. 200,000 naïve CD4^+^ T cells per well were cultured on anti-CD3 coated plates (2B Scientific, 145-2C11, 5 mg ml^-1^), supplemented with anti-CD28 (2B Scientific, 37.51, 2 mg ml^-1^), IL-2 (BioLegend, 575406, 10ng ml^-1^), IL-4 (Biolegend, 574306, 10ng ml^-1^) and anti-IFN-γ neutralising antibody (BioLegend, 11B11, 1 mg ml^-1^). Cells were stimulated for 6 days (day 0-6), then rested for 8 days (day 6-14), restimulated or 3 days (day 14-17) and harvested for analysis by flow cytometry on day 17.

### *In vitro* mouse ILC2 culture and stimulation

Flow purified mesenteric lymph node ILC2s (Viable Lin-ICOS+ KLRG1+ cells) were maintained in RPMI 1640, 10% FCS with penicillin-streptomycin and 2-mercaptoethanol, supplemented with IL-2 (10 ng/mL), IL-7 (10 ng/mL, BioLegend, #577802) and IL-33 (10 ng/mL) for 6 days. For subsequent cytokine/LTC4 stimulation, expanded ILC2s were rested for 1 d in 10 ng/mL IL-2 and IL-7, then stimulated with the indicated cytokines and/or mediators: IL-25 (10 ng/mL), IL-33 (10 ng/mL), LTC4 (10 nM, Cambridge bioscience #CAY20210-25ug). The ILC2 Bl6 cell line of C57Bl6 origin (Qi Yang (76)) was maintained in alpha-MEM supplemented with 20% FCS and 10 ng/mL of IL-2, IL-7 and IL-33.

### Human ILC2 isolation and culture

UK HRA approval was granted following Research Ethics Committee (North West-Liverpool Central) review and written consent obtained from volunteers (1 male and 4 females, age range 26-66). Human peripheral blood ILC2s were isolated from healthy volunteers and severe asthmatics using the MACS human ILC2 Isolation Kit (Miltenyi Biotec, #130-114-825) according to the manufacturer’s instructions. In a typical experiment, around 3,000 ILC2s were obtained from 50 mLs of peripheral blood. Purified human ILC2s were cultured in the presence of recombinant human (rh)IL-2 (10 ng/mL, 202-IL-010), rhIL-7 (10 ng/mL, BioLegend 581908), rhIL-18 (10 ng/mL, BioLegend 592102), rhIL-25 (10 ng/mL, R&D 1258-IL-025/CF) and rhIL-33 (10 ng/mL, BioLegend 581802) for 14 days. ILC2 purity and identity were confirmed by flow cytometric analyses of lineage markers, including CD3 and CD4 negativity, and GATA3 positivity. After cell expansion, ILC2s were rested for 3 days in the presence of rhIL-2 and rhIL-7 (10 ng/mL each), followed by stimulation with the additional indicated cytokines for 3 days: a) basal condition − IL-2 and IL-7 only, b) basal plus IL-18, IL-25 IL-33, c) basal plus rh-IL-4 (50 ng/mL, R&D 204-IL-010), d) basal plus rh-IL-12 (50 ng/mL, R&D 219-IL-005), e) basal plus rh-IFN-γ (50 ng/mL, BioLegend 570202). Cells were harvested for flow cytometric and transcript analyses.

### RNA-sequencing

Cells were sorted by flow cytometry into PBS, 50% FCS, and RNA was extracted using the RNeasy Plus Micro kit (Qiagen). After assessment using a Bioanalyzer (Agilent), RNA was processed for RNA-seq using an Ovation RNA-seq System V2 (Nugen), fragmented using the Covaris M220 ultrasonicator and bar-coded using Ovation Ultralow Library Systems (Nugen). Samples were sequenced using an Illumina HiSeq 4000, by running a single-read 50-bp protocol (Cancer Research UK Cambridge Institute). Sequence data were trimmed to remove adaptors and sequences with a quality score below 30 using Trim Galore (version 0.50, Babraham Bioinformatics) and then aligned to the mouse genome (GRCm38) using STAR (version 2.6.0a), and differential expression was calculated using DESeq2 (version 1.18.1) (77).

### RT-qPCR

RNA was purified using QIAGEN RNeasy Mini Kit. cDNA synthesis was performed using SuperScript IV Reverse Transcriptase (Invitrogen). Diluted cDNA (1:20) was used as template for Taqman qPCR assays. The following probes for mouse genes were used: mouse *Zc3h12a* Thermo Fisher probe assay ID Mm00462535_g1, mouse *Nmur1* probe assay ID Mm00515885_m1, mouse *Gapdh* probe #4352932E, Applied Biosystems. The following probes for human genes were obtained from Thermo Fisher: Human *Mef2d* (assay ID Hs00954735_m1), human *Zc3h12a* (assay ID Hs00962356_m1), human *Gata3* (assay ID Hs00231122_m1), human *Il1rl1* (assay ID Hs00249384_m1), human *Il13* (assay ID Hs00174379_m1), human *Tbx21* (assay ID Hs00203436_m1), human *Foxp3* (assay ID Hs01085834_m1), human *Il10* (assay ID Hs00961622_m1), human HPRT1 (#4333768F, Applied Biosystems).

### Immunoprecipitation

*In vitro* expanded ILC2s (ILC2 Bl6 cell line) were lysed in lysis buffer (50 mM Tris pH 8.0, 0.1% NP40, 10% glycerol and 2 mM EDTA), supplemented with 1x cOmplete protease inhibitor (Roche) and PMSF (Sigma Aldrich). After 10 min incubation on ice with intermittent mixing the lysates were centrifuged at 1,700 g at 4oC for 5 min and the supernatant was collected. The pelleted nuclei were resuspended in nuclear extraction buffer (50 mM Tris pH 8.0, 1 mM EDTA, 150 mM NaCl, 1% NP40 and 5% glycerol) supplemented with protease inhibitor cocktail and PMSF, and incubated on ice for 1 hour. Nuclear extract was collected by centrifugation at 13,000 x g at 4oC for 10 min. Protein concentration was quantified using the Pierce 660nm protein assay reagent (ThermoFisher, #22660). Lysates were incubated with antibodies (2 mg antibody per 100 mg protein) overnight at 4oC on a rotator. Immunocomplexes were precipitated with protein A/G dynabeads (Thermo Scientific #88802), washed three times with lysis buffer and once with TE buffer (10 mM Tris and 0.1 mM EDTA, pH 8). For mass spectrometry analysis, the immunocomplexes were resuspended in 50mM NH4HCO3 followed by reduction with 10 mM DTT and alkylation with 55mM iodoacetamide. Then, proteins were digested (50 mM (NH_4_)HCO_3_ pH 8.0, 1 μg trypsin, overnight, 37°C). Digestion was terminated by the addition of formic acid to a final concentration of 2% v/v. After separation (C18 Acclaim PepMap100 3 μm, 75 μm x 150 mm nanoViper, ThermoScientific Dionex, San Jose, USA), peptides were eluted with a gradient of acetonitrile. The analytical column outlet was directly interfaced via a modified nano-flow electrospray ionisation source, with a hybrid dual pressure linear ion trap mass spectrometer (Orbitrap Velos, ThermoScientific, San Jose, USA). Data dependent analysis was carried out, using a resolution of 30,000 for the full MS spectrum, followed by ten MS/MS spectra in the linear ion trap. MS spectra were collected over a m/z range of 300−2000. MS/MS scans were collected using a threshold energy of 35 for collision induced dissociation. LC-MS/MS data were then searched against a protein database (UniProt KB) using the Mascot search engine programme (Matrix Science, UK) (78). Database search parameters were set with a precursor tolerance of 5 ppm and a fragment ion mass tolerance of 0.8 Da. Two missed enzyme cleavages were allowed and variable modifications for oxidized methionine, carbamidomethyl cysteine, pyroglutamic acid, phosphorylated serine, threonine and tyrosine were included. MS/MS data were validated using the Scaffold programme (Proteome Software Inc., USA) (79). All data were additionally interrogated manually.

### ChIP-seq using ChIPmentation

Chromatin extracts from *in vitro* expanded ILC2s (1.0 × 10^7^ cells) were prepared using the truChIP Chromatin Shearing kit (Covaris), with 5 min of crosslinking and optimized shearing conditions (peak power, 75; duty factor, 10.0; cycles per burst, 200; duration, 300 s), to obtain fragments of ~500 bp. Extracts were exposed to 1% SDS and diluted 10x with dilution buffer (5.5 mM EDTA, 55 mM Tris-HCl, pH 8, 200 mM NaCl, 0.5% NP-40). Chromatin extracts were incubated overnight at 4 °C with 2 μg of antibody. In addition, 25 μl protein A Dynabeads (Thermo Fisher Scientific) per immunoprecipitation were blocked in PBS containing 0.1% BSA (Sigma) by incubating overnight at 4 °C. The next day, beads were added to the chromatin extracts, followed by incubating for 1 h at 4 °C. Beads were collected and washed twice with low-salt buffer (0.1% SDS, 1% Triton X-100, 1 mM EDTA, 10 mM Tris-HCl, pH 8, 140 mM NaCl, 0.1% sodium deoxycholate), twice with high-salt buffer (0.1% SDS, 1% Triton X-100, 1 mM EDTA, 10 mM Tris-HCl, pH 8, 500 mM NaCl, 0.1% sodium deoxycholate), twice with LiCl buffer (10 mM Tris-HCl, pH 8, 1 mM EDTA, 250 mM LiCl, 0.5% NP-40, 0.5% sodium deoxycholate) and once with 10 mM Tris-HCl, pH 8. Chromatin−antibody−bead complexes were then subjected to tagmentation, followed by the elution of DNA, and libraries were amplified and purified as described previously (80). Pooled libraries were sequenced using an Illumina HiSeq 4000, running a single-read 50-bp protocol (Cancer Research UK Cambridge Institute). Sequenced reads were aligned to the mouse genome (GRCm38) using Bowtie2 (version 2.3.5.1) with default parameters, and reads that could not be uniquely mapped were removed from further analyses. Aligned reads were visualised using the SeqMonk software (v1.48.0). HOMER (81) (v4.10.4) software was used for motif find analysis. Peak calling analysis was performed using Macs2 (v2.1.2) and the target genes were defined by the closest gene from each peak (bedtools closest). Only target genes identified in two independent experiments were used in further analysis.

### ATAC-seq

ATAC-seq was performed as previously described (82). 20,000 to 50,000 FACS purified cells were lysed using cold lysis buffer (10 mM Tris-HCl, pH 7.4, 10 mM NaCl, 3 mM MgCl2 and 0.1% NP-40) to obtain nuclei extract. Nuclei were immediately used in the transposase reaction (25 μl 2× TD buffer, 2.5 μl transposase (Illumina) and 22.5 μl nuclease-free water) for 30 min at 37 °C, followed by sample purification (Qiagen MinElute kit). Then, we amplified library fragments using Kappa HiFi HotStart Ready mix and 1.25 M of custom Nextera PCR primers as previously described (83). Libraries were purified using dual (0.5x-0.7x) SPRI Ampure XP beads (Beckman Coulter), pooled and were subjected to high-throughput sequencing. ATAC-seq data was aligned to the genome using the same pipeline as the ChIP-seq data.

### H&E inflammation scoring

Formalin-fixed lung tissue were processed for histological staining by the Cambridge University Hospital Tissue Bank. H&E and periodic acid Schiff (PAS) stained slides were scored by a blinded researcher. Specimens were initially ranked by inflammation (size and cellularity of inflammatory infiltrates) as well as goblet cell metaplasia, and then scored on a severity index of 1 (least inflammation) to 10 (worst inflammation).

### Statistical analysis

Statistical analysis was performed using GraphPad Prism version 9 software. Data are plotted as mean with SD error bars. Statistical significance was calculated by unpaired Student’s t-test (two-tailed), one-way or two-way ANOVA. ****: P<0.0001, ***: P<0.001, **: P<0.01, *: P<0.05, ns: not significant.

## Supplementary Material

Supplementary Materials Combined

## Data Availability

All data needed to evaluate the conclusions in the paper are available in the main text or the supplementary materials. All high-throughput data in this study were deposited at the Gene Expression Omnibus (GEO) under accession number GSE242147. The BIC mice are available from Andrew McKenzie under a material agreement with UK Research and Innovation.
