# Supplementary material for: Mef2d potentiates type-2 immune responses and allergic lung inflammation Authors:
Source: Science. Author manuscript; Available in PMC 2024 Jul 17. (PMC7616247; doi:10.1126/science.adl0370)
Supplement: Supplementary Materials Combined [file EMS197071-supplement-Supplementary_Materials_Combined.pdf]

Science
AAAS

Supplementary Materials for

Mef2d potentiates type-2 immune responses and allergic lung inflammation

Aydan C. H. Szeto, Paula A. Clark, Ana C. F. Ferreira, Morgan Heycock, Emma L. Griffiths, Eric Jou, Jonathan Mannion, Shi-Lu Luan, Sophie Storrar, Martin D. Knolle, Patrycja Kozik, Helen E. Jolin, Padraic G. Fallon and Andrew N. J. McKenzie

Corresponding author: anm@mrc-lmb.cam.ac.uk, aszeto@mrc-lmb.cam.ac.uk, and pclark@mrc-lmb.cam.ac.uk.

The PDF file includes:

Materials and Methods
Figs. S1 to S24
Tables S1 and S2
References (84-85)

Other Supplementary Materials for this manuscript include the following:

Data File S1
MDAR Reproducibility Checklist

Materials and Methods

Generation of *Icos*-Cre mouse

Since the Ensembl data base entry for murine *Icos* is annotated with two potential transcripts which have different protein C termini encoded by alternatively spliced final exons we attempted to target the locus such that expression of either transcript would result in the expression of the Dre activated Cre transgene and in a way to preserve expression of the 200 amino acid protein which is predominantly described in the literature *(84)* and has an NCBI protein reference entry (NP_059508.2). To this end we targeted our construct to the end of the region of the fourth exon which is shared by both transcripts which would result in the 200 amino acid version losing its final four amino acids. To address this, we therefore included at the 5' end of our cassette a short sequence to replace these amino acids but with the valine at position 198 replaced by isoleucine to avoid the potential of creating a cryptic splice donor site (GT). This sequence was followed by a single T such that the subsequent Rox-stop-Rox cassette would retain the necessary open reading frame for the T2A-Cre sequence to be expressed after Dre mediated recombination. Consequently, the unrecombined version of this targeted *Icos* allele would therefore express an ICOS protein tagged by 13 amino acids encoded by the Rox sequence whilst the recombined version would carry an extra 28 amino acids at its C terminus consisting of the Rox sequence and the T2A sequence. Despite these alterations we observed that ICOS protein expression levels were reduced by targeting of the allele. However, we had observed this previously in our ICOST mice without evidence of T cell-intrinsic defects following IL-33 administration or *N. brasiliensis* infection *(23)*. Downstream of the Rox-stop-Rox-T2A-Cre cassette we included a FRT site-flanked neomycin resistance cassette to facilitate selection of targeted ES cell clones and to allow Flp-mediated excision of this cassette from the resultant mice. ES cell targeting was achieved by transfection with the targeting construct described above with 1 kb arms of homology to the target sequence (fig. S8A) and expression constructs for wildtype Cas9 and a sgRNA that recognised a sequence overlapping the site of insertion. Neomycin resistant ES cell clones were screened and verified by Southern analysis (fig. S8B).

Generation of *Il13*-Dre mouse

To generate an allele at the *Il13* locus that would express Dre under the control of the *Il13* promoter we inserted a wildtype EMCV IRES sequence *(85)* upstream of the Dre recombinase sequence into the 3' untranslated region of exon 4 of *Il13*. This cassette was flanked by Vox sites to permit its excision by Vika recombinase. Following these sequences, we included a neomycin selection cassette flanked by Frt (F5) sites to facilitate selection of targeted ES cell clones and to allow Flp-mediated excision of this cassette from the resultant mice. ES cell targeting was achieved by transfection with the targeting construct described above with 1 kb arms of homology to the target sequence (fig. S8C) and expression constructs for the D10A nickase Cas9 variant and two sgRNAs of opposing directions (PAMs outwards) that recognised sequences close to the site of insertion. Neomycin resistant ES cell clones were screened and verified by Southern analysis (fig. S8D).

Generation of *Cd28*-Vika mouse

To generate an allele at the *Cd28* locus that would express Vika recombinase under the control of the *Cd28* promoter we inserted a T2A-Vika cassette at the C terminus of the *Cd28* coding sequence within exon 4, removing the stop codon. Following this we included a Frt (F3) flanked neomycin cassette to facilitate selection of targeted ES cell clones and to allow Flp-mediated excision of this

cassette from the resultant mice. ES cell targeting was achieved by transfection with the targeting construct described above with 1 kb arms of homology to the target sequence (fig. S8E) and expression constructs for wildtype Cas9 and a sgRNA that recognised a sequence close to the site of insertion. Neomycin resistant ES cell clones were screened and verified by Southern analysis at the 3' end (fig. S8F) and the 5' insertion site was confirmed by amplifying by PCR and sequencing with the primers indicated (fig. S8E).

Fig. S1

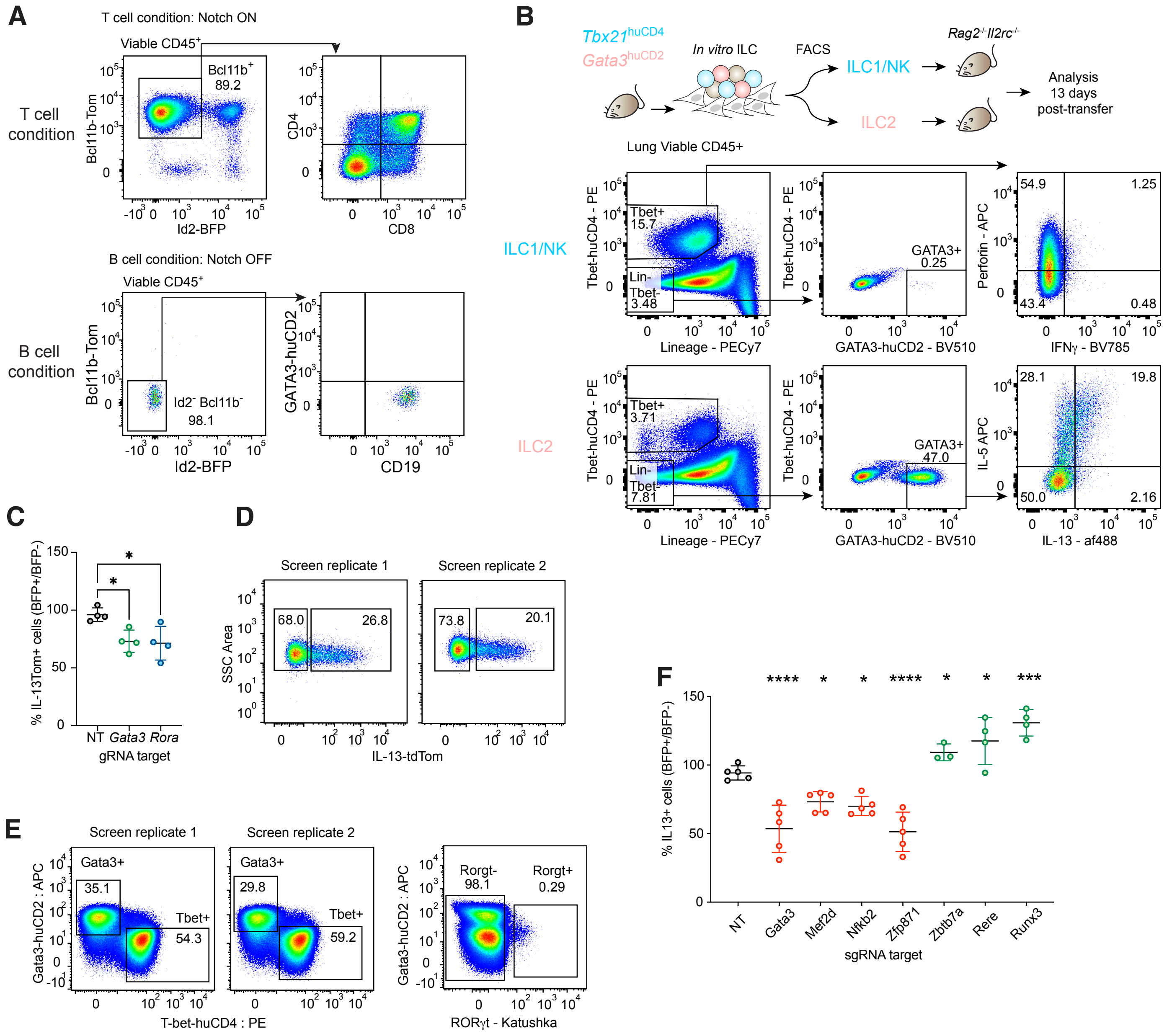

A
T cell condition: Notch ON
Viable CD45+
T cell condition
Bcl11b+ 89.2
Bcl11b-Tom
Id2-BFP
CD4
CD8
B cell condition: Notch OFF
Viable CD45+
B cell condition
Id2- Bcl11b- 98.1
Id2-BFP
GATA3-huCD2
CD19
B
Tbx21huCD4
Gata3huCD2
In vitro ILC
FACS
ILC1/NK
ILC2
Rag2-/-Il2rc-/-
Analysis 13 days post-transfer
Lung Viable CD45+
ILC1/NK
Tbet+ 15.7
Lin- Tbet- 3.48
Tbet-huCD4 - PE
Lineage - PECy7
GATA3+ 0.25
GATA3-huCD2 - BV510
54.9
1.25
43.4
0.48
Perforin - APC
IFNγ - BV785
ILC2
Tbet+ 3.71
Lin- Tbet- 7.81
GATA3+ 47.0
28.1
19.8
50.0
2.16
IL-5 APC
IL-13 - af488
C
% IL-13Tom+ cells (BFP+/BFP-)
NT Gata3 Rora
gRNA target
D
Screen replicate 1
Screen replicate 2
68.0
26.8
73.8
20.1
SSC Area
IL-13-tdTom
E
Screen replicate 1
Screen replicate 2
Gata3+
35.1
Tbet+
54.3
Gata3+
29.8
Tbet+
59.2
Gata3-huCD2 : APC
T-bet-huCD4 : PE
Rorgt- 98.1
Rorgt+ 0.29
RORγt - Katushka
F
% IL 13+ cells (BFP+/BFP-)
NT
Gata3
Mef2d
Nfkb2
Zfp871
Zbtb7a
Rere
Runx3
sgRNA target

fig. S1: Optimisation of the ILC culture for CRISPR-Cas9 screening

(A) Flow cytometric analysis of progeny cells following the culture of sorted CLPs purified from 5x polychromILC mice in T or B cell conditions. The expanded CLPs retained T cell, B cell and ILC potential. Data are representative of 2 independent experiments with n=3 biologically independent samples in each experiment.

(B) Flow cytometric analysis of progeny cells following the transfer of sorted ILC2s or ILC1/NK cells, following ILC culture, into lymphopenic mice. Progeny cells retained their discrete ILC characteristics following transfer. Data are representative of 2 independent experiments with n=3 biologically independent samples in each experiment.

(C) Validation of the CRISPR-Cas9 screening protocol using sgRNAs targeting known regulators of ILC2 development. The percentage of IL-13Tom-expressing cells from CRISPR-KO cells is normalised to the untransduced (BFP-) cells in each well. sgRNAs targeting *Gata3* or *Rora* reduced the percentage of IL-13Tom-expressing ILC2s as compared to NT control. Data are representative of 2 independent experiments with n=2 biologically independent samples in each experiment, and 4 different sgRNAs targeting each gene; mean ± SD.

(D) Representative sorting strategies of two independent screens comparing sgRNA enrichment or depletion between IL-13Tom^{-} and IL-13Tom^{+} cell populations.

(E) Representative sorting strategies of two independent screens comparing sgRNA enrichment or depletion between (GATA3^{low}T-bet^{+}) and ILC2s (GATA3^{high}T-bet^{-}) cell populations. (right) RORγt+ ILC3s were excluded from sgRNA enrichment analyses due to their low abundance in the assay resulting in insufficient library representation.

(F) Flow cytometric quantification of IL-13 expression by cultured ILCs transduced with sgRNAs targeting the indicated genes. The percentage of IL-13Tom-expressing cells from CRISPR-KO cells is normalised to the untransduced (BFP-) cells in each well. Data are representative of 2 independent experiments with n=2 biologically independent samples in each experiment and at least 3 different sgRNAs targeting each gene; mean ± SD.

Significance in (C) & (F) was determined using one-way ANOVA with Dunnett's post-hoc test (compared to NT sgRNA). *P<0.05; ***P<0.001; ****P<0.0001. Individual data point denotes different sgRNAs targeting each gene.

Fig. S2

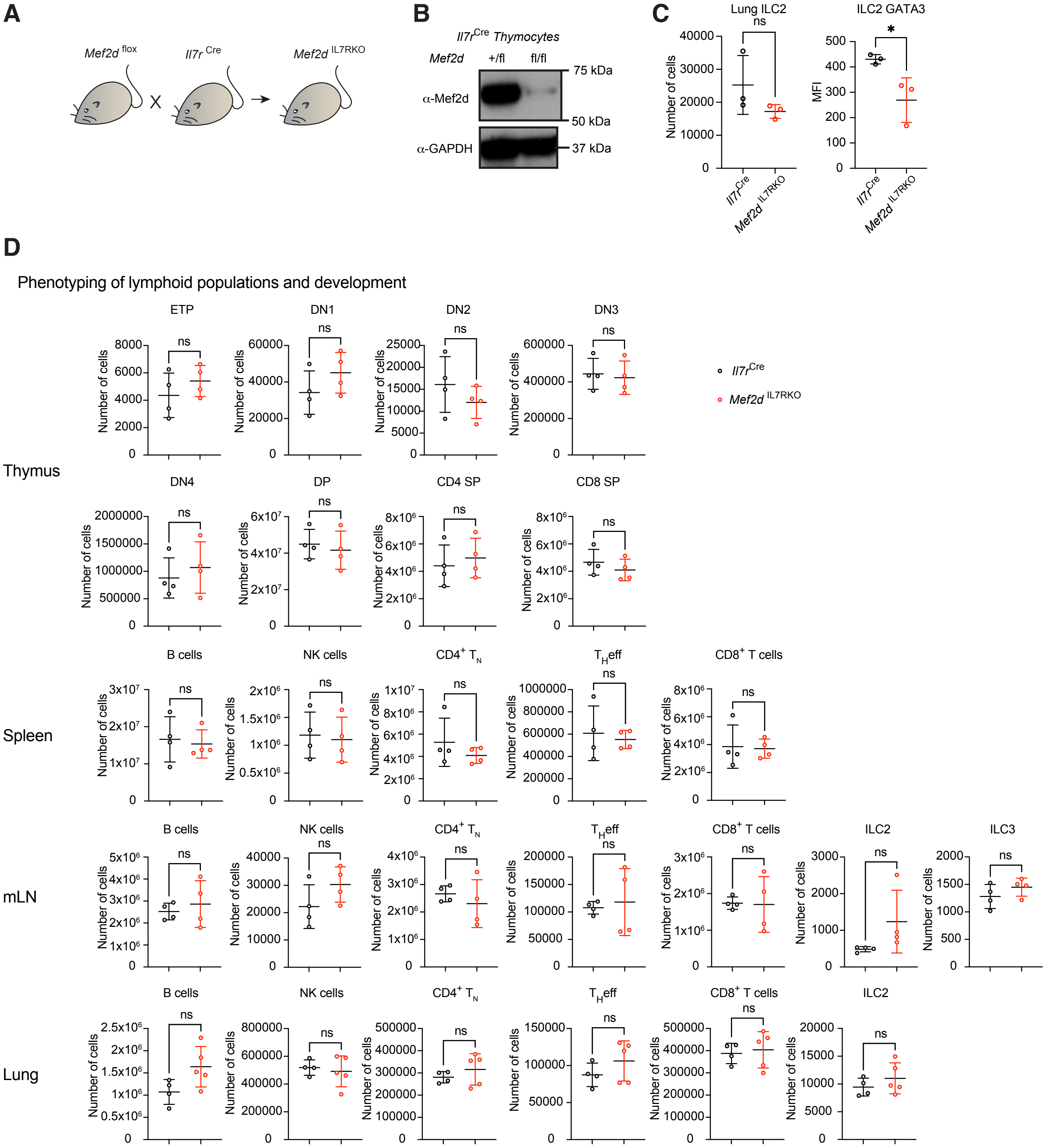
A
Mef2d flox
Il7r Cre
Mef2d IL7RKO
X
B
Il7rCre Thymocytes
Mef2d
+/fl
fl/fl
α-Mef2d
α-GAPDH
75 kDa
50 kDa
37 kDa
C
Lung ILC2
ILC2 GATA3
ns
*
Number of cells
MFI
Il7rCre
Mef2d IL7RKO
D
Phenotyping of lymphoid populations and development
Thymus
ETP
DN1
DN2
DN3
DN4
DP
CD4 SP
CD8 SP
Spleen
B cells
NK cells
CD4+ TN
THeff
CD8+ T cells
mLN
ILC2
ILC3
Lung
ns
Number of cells

fig. S2: Generation of conditional Mef2d-deficient mice and phenotyping of their homeostatic lymphoid populations.

(A) Schematic of the generation of *Mef2d*^{IL7RKO} mice by intercrossing *Mef2d*^{flox} mice with *Il7r*^{Cre} mice.

(B) Western blotting detection of Mef2d and GAPDH (loading control) protein in thymocyte protein lysates derived from *Il7r*^{Cre} and *Mef2d*^{IL7RKO} mice.

(C) Quantification of lung ILC2s and their GATA3 MFI at homeostasis. Data are representative of 2 independent experiments with n=3 biologically independent samples in each experiment; mean ± SD.

(D) Quantification of thymic progenitors and peripheral lymphoid populations (spleen, mesenteric lymph node and lung) in *Il7r*^{Cre} or *Mef2d*^{IL7RKO} mice. Representative gating strategies are shown in fig. S3. Data are representative of 2 independent experiments with n=4 biologically independent samples in each experiment; mean ± SD.

Significance in (C) & (D) was determined using unpaired two-sided t-test; ns, not significant; *$P<0.05$; individual data point denotes biological replicates.

Fig. S3

A Representative gating strategy for the phenotyping of homeostatic lymphoid populations

Thymus

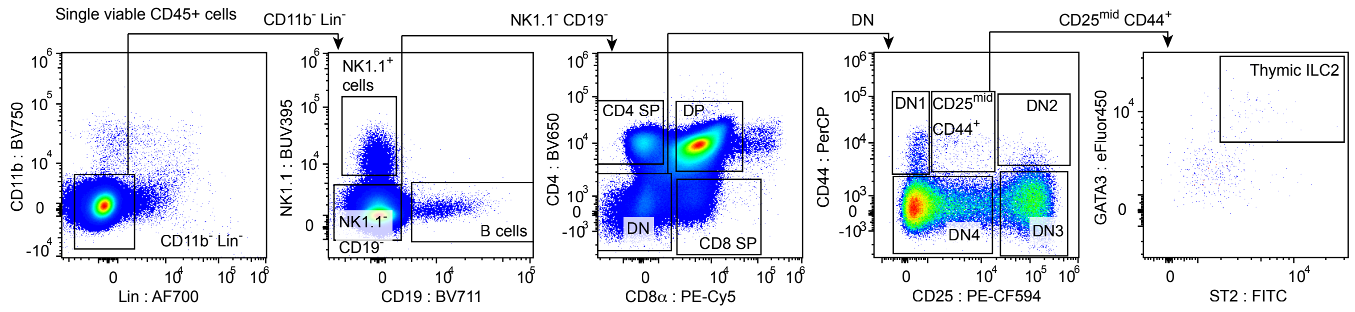

B

Lung, Adipose, siLP, cLP, mLN, spleen, bone marrow, liver

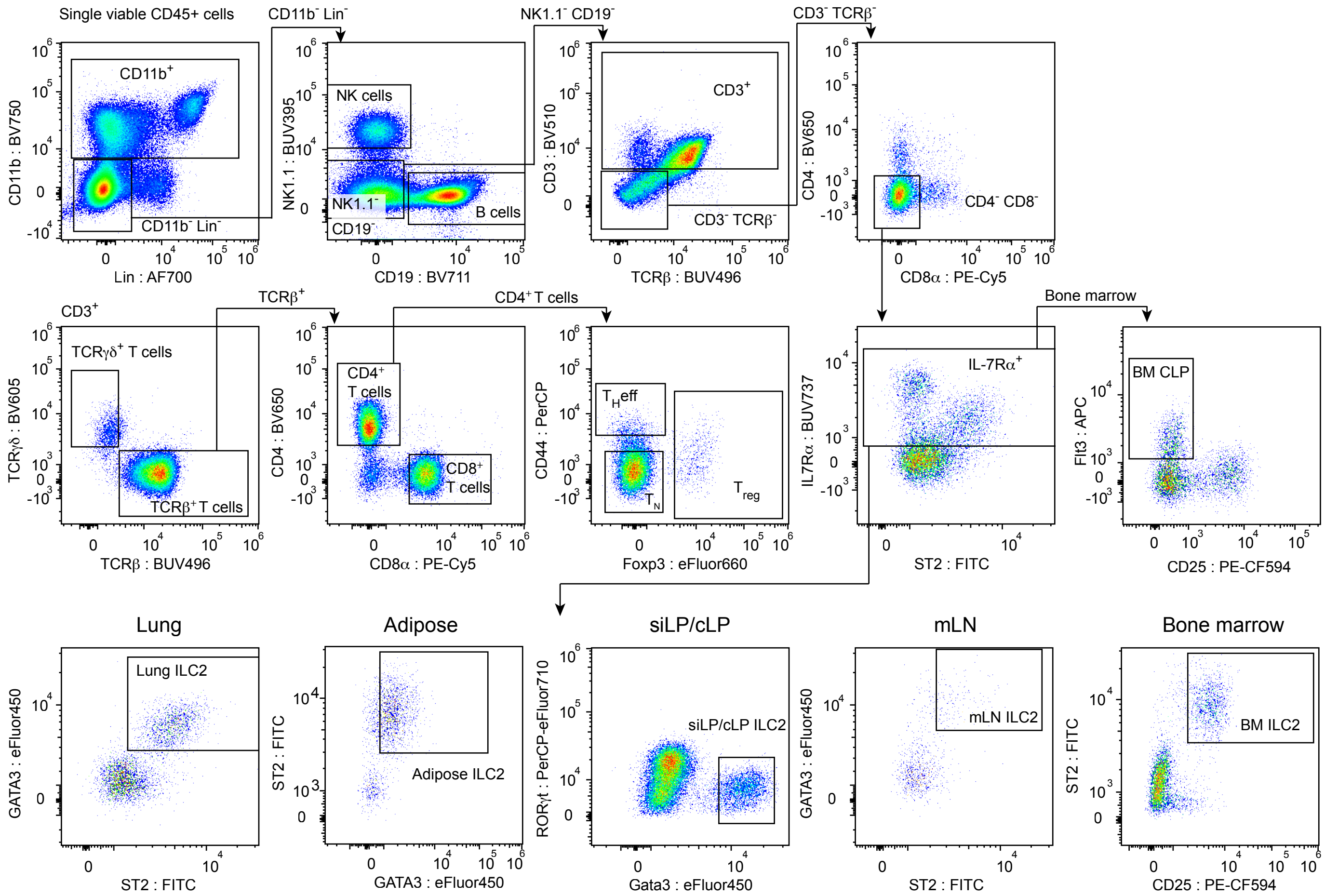

fig. S3: Gating strategies for lymphoid populations at homeostasis

(A) Representative gating strategies for the cell populations analysed from the thymus. Lineage staining: CD11c, FcεR1, GR-1, Ter119.

(B) Representative gating strategies for cell populations analysed from the lung, adipose tissue, small intestinal lamina propria (siLP), colonic lamina propria (cLP), mesenteric lymph node (mLN), bone marrow, liver and spleen. Lineage staining: CD11c, FcεR1, GR-1, Ter119.

Fig. S4

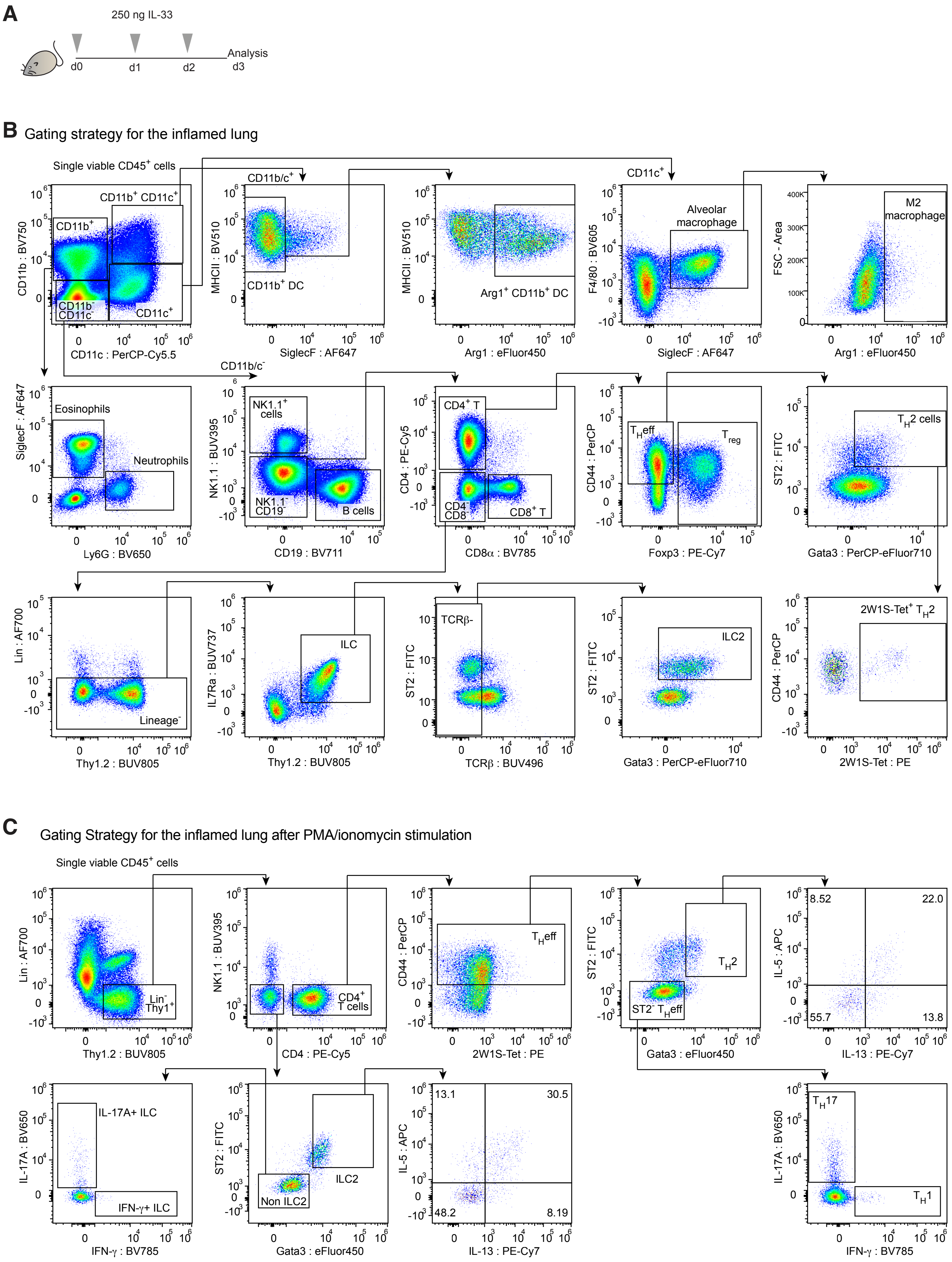
A
250 ng IL-33
d0
d1
d2
Analysis
d3
B Gating strategy for the inflamed lung
Single viable CD45+ cells
CD11b+ CD11c+
CD11b+
CD11b- CD11c-
CD11c+
CD11b : BV750
CD11c : PerCP-Cy5.5
CD11b/c+
CD11b+ DC
MHCII : BV510
SiglecF : AF647
Arg1+ CD11b+ DC
Arg1 : eFluor450
CD11c+
Alveolar macrophage
F4/80 : BV605
M2 macrophage
FSC - Area
CD11b/c-
Eosinophils
Neutrophils
SiglecF : AF647
Ly6G : BV650
NK1.1+ cells
NK1.1- CD19-
B cells
NK1.1 : BUV395
CD19 : BV711
CD4+ T
CD4- CD8-
CD8+ T
CD4 : PE-Cy5
CD8α : BV785
THeff
Treg
CD44 : PerCP
Foxp3 : PE-Cy7
TH2 cells
ST2 : FITC
Gata3 : PerCP-eFluor710
Lineage-
Lin : AF700
Thy1.2 : BUV805
ILC
IL7Ra : BUV737
TCRβ-
TCRβ : BUV496
ILC2
2W1S-Tet+ TH2
2W1S-Tet : PE
C Gating Strategy for the inflamed lung after PMA/ionomycin stimulation
Single viable CD45+ cells
Lin- Thy1+
CD4+ T cells
CD4 : PE-Cy5
THeff
2W1S-Tet : PE
TH2
ST2- THeff
Gata3 : eFluor450
8.52
22.0
55.7
13.8
IL-5 : APC
IL-13 : PE-Cy7
IL-17A+ ILC
IFN-γ+ ILC
IL-17A : BV650
IFN-γ : BV785
ILC2
Non ILC2
13.1
30.5
48.2
8.19
TH17
TH1

fig. S4: Gating strategy for the identification of immune populations during inflammation

(A) Schematic of the experimental induction of IL-33-mediated lung inflammation.

(B) Gating strategy to identify myeloid and lymphoid populations from lung tissue and the BAL fluid.

(C) Gating strategy to identify cytokine-expressing lymphocytes following *ex vivo* PMA/ionomycin stimulation.

Fig. S5

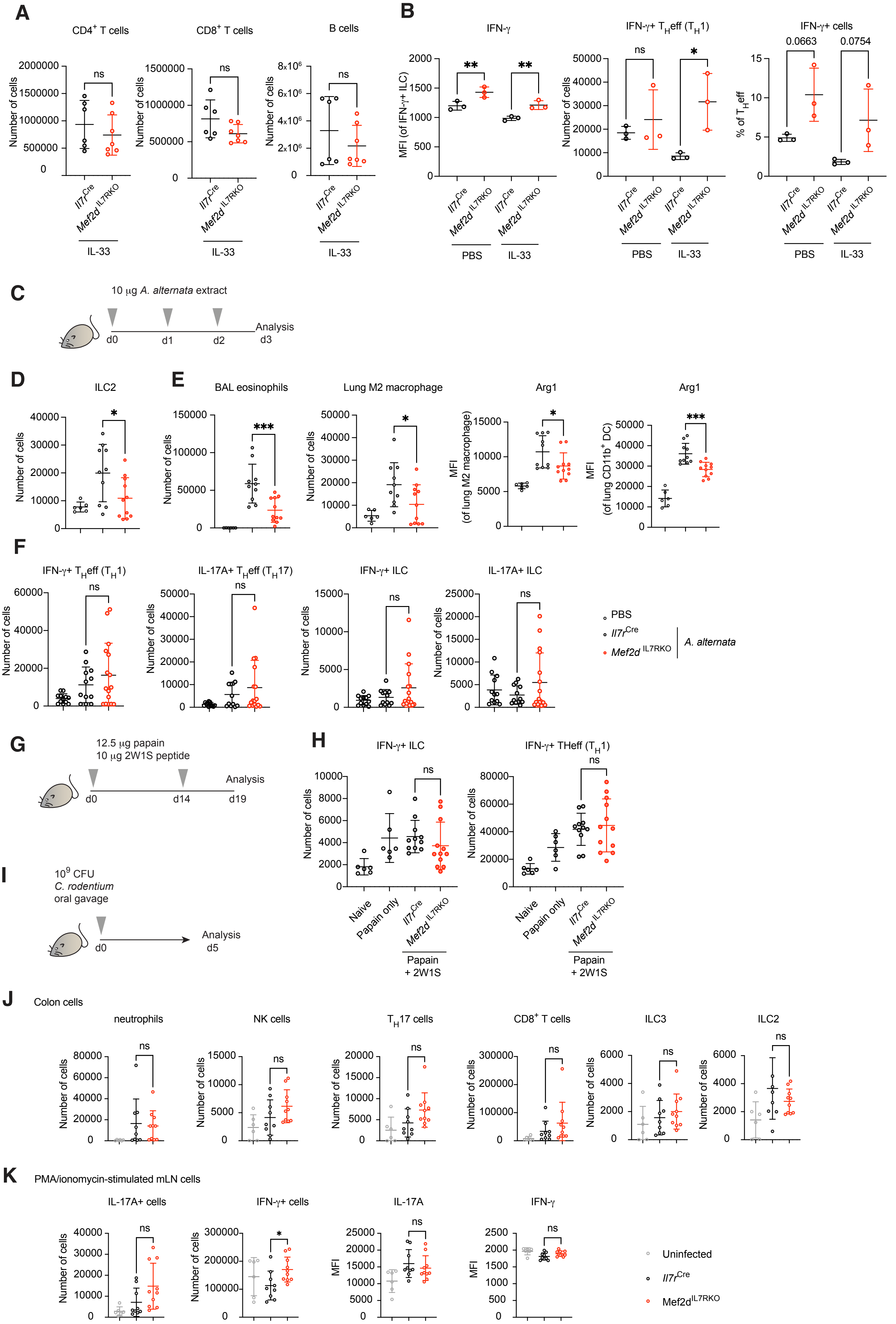
A
CD4+ T cells
CD8+ T cells
B cells
Number of cells
ns
Il7rCre
Mef2d IL7RKO
IL-33
B
IFN-γ
IFN-γ+ THeff (TH1)
IFN-γ+ cells
MFI (of IFN-γ+ ILC)
% of THeff
0.0663
0.0754
PBS
IL-33
C
10 µg A. alternata extract
d0
d1
d2
Analysis
d3
D
ILC2
E
BAL eosinophils
Lung M2 macrophage
Arg1
MFI (of lung M2 macrophage)
MFI (of lung CD11b+ DC)
F
IFN-γ+ THeff (TH1)
IL-17A+ THeff (TH17)
IFN-γ+ ILC
IL-17A+ ILC
PBS
A. alternata
G
12.5 µg papain
10 µg 2W1S peptide
d14
d19
H
IFN-γ+ ILC
IFN-γ+ THeff (TH1)
Naive
Papain only
Papain + 2W1S
I
109 CFU
C. rodentium
oral gavage
Analysis
d5
J
Colon cells
neutrophils
NK cells
TH17 cells
CD8+ T cells
ILC3
ILC2
K
PMA/ionomycin-stimulated mLN cells
IL-17A+ cells
IFN-γ+ cells
IL-17A
MFI
Uninfected
Mef2dIL7RKO

fig. S5: Pan-lymphoid Mef2d deficiency did not affect type-1 or type-17 cytokine expression

(A) Quantification of $CD4^+$ T cells, $CD8^+$ T cells and B cells in *Il7r*^{Cre} or *Mef2d*^{IL7RKO} mice treated with IL-33. Data are pooled from 2 independent experiments and represent mean ± SD; n=6-7 mice; mean ± SD.

(B) Quantification of IFN-γ-expressing innate and T_H lymphocytes from PBS or IL-33 treated *Il7r*^{Cre} and *Mef2d*^{IL7RKO} mice. Data are representative of 2 independent experiments and represent mean ± SD; n=3 mice (PBS groups) and n=3 mice (IL-33 groups); mean ± SD.

(C) Schematic of the experimental induction of *A. alternata* extract-mediated lung inflammation.

(D) Quantification of lung ILC2s from PBS or *A. alternata*-treated *Il7r*^{Cre} or *Mef2d*^{IL7RKO} mice.

(E) Quantification of BAL eosinophils, lung M2 macrophages, Arg1 MFI of lung M2 macrophages and Arg1 MFI of lung $CD11b^+$ DCs from PBS or *A. alternata*-treated *Il7r*^{Cre} or *Mef2d*^{IL7RKO} mice.

(F) Quantification of IFN-γ- or IL-17A-expressing innate and T_H lymphocytes from PBS or *A. alternata*-treated *Il7r*^{Cre} or *Mef2d*^{IL7RKO} mice.
(D) – (E) Data are pooled from 3 independent experiments and represent mean ± SD; n=6 mice in PBS group, n=10-11 mice in *A. alternata* -treated groups. (F) Cytokine expression data are pooled from 3 independent experiments and represent mean ± SD; n=12 mice in PBS group, n=12-16 mice in *A. alternata* -treated groups.

(G) Schematic of the experimental induction of papain+2W1S-mediated lung inflammation. The cysteine protease activity of papain orchestrates robust pulmonary type-2 inflammation, which in the presence of 2W1S peptide leads to the development of 2W1S-specific T_H2 cells that can be detected with 2W1S-loaded MHCII tetramers.

(H) Quantification of IFN-γ-expressing innate and T_H lymphocytes from naive, papain or papain+2W1S treated mice. Data are pooled from 2 independent experiments and represent mean ± SD; n=6 mice in naïve and papain only groups, n=11-12 mice in papain+2W1S-treated groups.

(I) Schematic of the experimental induction of type-1/17 inflammation by *Citrobacter rodentium* infection.

(J) Quantification of neutrophils, NK cells, T_H17 cells, $CD8^+$ T cells, ILC3s and ILC2s from uninfected, *Citrobacter rodentium*-infected *Il7r*^{Cre} or *Mef2d*^{IL7RKO} mice. Conditional Mef2d-deficiency did not affect the induction of these cell types.

(K) Quantification of total IFN-γ- or IL-17A-expressing cells and their MFI from uninfected, *Citrobacter rodentium*-infected *Il7r*^{Cre} or *Mef2d*^{IL7RKO} mice. Conditional Mef2d-deficiency led to

similar expression of type-1/17 cytokines, though there was a modest increase in the number of IFN-γ-expressing cells in *Mef2d*^{IL7RKO} mice.

(J) – (K) Data are pooled from 2 independent experiments and represent mean ± SD; n=6 mice (Uninfected group), n=9 mice (*Il7r*^{Cre} group), n=10 mice (*Mef2d*^{IL7RKO} group).

Significance was determined using unpaired two-sided t-test [(A)] or one-way ANOVA with Šidák's multiple comparisons test between the indicated groups [(B) - (K)]; ns, not significant; *P<0.05; **P<0.01; ***P<0.001; individual data point denotes biological replicates.

Fig. S6

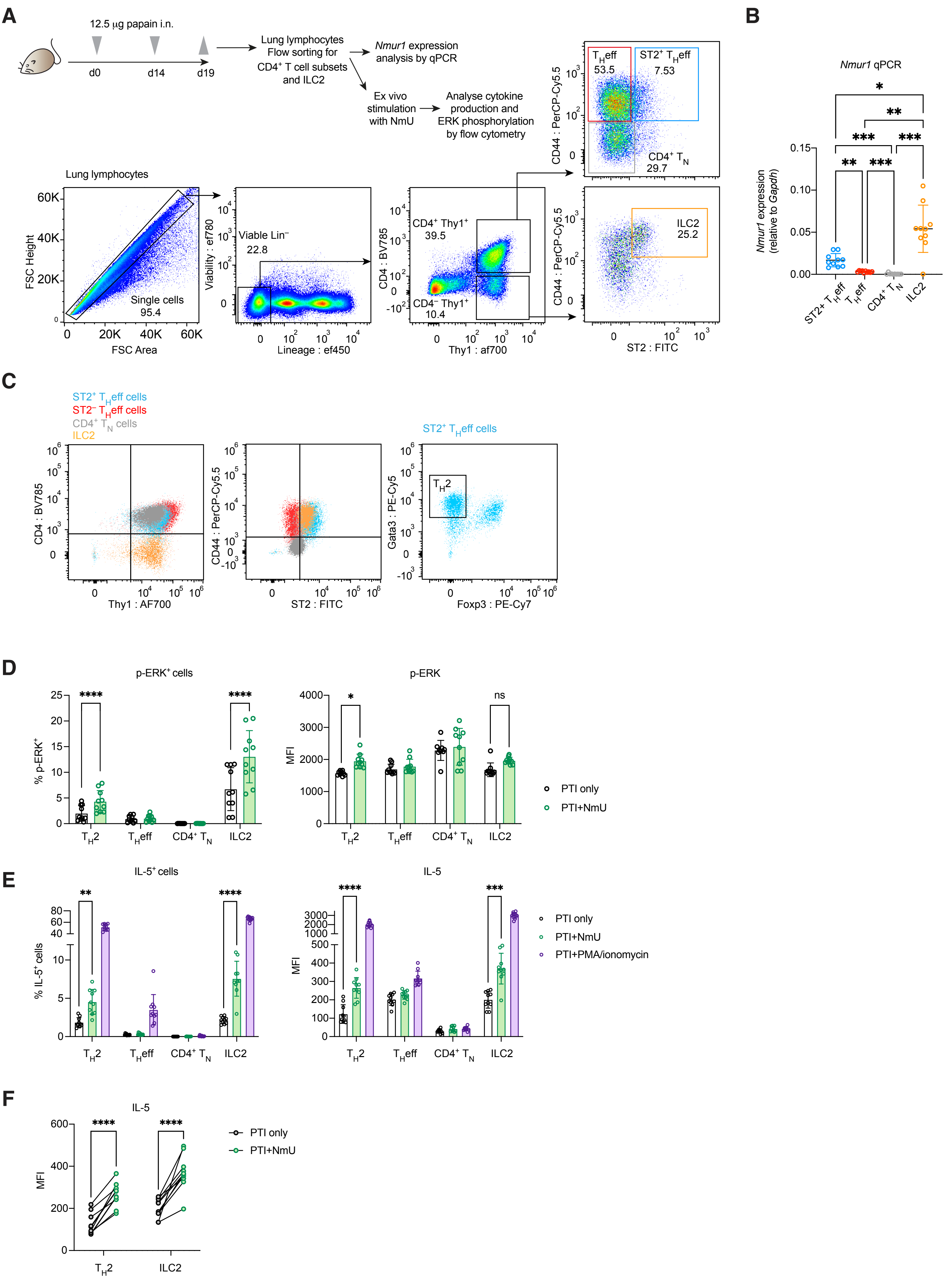
A
12.5 μg papain i.n.
d0
d14
d19
Lung lymphocytes Flow sorting for CD4+ T cell subsets and ILC2
Nmur1 expression analysis by qPCR
Ex vivo stimulation with NmU
Analyse cytokine production and ERK phosphorylation by flow cytometry
Lung lymphocytes
Single cells 95.4
FSC Height
FSC Area
Viable Lin− 22.8
Viability : ef780
Lineage : ef450
CD4+ Thy1+ 39.5
CD4− Thy1+ 10.4
CD4 : BV785
Thy1 : af700
THeff 53.5
ST2+ THeff 7.53
CD4+ TN 29.7
CD44 : PerCP-Cy5.5
ILC2 25.2
ST2 : FITC
B
Nmur1 qPCR
Nmur1 expression (relative to Gapdh)
ST2+ THeff
THeff
CD4+ TN
ILC2
C
ST2+ THeff cells
ST2− THeff cells
CD4+ TN cells
ILC2
CD4 : BV785
Thy1 : AF700
CD44 : PerCP-Cy5.5
ST2 : FITC
ST2+ THeff cells
TH2
Gata3 : PE-Cy5
Foxp3 : PE-Cy7
D
p-ERK+ cells
% p-ERK+
p-ERK
MFI
TH2
THeff
CD4+ TN
ILC2
PTI only
PTI+NmU
E
IL-5+ cells
% IL-5+ cells
IL-5
MFI
PTI only
PTI+NmU
PTI+PMA/ionomycin
F
IL-5
MFI
TH2
ILC2
PTI only
PTI+NmU

fig. S6: T_H2 cells express NMUR1 and respond to NmU stimulation

(A) Schematic of the experimental procedure to induce and flow-purify lymphocyte populations from papain-stimulated mice. Lineage staining: CD8α, CD11b, CD11c, CD19, FcεR1, GR-1, NK1.1, Ter119.

(B) Quantitative PCR analysis of the *Nmur1* transcript (normalised to *Gapdh*) from sorted cell populations as in (A). One-way ANOVA with Tukey's post-hoc test.

(C) Flow cytometric characterisation of sorted cell populations using surface and transcription factor markers.

(D) Flow cytometric analysis of phospho-ERK from the indicated sorted cell populations in response to control (protein transport inhibitor (PTI) only) or NmU stimulation. Paired two-sided t-test.

(E) Flow cytometric analysis of IL-5 expression from the indicated sorted cell populations in response to control or NmU stimulation. Paired two-sided t-test.

(F) Flow cytometric quantification of IL-5 MFI from T_H2 cells and ILC2s in response to control or NmU stimulation. Paired two-sided t-test.

(A) – (F) Data are representative of 2 independent experiments with n=10 biologically independent samples in each experiment; mean ± SD; individual data point denotes biological replicates; ns, not significant; *P<0.05; **P<0.01; ***P<0.001; ****P<0.0001.

Fig. S7

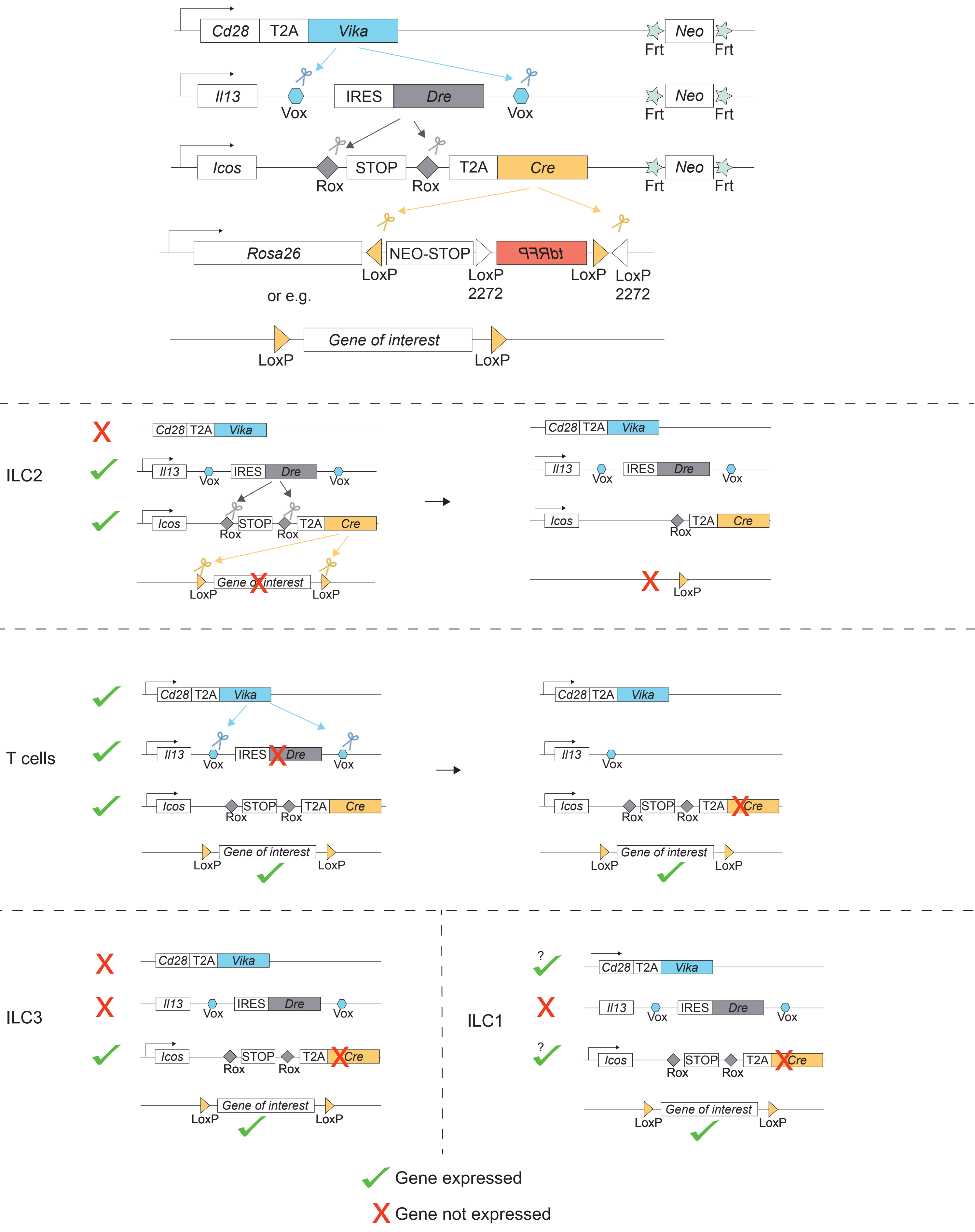

Cd28
T2A
Vika
Neo
Frt
Il13
Vox
IRES
Dre
Icos
STOP
Rox
Cre
Rosa26
NEO-STOP
tdRFP
LoxP
LoxP 2272
or e.g.
Gene of interest
ILC2
T cells
ILC3
ILC1
?
Gene expressed
Gene not expressed

fig. S7: Schematic of the Boolean SSR cascade and the outcomes in different cell subsets
In ILC2 *Cd28* is not expressed and so Vika-mediated recombination of the *Il13*-Dre locus does not occur, leading to Dre-mediated removal of the STOP cassette from the *Icos*-Cre locus, resulting in Cre expression and subsequent Cre-mediated modification of the LoxP-flanked locus. In T cells *Cd28* is expressed and so Vika-mediated recombination of the *Il13*-Dre locus occurs, leading to the deletion of the Vox-flanked Dre-allele. Consequently, Dre-mediated removal of the STOP cassette from the *Icos-Cre* locus cannot occur and Cre is not expressed, and there is no Cre-mediated modification of the LoxP-flanked locus. In ILC3 and ILC1 there is no expression of the *Il13*-Dre locus. Consequently, Dre-mediated removal of the STOP cassette from the *Icos*-Cre locus cannot occur and Cre is not expressed, and there is no Cre-mediated modification of the LoxP-flanked locus.

Fig. S8

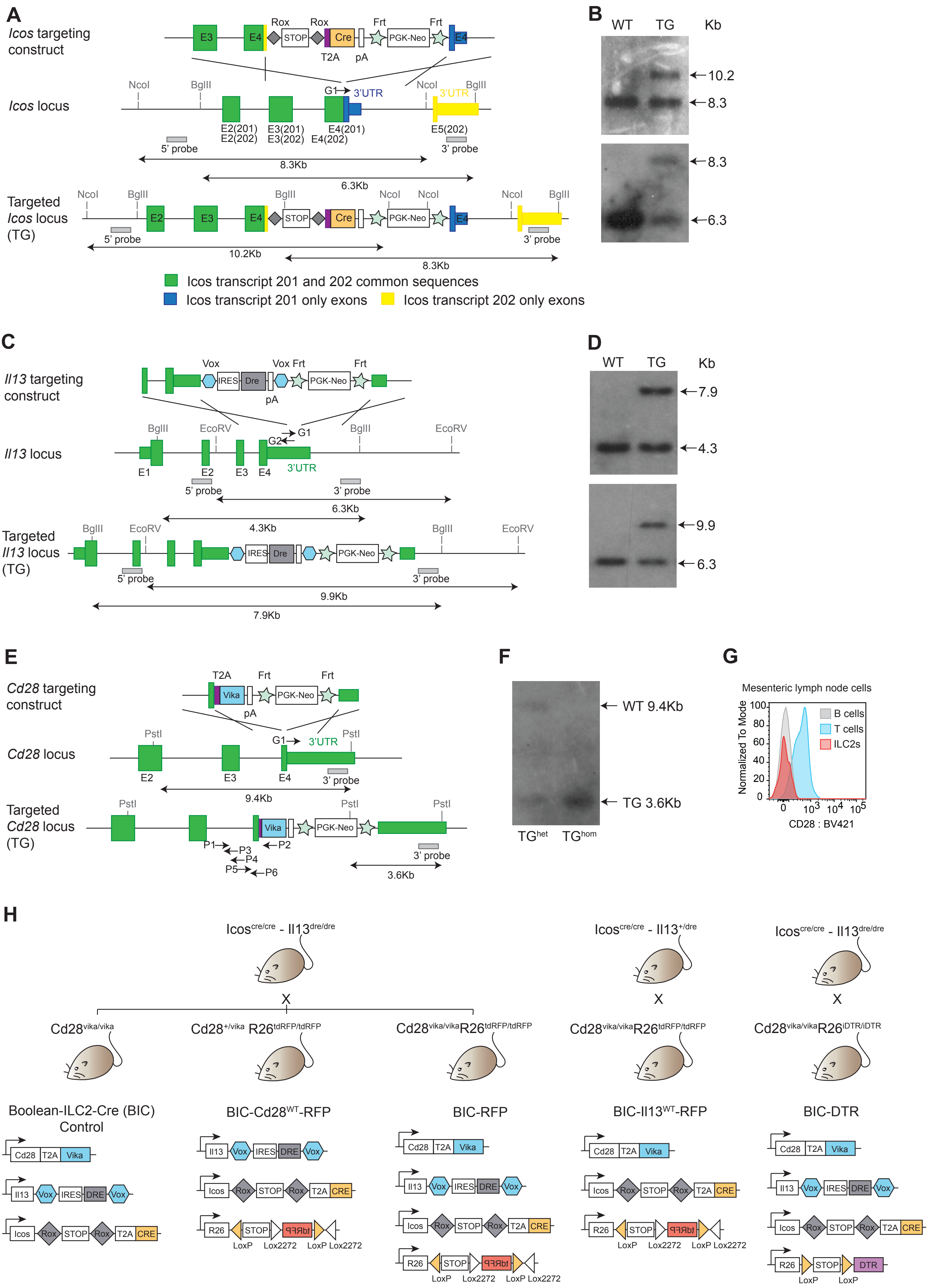

A
Icos targeting construct
Icos locus
Targeted Icos locus (TG)
E3
E4
Rox
STOP
Cre
Frt
PGK-Neo
T2A
pA
NcoI
BglII
G1
3'UTR
E2(201)
E2(202)
E3(201)
E3(202)
E4(201)
E4(202)
E5(202)
5' probe
3' probe
8.3Kb
6.3Kb
10.2Kb
Icos transcript 201 and 202 common sequences
Icos transcript 201 only exons
Icos transcript 202 only exons
B
WT
TG
Kb
10.2
8.3
6.3
C
Il13 targeting construct
Il13 locus
Targeted Il13 locus (TG)
Vox
IRES
Dre
BglII
EcoRV
G1
G2
E1
E2
E3
E4
4.3Kb
9.9Kb
7.9Kb
D
7.9
4.3
9.9
E
Cd28 targeting construct
Cd28 locus
Targeted Cd28 locus (TG)
Vika
PstI
P1
P2
P3
P4
P5
P6
9.4Kb
3.6Kb
F
WT 9.4Kb
TG 3.6Kb
TGhet
TGhom
G
Mesenteric lymph node cells
Normalized To Mode
B cells
T cells
ILC2s
CD28 : BV421
H
Icoscre/cre - Il13dre/dre
Icoscre/cre - Il13+/dre
X
Cd28vika/vika
Cd28+/vika R26tdRFP/tdRFP
Cd28vika/vikaR26tdRFP/tdRFP
Cd28vika/vikaR26iDTR/iDTR
Boolean-ILC2-Cre (BIC) Control
BIC-Cd28WT-RFP
BIC-RFP
BIC-Il13WT-RFP
BIC-DTR
Cd28
Il13
Icos
R26
DRE
CRE
tdRFP
LoxP
Lox2272
DTR

fig. S8: Targeting and breeding strategies utilised to generate Boolean ILC2 Cre mice

(A) Schematic representation of the targeting strategy used to introduce the Rox-stop-Rox-Cre cassette into the *Icos* locus. G1 indicates the location of the CRISPR guide RNA recognition site used to mediate homology directed repair to introduce the cassette. Location of restriction enzyme sites and probes used for the Southern blot verification shown in (B) is indicated on both the wild type and targeted loci along with expected fragment sizes.

(B) Southern blot analysis of the ESC clone used to generate the *Icos*-Cre strain digested with either NcoI and probed with the 5' probe or digested with BglII and probed with the 3' probe. A wild type sample (WT) is shown alongside the targeted clone (TG) for comparison.

(C) Schematic representation of the targeting strategy used to introduce the Dre cassette into the *Il13* locus. G1 and G2 indicate the location of the CRISPR guide RNA recognition sites used to mediate homology directed repair to introduce the cassette. Location of restriction enzyme sites and probes used for the Southern blot verification of targeting shown in (D) is indicated on both the wild type and targeted loci along with expected fragment sizes.

(D) Southern blot analysis of the embryonic stem cell (ESC) clone used to generate the *Il13*-Dre strain digested with either BglII and probed with the 5' probe or digested with EcoRV and probed with the 3' probe. A wild type sample (WT) is shown alongside the targeted clone (TG) for comparison.

(E) Schematic representation of the targeting strategy used to introduce the Vika cassette into the *Cd28* locus. G1 indicates the location of the CRISPR guide RNA recognition site used to mediate homology directed repair to introduce the cassette. P1 and P2 indicate the locations of the primers used to amplify across from upstream of the 5' arm of homology to within the Vika cassette and P3, P4, P5 and P6 indicate the locations of the primers used to confirm correct insertion by sequencing of the amplification product. Location of restriction enzyme sites and the probe used for the Southern blot verification of targeting shown in (F) is indicated on both the wild type and targeted loci along with expected fragment sizes.

(F) Southern blot analysis of the ESC clone used to generate the *Cd28*-Vika strain digested with PstI and probed with the 3' probe. A heterozygously targeted clone (TG^{het}) is shown alongside the homozygously targeted clone that produced the strain (TG^{hom}) for comparison.

(G) Flow cytometric analysis of CD28 expression by ILC2s (red) from the mesenteric lymph node compared to a negative population which is predominantly B cells (grey) and a positive population which is T cells (blue). ILC2s are defined as $CD45^+$, $lineage^-$ (lineage: CD4, CD5, CD8, Ly6G/Ly6C, FceR1, CD11b, CD11c, Ter119, CD19), $IL7R\alpha^+$, $ICOS^+$, $KLRG1^+$, $CD25^+$. T cells are defined as $CD45^+$, $lineage^+$, $CD3^+$ and the CD28 negative population, which is predominantly B cells is defined as $CD45^+$, $lineage^+$, $CD3^-$.

(H) Schematic representation of the breeding strategies used to generate BIC control, BIC-RFP, BIC-*Cd28*^{WT}-RFP, BIC-*Il13*^{WT}-RFP and BIC-DTR mouse strains and the alleles that each strain carries.

Fig. S9

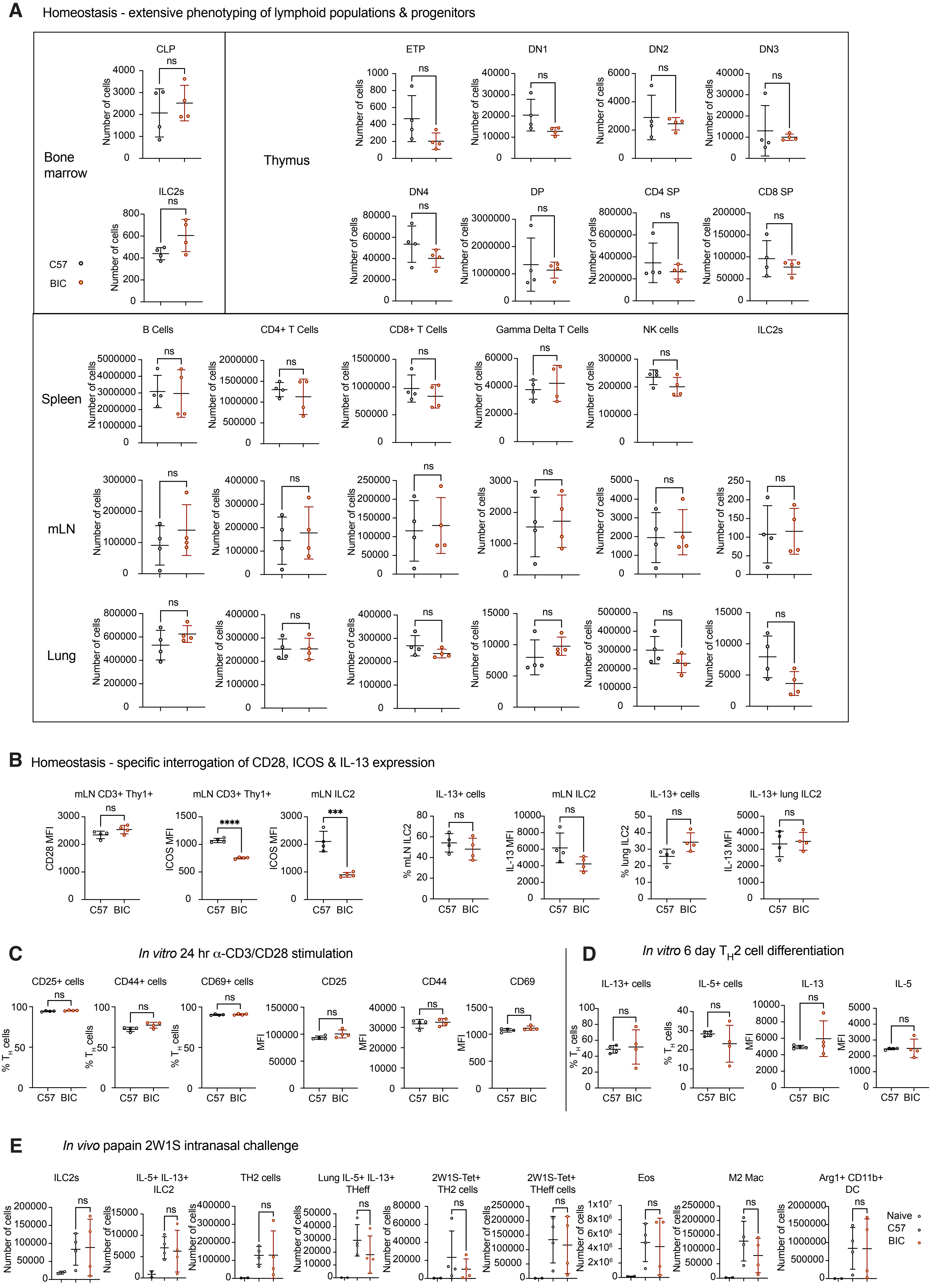
A
Homeostasis - extensive phenotyping of lymphoid populations & progenitors
Bone marrow
CLP
ILC2s
C57
BIC
Thymus
ETP
DN1
DN2
DN3
DN4
DP
CD4 SP
CD8 SP
B Cells
CD4+ T Cells
CD8+ T Cells
Gamma Delta T Cells
NK cells
ILC2s
Spleen
mLN
Lung
Number of cells
ns
B
Homeostasis - specific interrogation of CD28, ICOS & IL-13 expression
mLN CD3+ Thy1+
mLN CD3+ Thy1+
mLN ILC2
IL-13+ cells
mLN ILC2
IL-13+ cells
IL-13+ lung ILC2
CD28 MFI
ICOS MFI
% mLN ILC2
IL-13 MFI
% lung ILC2
****
***
C
In vitro 24 hr α-CD3/CD28 stimulation
CD25+ cells
CD44+ cells
CD69+ cells
CD25
CD44
CD69
MFI
D
In vitro 6 day TH2 cell differentiation
IL-13+ cells
IL-5+ cells
IL-13
IL-5
E
In vivo papain 2W1S intranasal challenge
ILC2s
IL-5+ IL-13+ ILC2
TH2 cells
Lung IL-5+ IL-13+ THeff
2W1S-Tet+ TH2 cells
2W1S-Tet+ THeff cells
Eos
M2 Mac
Arg1+ CD11b+ DC
Naive
C57
BIC

fig. S9: Phenotyping of BIC mice.

(A) Quantification of bone marrow progenitors, thymic progenitors and peripheral lymphoid populations (spleen, mesenteric lymph node and lung) in C57 or BIC mice. Data are representative of 2 independent experiments with n=4 biologically independent samples in each experiment; mean ± SD.

(B) Quantification of ICOS and CD28 expression by T cells, and ICOS and IL-13 expression by ILC2s in C57 or BIC mice. The modest reduction in ICOS expression is further noted in the Supplementary Text. Data are representative of 2 independent experiments with n=4 biologically independent samples in each experiment; mean ± SD.

(C) Quantification of CD25, CD44 and CD69 expression by T_H cells from C57 or BIC mice stimulated with α-CD3/CD28 for 24 hr. Data are representative of 2 independent experiments with n=4 biologically independent samples in each experiment; mean ± SD.

(D) Quantification of IL-5 and IL-13 expression by T_H cells from C57 or BIC mice cultured in T_H2-polarising condition for 6 days. Data are representative of 2 independent experiments with n=4 biologically independent samples in each experiment; mean ± SD.

(E) Quantification of type-2 immune response, including ILC2, IL-5$^+$ IL-13$^+$ ILC2, total T_H2 cells, IL-5$^+$ IL-13$^+$ $T_H$eff cells, 2W1S-Tetramer$^+$ T_H2 cells, total 2W1S-Tetramer$^+$ $T_H$eff cells, eosinophils, M2 macrophages and Arg1$^+$ CD11b$^+$ DC, from C57 or BIC mice challenged with papain+2W1S. Data shown from 1 experiment with n=4 biologically independent samples in the papain+2W1S-treated groups and 2 mice in the naïve group; mean ± SD.

Significance was determined using unpaired two-sided t-test [(A) – (D)] or one-way ANOVA with Šidák's multiple comparisons test between the indicated groups [(E)]; ns, not significant; ***P<0.001; ****P<0.0001; individual data point denotes biological replicates.

Fig. S10

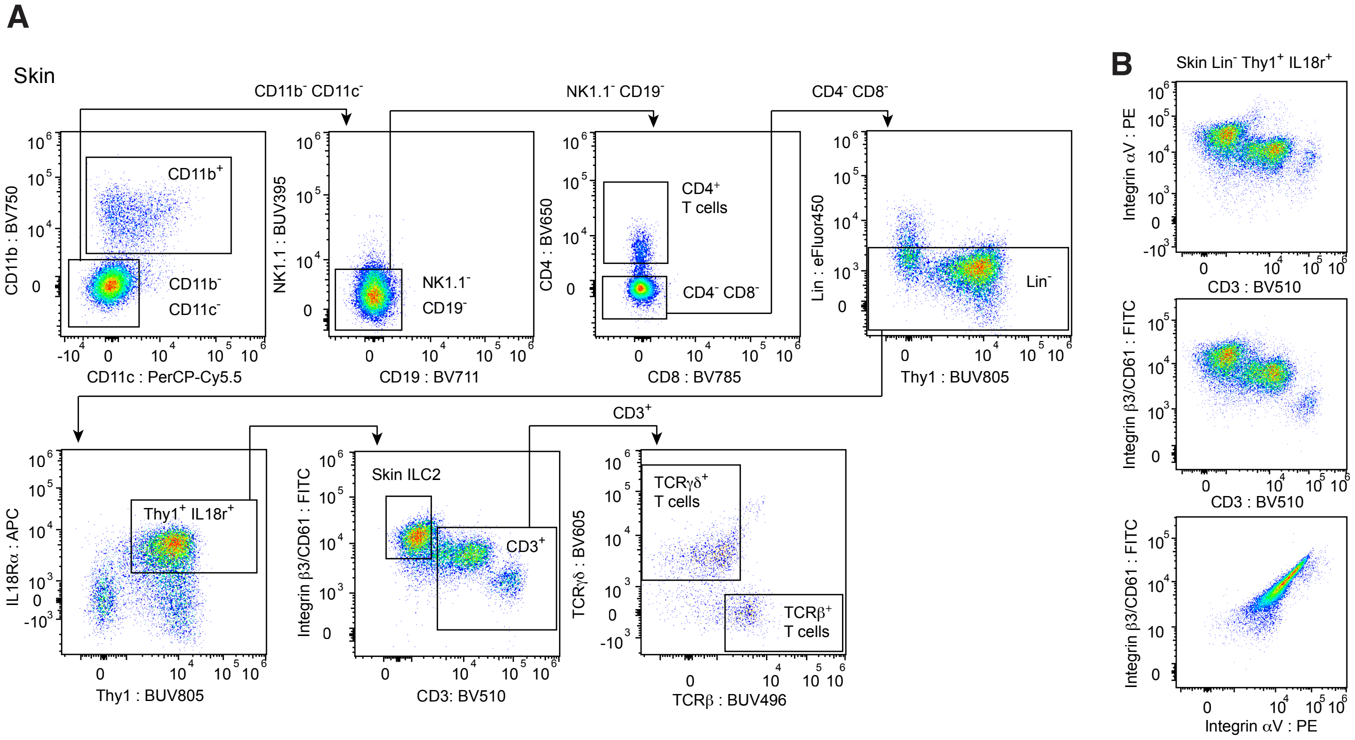
A
Skin
CD11b- CD11c-
NK1.1- CD19-
CD4- CD8-
CD11b+
CD11b- CD11c-
NK1.1- CD19-
CD4+ T cells
CD4- CD8-
Lin-
CD11b : BV750
CD11c : PerCP-Cy5.5
NK1.1 : BUV395
CD19 : BV711
CD4 : BV650
CD8 : BV785
Lin : eFluor450
Thy1 : BUV805
CD3+
Thy1+ IL18r+
Skin ILC2
CD3+
TCRγδ+ T cells
TCRβ+ T cells
IL18Rα : APC
Thy1 : BUV805
Integrin β3/CD61 : FITC
CD3: BV510
TCRγδ : BV605
TCRβ : BUV496
B
Skin Lin- Thy1+ IL18r+
Integrin αV : PE
CD3 : BV510
Integrin β3/CD61 : FITC
CD3 : BV510
Integrin β3/CD61 : FITC
Integrin αV : PE

fig. S10: Gating strategies for skin cell populations analysed in BIC-RFP mice

(A) & (B) Flow cytometric analysis of skin ILCs (Lin^{-}Thy1^{+}IL18R^{+}) demonstrating that integrin β3 (CD61) is a valid surrogate for integrin αV (a marker of skin ILC2s) since they are obligate dimer partners and that as its fluorescent labelling signal has a better dynamic range than integrin αV it is a more useful marker of ILC2s in the skin.

Fig. S11

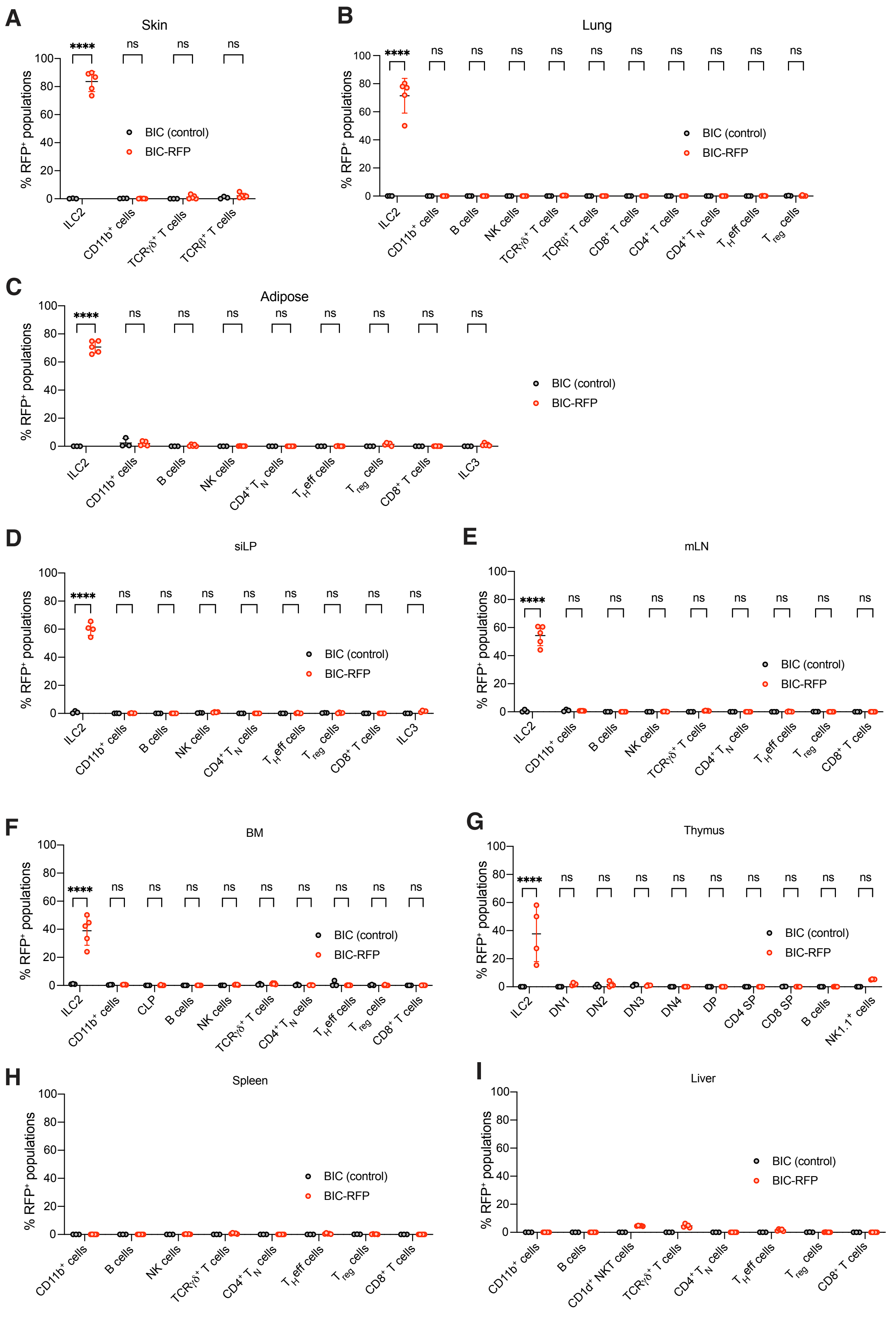
A
Skin
B
Lung
C
Adipose
D
siLP
E
mLN
F
BM
G
Thymus
H
Spleen
I
Liver
% RFP⁺ populations
BIC (control)
BIC-RFP
****
ns
ILC2
CD11b⁺ cells
B cells
NK cells
TCRγδ⁺ T cells
TCRβ⁺ T cells
CD8⁺ T cells
CD4⁺ T cells
CD4⁺ T N cells
T H eff cells
T reg cells
ILC3
CLP
DN1
DN2
DN3
DN4
DP
CD4 SP
CD8 SP
NK1.1⁺ cells
CD1d⁺ NKT cells

fig. S11: Quantification of RFP expression across multiple tissues from BIC-RFP mice

(A – I) Flow cytometric analysis and quantification of RFP expression by immune cell populations in the (A) skin, (B) lung, (C) adipose tissue, (D) small intestine lamina propria, (E) mesenteric lymph node, (F) bone marrow, (G) thymus, (H) spleen and (I) liver. Data are representative of 2 independent experiments with n=5 biologically independent samples in each experiment; mean ± SD; unpaired two-sided t-test; ns, not significant; ****P<0.0001; individual data point denotes biological replicates.

Fig. S12

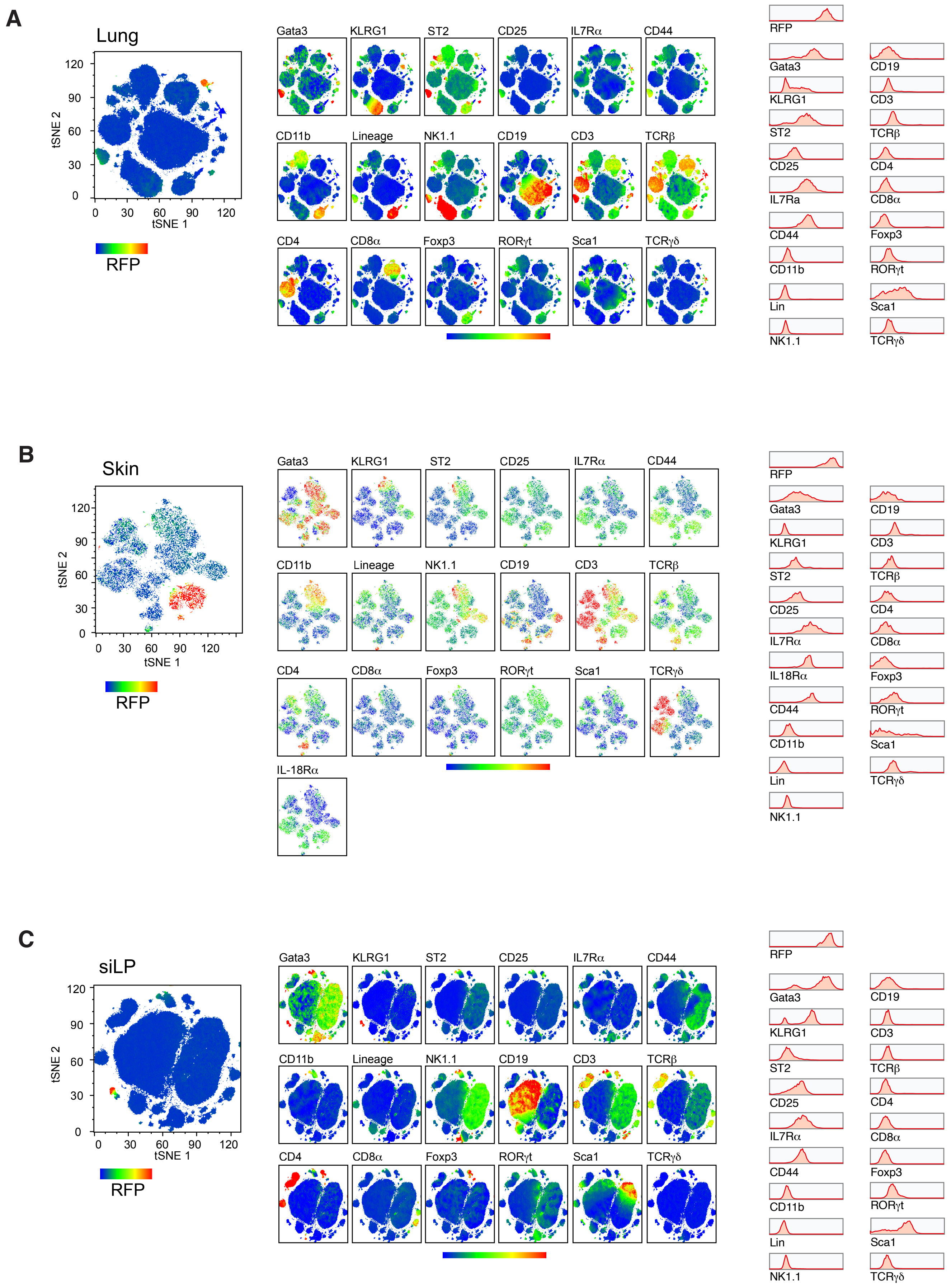
A
Lung
tSNE 2
tSNE 1
RFP
Gata3
KLRG1
ST2
CD25
IL7Rα
CD44
CD11b
Lineage
NK1.1
CD19
CD3
TCRβ
CD4
CD8α
Foxp3
RORγt
Sca1
TCRγδ
Lin
B
Skin
IL18Rα
IL-18Rα
C
siLP

fig. S12: tSNE analysis of immune cell markers and RFP expression from the lung, skin and siLP of BIC-RFP mice.

(A – C) High-dimensional spectral cytometric data from lung (A), skin (B) and small intestine lamina propria (siLP) tissue (C) from Boolean-RFP mice were analysed with the tSNE tool in FlowJo, using all recorded fluorescent parameters with the exception of viability dye and CD45 (which were used to pre-gate on viable immune cells as viability dye negative and CD45 positive events) with the default settings to generate cell clusters. Cell clusters are displayed along tSNE-1 and tSNE-2 space and the expression of markers are displayed as a heat map (middle heat map plots). To visualise markers expressed by RFP^+ cells, RFP^+ cells were pre-gated and their expression of each marker are displayed as histograms on the right. Data are representative of 2 independent experiments with n=5 biologically independent samples in each experiment.

Fig. S13

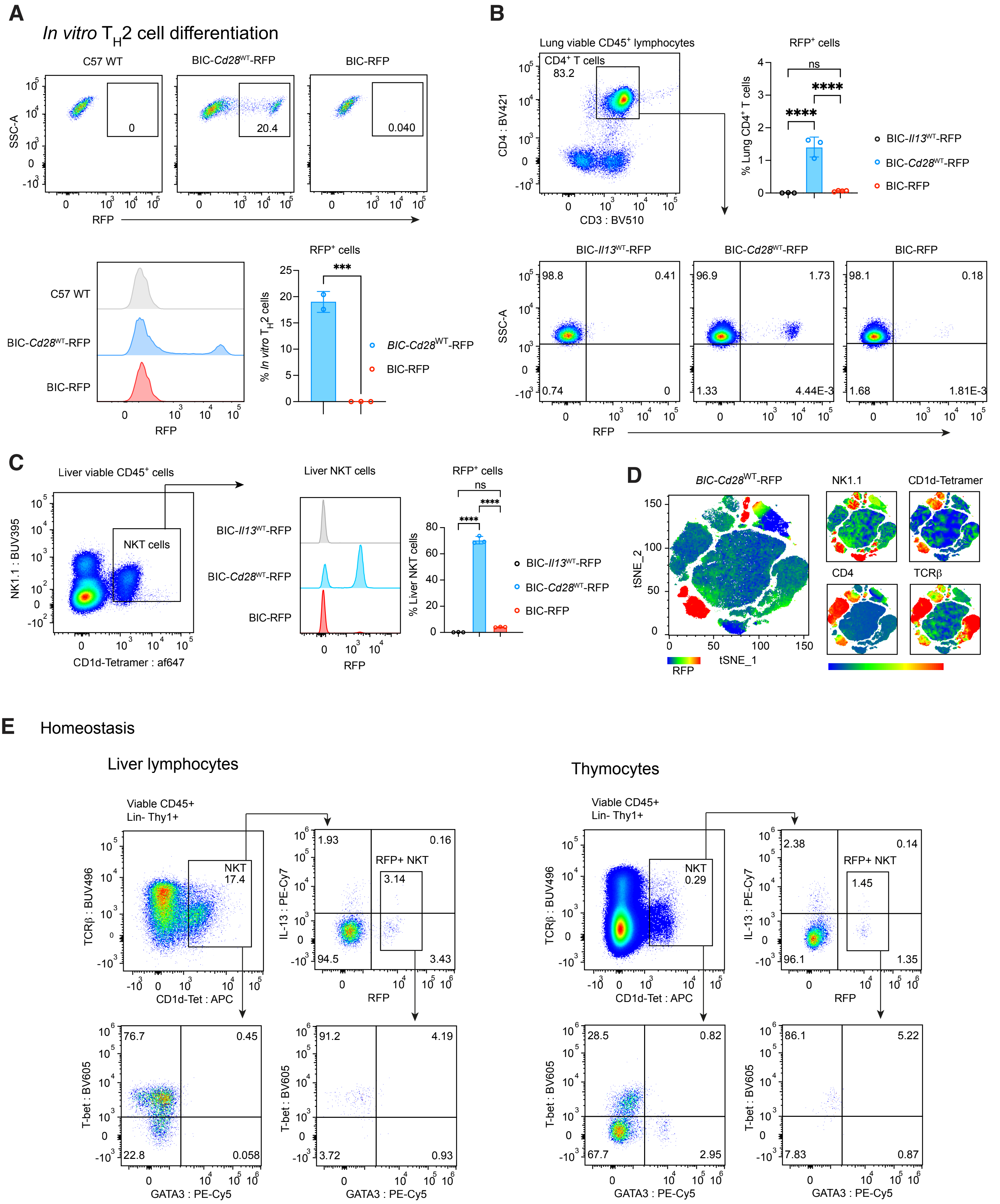
A
In vitro TH2 cell differentiation
C57 WT
BIC-Cd28WT-RFP
BIC-RFP
SSC-A
RFP
0
20.4
0.040
RFP+ cells
% In vitro TH2 cells
***
B
Lung viable CD45+ lymphocytes
CD4+ T cells
83.2
CD4 : BV421
CD3 : BV510
RFP+ cells
% Lung CD4+ T cells
ns
****
BIC-Il13WT-RFP
98.8
0.41
0.74
96.9
1.73
1.33
4.44E-3
98.1
0.18
1.68
1.81E-3
C
Liver viable CD45+ cells
NKT cells
NK1.1 : BUV395
CD1d-Tetramer : af647
Liver NKT cells
% Liver NKT cells
D
tSNE_1
tSNE_2
NK1.1
CD1d-Tetramer
CD4
TCRβ
E
Homeostasis
Liver lymphocytes
Thymocytes
Viable CD45+
Lin- Thy1+
TCRβ : BUV496
CD1d-Tet : APC
NKT
17.4
0.29
IL-13 : PE-Cy7
RFP+ NKT
1.93
0.16
3.14
94.5
3.43
2.38
0.14
1.45
96.1
1.35
T-bet : BV605
GATA3 : PE-Cy5
76.7
0.45
22.8
0.058
91.2
4.19
3.72
0.93
28.5
0.82
67.7
2.95
86.1
5.22
7.83
0.87

fig. S13: The *Cd28*-Vika 'AND NOT' allele shields T_H2 and NKT cells

(A) Flow cytometric analysis of RFP expression by T_H2 cells differentiated in vitro from naïve $CD4^+$ T cells isolated from the indicated genotypes. Data shown from 1 experiment with n=2 (BIC-*Cd28*^{WT}-RFP) and n=3 (BIC-RFP) biologically independent samples; mean ± SD.

(B) Representative flow cytometric analysis of RFP expression by lung $CD4^+$ T cells from mice with the indicated genotypes. BIC-*Il13*^{WT}-RFP n=3, BIC-*Cd28*^{WT}-RFP n=3, BIC-RFP n=4; mean ± SD.

(C) Representative flow cytometric analysis of RFP expression by liver NKT cells from mice with the indicated genotypes. The *Cd28-Vika* allele protects the vast majority of liver NKT cells from Cre activity (from 70% down to 3%). BIC-*Il13*^{WT}-RFP n=3, BIC-*Cd28*^{WT}-RFP n=3, BIC-RFP n=3; mean ± SD.

(D) A liver lymphocyte sample from BIC-*Cd28*^{WT}-RFP mice was analysed with the tSNE tool in FlowJo, using all recorded fluorescent parameters with the exception of viability dye and CD45 (which were used to pre-gate on viable immune cells as viability dye negative and CD45 positive events) with the default settings to generate cell clusters. Cell clusters are displayed along tSNE-1 and tSNE-2 space and the expression of NKT cell markers (NK1.1, CD1d-Tetramer, CD4 and TCRβ) are displayed as a heat map.

(E) Flow cytometric analysis of T-bet and GATA3 expression by total or RFP+ NKT cells from the liver or thymus of BIC-RFP mice.

Significance was determined using unpaired two-sided t-test [(A)] or one-way ANOVA with Tukey's post-hoc test [(B) & (C)]; ns, not significant; ***P<0.001; ****P<0.0001; individual data point denotes biological replicates.

Fig. S14

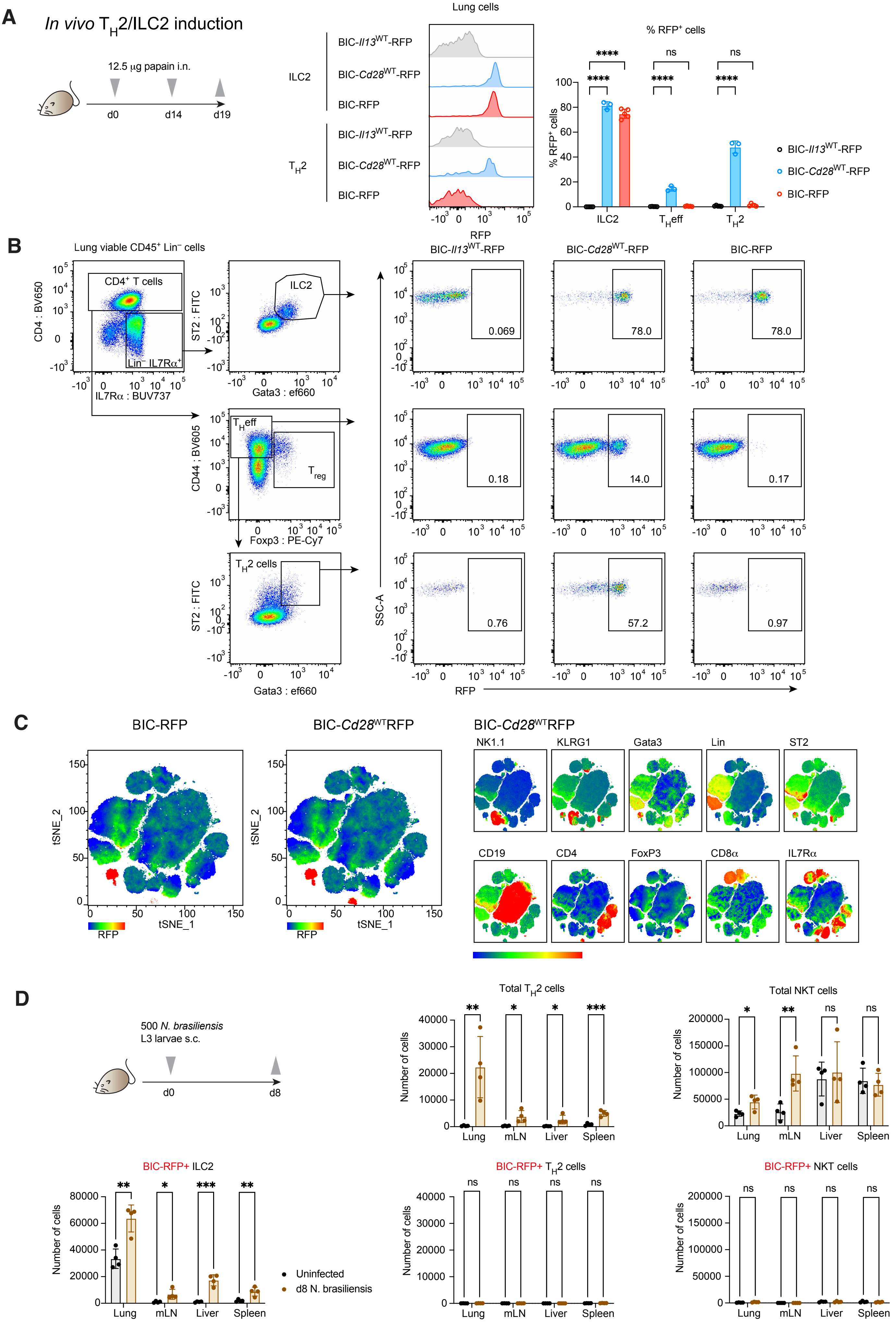
A
In vivo T_H2/ILC2 induction
12.5 µg papain i.n.
d0
d14
d19
Lung cells
ILC2
T_H2
BIC-Il13WT-RFP
BIC-Cd28WT-RFP
BIC-RFP
RFP
% RFP+ cells
% RFP+ cells
ILC2
T_Heff
T_H2
****
ns
B
Lung viable CD45+ Lin− cells
CD4+ T cells
Lin− IL7Rα+
CD4 : BV650
IL7Rα : BUV737
ST2 : FITC
Gata3 : ef660
T_Heff
T_reg
CD44 : BV605
Foxp3 : PE-Cy7
T_H2 cells
SSC-A
0.069
78.0
78.0
0.18
14.0
0.17
0.76
57.2
0.97
C
BIC-RFP
BIC-Cd28WTRFP
tSNE_1
tSNE_2
NK1.1
KLRG1
Gata3
Lin
ST2
CD19
CD4
FoxP3
CD8α
IL7Rα
D
500 N. brasiliensis
L3 larvae s.c.
d0
d8
Total T_H2 cells
Total NKT cells
Number of cells
Lung
mLN
Liver
Spleen
BIC-RFP+ ILC2
BIC-RFP+ T_H2 cells
BIC-RFP+ NKT cells
Uninfected
d8 N. brasiliensis

fig. S14: NKT and T_H2 cells are protected from Cre-labelling by the *Cd28*-Vika allele during type-2 challenge

(A) Schematic of the experimental induction of pulmonary type-2 inflammation by papain intranasal treatment and flow cytometric analysis of RFP expression by lung T_H2 cells and ILC2s from papain-treated mice with the indicated genotypes ($T_H$eff, T helper effector cells). Data are representative of 2 independent experiments with n=3 (BIC-*Il13*^{WT}-RFP and BIC-*Cd28*^{WT}-RFP) and n=5 (BIC-RFP) biologically independent samples in each experiment; mean ± SD.

(B) Gating strategy for lung T_H2 cells and ILC2s from papain-treated mice and analysis of RFP expression in mice of the indicated genotypes. Lineage staining: CD8α, CD11b, CD11c, CD19, FcεR1, GR-1, NK1.1, Ter119. Data are representative of 2 independent experiments with n=3 (BIC-*Il13*^{WT}-RFP and BIC-*Cd28*^{WT}-RFP) and n=5 (BIC-RFP) biologically independent samples in each experiment.

(C) t-SNE analysis of lung lymphocytes from papain-treated mice demonstrating the co-expression of RFP and $CD4^+$ T cell markers in BIC-*Cd28*^{WT}-RFP mice, but not BIC-RFP mice (T cell-protected).

(D) Schematic of the *N. Brasiliensis* infection model and flow cytometric analysis of RFP expression by ILC2s, T_H2 and NKT cells from the indicated tissues. Data shown for 1 experiment with n=4 mice in each group; mean ± SD.

Significance was determined using one-way ANOVA with Dunnett's post-hoc test [(A)] or multiple unpaired two-sided t-tests between the indicated samples [(D)]; ns, not significant; *P<0.05; **P<0.01; ***P<0.001; ****P<0.0001; individual data point denotes biological replicates.

Fig. S15

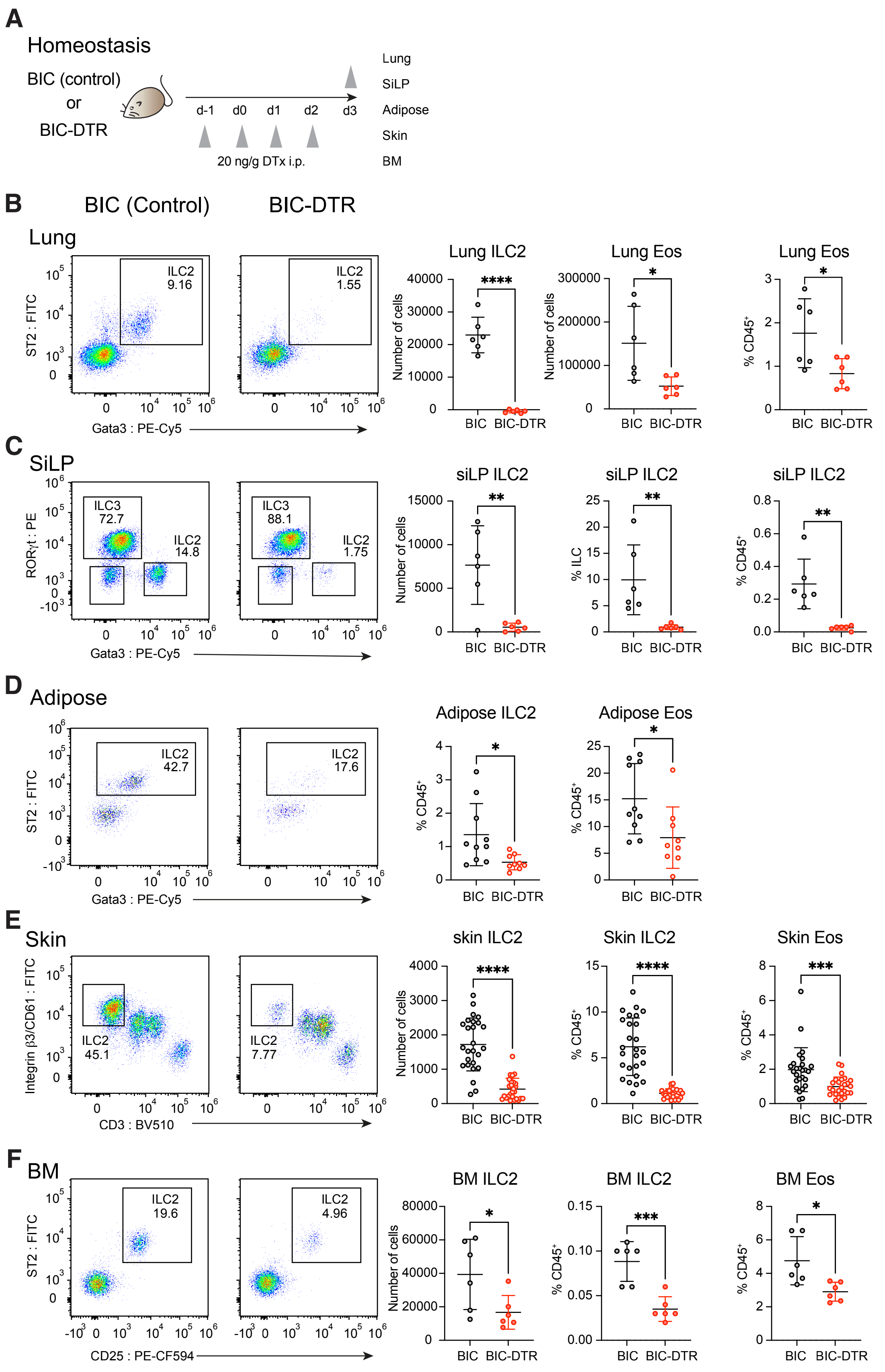
A
Homeostasis
BIC (control)
or
BIC-DTR
d-1 d0 d1 d2 d3
20 ng/g DTx i.p.
Lung
SiLP
Adipose
Skin
BM
B
BIC (Control)
BIC-DTR
Lung
ILC2 9.16
ILC2 1.55
ST2 : FITC
Gata3 : PE-Cy5
Lung ILC2
Lung Eos
Lung Eos
Number of cells
% CD45+
BIC BIC-DTR
C
SiLP
ILC3 72.7
ILC2 14.8
ILC3 88.1
ILC2 1.75
RORγt : PE
Gata3 : PE-Cy5
siLP ILC2
% ILC
D
Adipose
ILC2 42.7
ILC2 17.6
ST2 : FITC
Gata3 : PE-Cy5
Adipose ILC2
Adipose Eos
E
Skin
ILC2 45.1
ILC2 7.77
Integrin β3/CD61 : FITC
CD3 : BV510
skin ILC2
Skin ILC2
Skin Eos
F
BM
ILC2 19.6
ILC2 4.96
ST2 : FITC
CD25 : PE-CF594
BM ILC2
BM Eos

fig. S15: Inducible ILC2 deletion in BIC-DTR mice restricts homeostatic tissue eosinophilia

(A) Schematic of the experimental procedure to temporally induce ILC2 deletion by treatment of BIC-DTR mice with DTx.

(B) – (F) Flow cytometric analyses and quantification of ILC2s (defined by gating strategy in fig. S2) and eosinophils (defined as viable $CD45^{+}CD11b^{+}SiglecF^{+}$cells) in various tissues including the (B) lung, (C) small intestine lamina propria, (D) adipose tissue), (E) skin and (F) bone marrow. Data are pooled from at least 2 independent experiments and represent mean ± SD; unpaired two-sided t-test; *P<0.05; **P<0.01; ***P<0.001; ****P<0.0001; individual data point denotes biological replicates; n=6 mice (lung, siLP, bone marrow), n=9-10 mice (adipose tissue), n=26 mice (skin).

Fig. S16

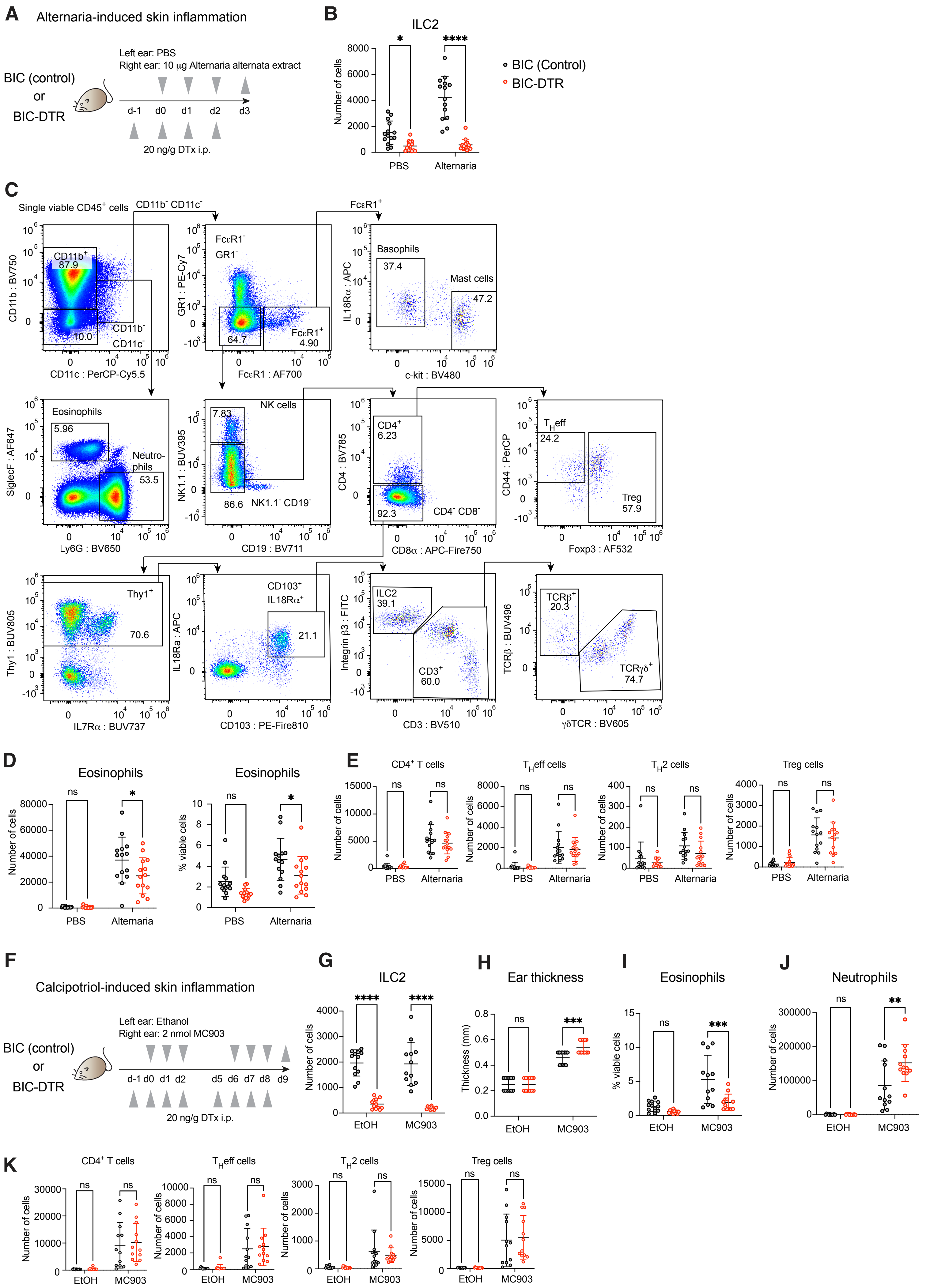

A Alternaria-induced skin inflammation
BIC (control) or BIC-DTR
Left ear: PBS
Right ear: 10 μg Alternaria alternata extract
d-1 d0 d1 d2 d3
20 ng/g DTx i.p.
B ILC2
Number of cells
PBS Alternaria
BIC (Control)
BIC-DTR
C
Single viable CD45+ cells
CD11b- CD11c-
FcεR1+
CD11b : BV750
CD11c : PerCP-Cy5.5
CD11b+ 87.9
CD11b- CD11c- 10.0
GR1 : PE-Cy7
FcεR1 : AF700
FcεR1- GR1- 64.7
FcεR1+ 4.90
IL18Rα : APC
c-kit : BV480
Basophils 37.4
Mast cells 47.2
SiglecF : AF647
Ly6G : BV650
Eosinophils 5.96
Neutrophils 53.5
NK1.1 : BUV395
CD19 : BV711
NK cells 7.83
86.6 NK1.1- CD19-
CD4 : BV785
CD8α : APC-Fire750
CD4+ 6.23
92.3 CD4- CD8-
CD44 : PerCP
Foxp3 : AF532
THeff 24.2
Treg 57.9
Thy1 : BUV805
IL7Rα : BUV737
Thy1+ 70.6
IL18Ra : APC
CD103 : PE-Fire810
CD103+ IL18Rα+ 21.1
Integrin β3 : FITC
CD3 : BV510
ILC2 39.1
CD3+ 60.0
TCRβ : BUV496
γδTCR : BV605
TCRβ+ 20.3
TCRγδ+ 74.7
D Eosinophils
Number of cells
% viable cells
PBS Alternaria
E CD4+ T cells
THeff cells
TH2 cells
Treg cells
F Calcipotriol-induced skin inflammation
Left ear: Ethanol
Right ear: 2 nmol MC903
d-1 d0 d1 d2 d5 d6 d7 d8 d9
G ILC2
H Ear thickness
Thickness (mm)
I Eosinophils
J Neutrophils
EtOH MC903
K CD4+ T cells
THeff cells
TH2 cells
Treg cells
ns

fig. S16: Inflammatory dermal eosinophilia, but not neutrophilia, require skin ILC2s

(A) Schematic of the experimental procedure to temporally induce ILC2 deletion in the context of an *A. alternata* extract-mediated skin inflammation model.

(B) Flow cytometric analyses and quantification of skin ILC2s from mice treated in (A), showing effective DTx-mediated ILC2 ablation.

(C) Gating strategy for immune cells from inflamed skin.

(D) Flow cytometric analyses and quantification of skin eosinophils from mice treated in (A). Decrease in skin eosinophil infiltration was associated with ILC2 ablation.

(E) Flow cytometric quantification of skin $CD4^+$ T cells, effector T ($T_H$eff) cells, T_H2 cells and regulatory T (Treg) cells from mice treated in (A). These cell types were not affected by ILC2 ablation.

(B, D and E) Data pooled from 3 independent experiments and represent mean ± SD; n=14 mice in each group in total.

(F) Schematic of the experimental procedure to temporally induce ILC2 deletion in the context of a calcipotriol-mediated skin inflammation model.

(G) Flow cytometric analyses and quantification of skin ILC2s from mice treated in (F), showing effective DTx-mediated ILC2 ablation. EtOH, ethanol.

(H) Measurement of ear thickness (day 8) after calcipotriol treatment. Increased ear thickness is associated with ILC2 ablation.

(I and J) Flow cytometric analyses and quantification of skin eosinophils (I) and neutrophils (J) after calcipotriol treatment (day 9). ILC2 ablation resulted in a shift from eosinophilia to neutrophilia in the absence of ILC2-derived type-2 signals.

(K) Flow cytometric quantification of skin $CD4^+$ T cells, effector T ($T_H$eff) cells, T_H2 cells and regulatory T (Treg) cells after calcipotriol treatment (day 9). These cell types were not affected by ILC2 ablation.

(F) – (K) Data pooled from 2 independent experiments and represent mean ± SD; n=12 mice in each group in total.

Significance in (B) - (K) was determined using one-way ANOVA followed by Šidák's multiple comparisons test between the indicated groups; ns, not significant; **P<0.01; ***P<0.001; ****P<0.0001; individual data point denotes biological replicates.

Fig. S17

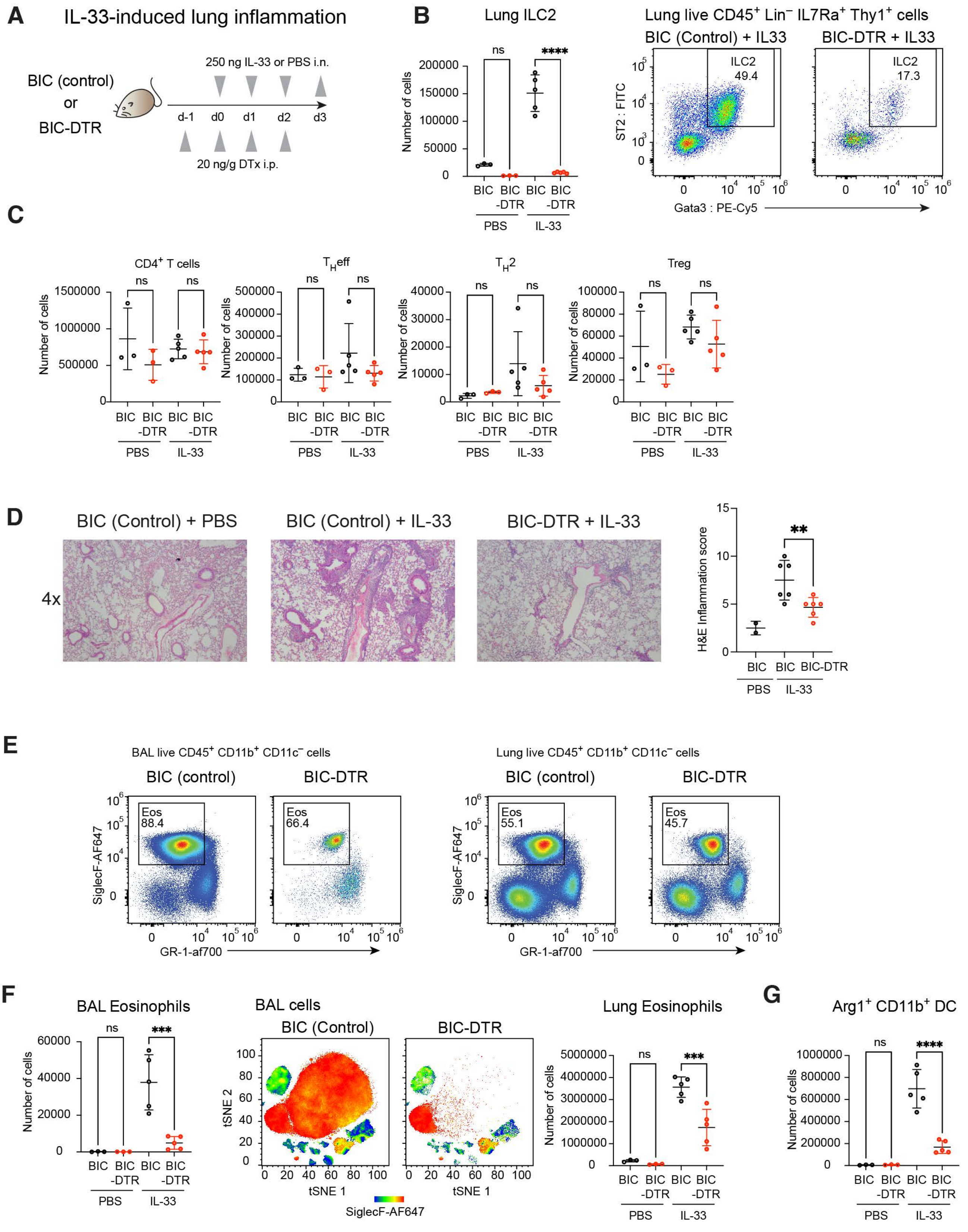

A
IL-33-induced lung inflammation
BIC (control)
or
BIC-DTR
250 ng IL-33 or PBS i.n.
d-1
d0
d1
d2
d3
20 ng/g DTx i.p.
B
Lung ILC2
Number of cells
ns
****
PBS
IL-33
Lung live CD45+ Lin− IL7Ra+ Thy1+ cells
BIC (Control) + IL33
BIC-DTR + IL33
ILC2
49.4
ILC2
17.3
ST2 : FITC
Gata3 : PE-Cy5
C
CD4+ T cells
THeff
TH2
Treg
Number of cells
D
BIC (Control) + PBS
BIC (Control) + IL-33
BIC-DTR + IL-33
4x
H&E Inflammation score
**
E
BAL live CD45+ CD11b+ CD11c− cells
Lung live CD45+ CD11b+ CD11c− cells
BIC (control)
BIC-DTR
Eos
88.4
Eos
66.4
Eos
55.1
Eos
45.7
SiglecF-AF647
GR-1-af700
F
BAL Eosinophils
***
BAL cells
BIC (Control)
BIC-DTR
tSNE 2
tSNE 1
SiglecF-AF647
Lung Eosinophils
G
Arg1+ CD11b+ DC
****

fig. S17: ILC2 ablation potently reduces IL-33-mediated acute lung inflammation

(A) Schematic of the experimental procedure to temporally induce ILC2 deletion in the context of an IL-33-mediated lung inflammation model.

(B) Flow cytometric analyses and quantification of lung ILC2s from mice treated in (A).

(C) Flow cytometric quantification of lung $CD4^+$ T cells, effector T ($T_H$eff) cells, T_H2 cells and regulatory T (Treg) cells in BIC control or BIC-DTR mice treated with PBS or IL-33. These cell types were not affected by ILC2 ablation in this acute model.

(D) Histological assessment of lung inflammation by H&E staining. ILC2 ablation resulted in reduced lung inflammation. Data pooled from 2 independent experiments and represent 6 biologically independent samples in each group.

(E) & (F) Flow cytometric analyses and quantification of bronchoalveolar lavage (BAL) and lung eosinophils in BIC control or BIC-DTR mice treated intranasally with IL-33. ILC2 ablation resulted in reductions in lung and bronchoalveolar eosinophils. High parametric flow cytometry of samples from the BAL were also interrogated with tSNE analysis (F) using all recorded fluorescent parameters (with the exception of viability dye and CD45) to generate cell clusters represented in two dimensions by tSNE-1 and tSNE-2. Expression of SiglecF by each cluster is displayed as heatmap and demonstrates the profound deficiency of eosinophils when ILC2 are deleted.

(G) Flow cytometric analyses and quantification of lung $Arg1^+CD11b^+$ dendritic cells (DCs) in BIC control or BIC-DTR mice treated intranasally with IL-33. ILC2 ablation resulted in reduced in lung $Arg1^+CD11b^+$ DCs. Gating strategy for $Arg1^+CD11b^+$ DCs shown in fig. S4B.

(B, C, D, F) Data are representative of 2 independent experiments and represent mean ± SD; n=3 mice in PBS groups, n=5 mice in IL-33 treatment groups in each experiment; one-way ANOVA followed by Šidák's multiple comparisons test between the indicated groups; ns, not significant; **P<0.01; ***P<0.001; ****P<0.0001; individual data point denotes biological replicates.

Fig. S18

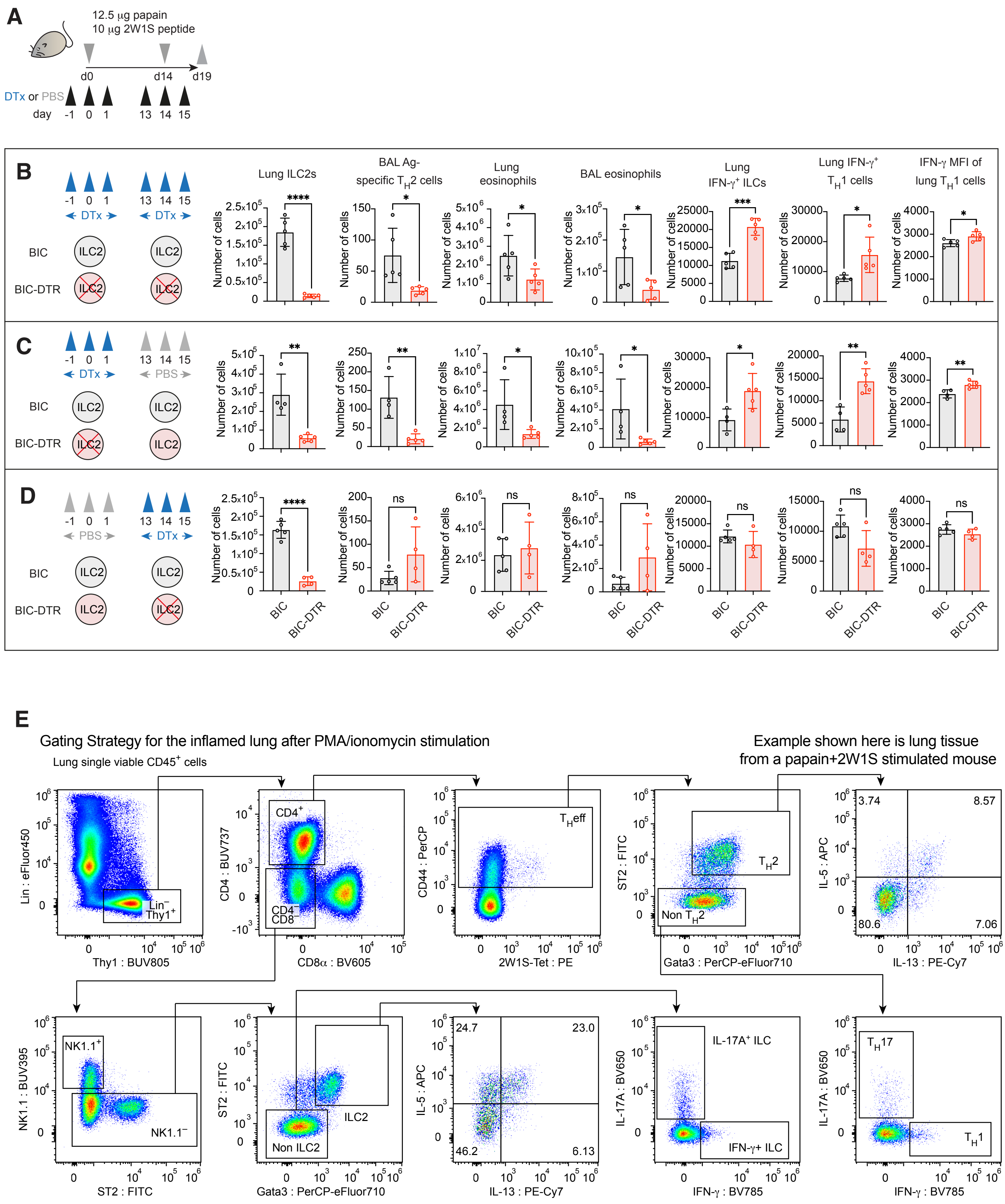

A
12.5 μg papain
10 μg 2W1S peptide
d0
d14
d19
DTx or PBS
day -1 0 1 13 14 15
B
DTx
BIC
BIC-DTR
ILC2
Lung ILC2s
BAL Ag-specific TH2 cells
Lung eosinophils
BAL eosinophils
Lung IFN-γ+ ILCs
Lung IFN-γ+ TH1 cells
IFN-γ MFI of lung TH1 cells
Number of cells
C
DTx
PBS
D
PBS
DTx
ns
BIC
BIC-DTR
E
Gating Strategy for the inflamed lung after PMA/ionomycin stimulation
Lung single viable CD45+ cells
Example shown here is lung tissue from a papain+2W1S stimulated mouse
Lin : eFluor450
Thy1 : BUV805
Lin− Thy1+
CD4 : BUV737
CD8α : BV605
CD4+
CD4− CD8−
CD44 : PerCP
2W1S-Tet : PE
THeff
ST2 : FITC
Gata3 : PerCP-eFluor710
TH2
Non TH2
IL-5 : APC
IL-13 : PE-Cy7
3.74
8.57
80.6
7.06
NK1.1 : BUV395
ST2 : FITC
NK1.1+
NK1.1−
ILC2
Non ILC2
24.7
23.0
46.2
6.13
IL-17A : BV650
IFN-γ : BV785
IL-17A+ ILC
IFN-γ+ ILC
TH17
TH1

fig. S18: Primary and secondary allergic lung challenge in BIC-DTR mice

(A) Schematic of the experimental timeline used to investigate the role of ILC2 in the initiation and recall phase of the response to papain and 2W1S peptide challenge, with varying Diphtheria toxin (DTx) or PBS treatment.

(B – D) Quantification of flow cytometric analysis of the indicated cell populations from the lung and bronchoalveolar lavage (BAL) from mice as treated in (A). DTx treatment during both the sensitisation and recall phases (B); DTx treatment and ILC2 ablation during the sensitisation phase only (C); DTx treatment and ILC2 ablation during the recall phase only (D). The timing of ILC2 ablation markedly influenced the outcome of the immune reaction. ILC2 depletion during both primary and secondary challenge (B), or only during primary stimulation (D), resulted in reduced antigen-specific T_H2 cell responses and impaired pulmonary eosinophilia. In both regimens we also observed a reciprocal increase in type-1 IFN-γ responses when ILC2s were ablated. By contrast, ILC2 depletion only before the rechallenge of the lungs with papain (D) did not diminish the T_H2 cell recall response, or the levels of eosinophilia, or increase IFN-γ responses. Data are representative of 2 similar experiments and represent mean ± SD; n=4-5 mice in each group; unpaired two-sided t-test; ns, not significant; *P<0.05; **P<0.01; ***P<0.001; ****P<0.0001; individual data point denotes biological replicates.

(E) Gating strategy for cytokine production from PMA/ionomycin stimulated immune cells from inflamed lung. Lineage staining: CD11b, CD11c, CD19, FcεR1, GR-1, Ter119.

Fig. S19

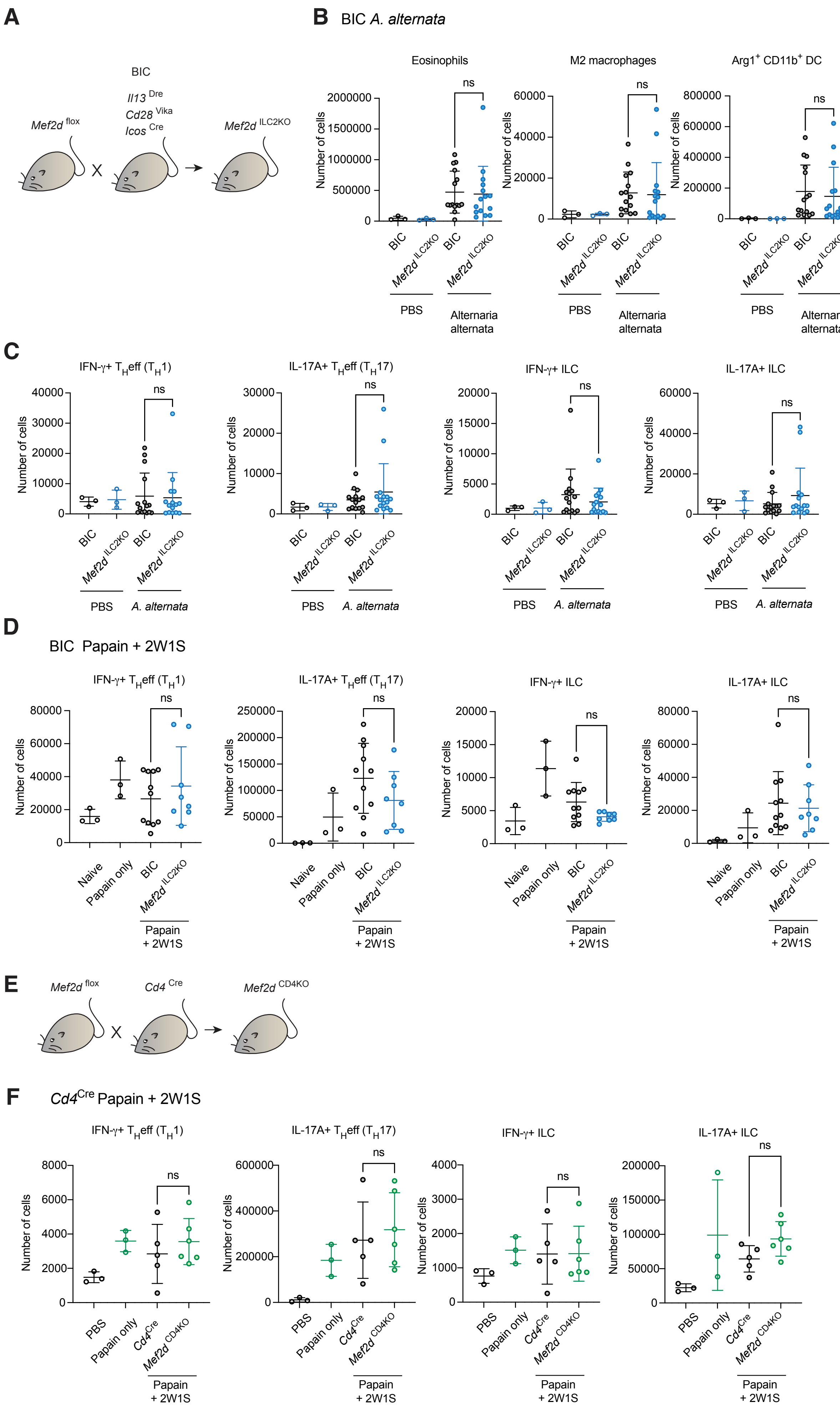
A
BIC
Mef2d flox
Il13 Dre
Cd28 Vika
Icos Cre
Mef2d ILC2KO
B BIC A. alternata
Eosinophils
M2 macrophages
Arg1+ CD11b+ DC
Number of cells
ns
BIC
Mef2d ILC2KO
PBS
Alternaria alternata
C
IFN-γ+ THeff (TH1)
IL-17A+ THeff (TH17)
IFN-γ+ ILC
IL-17A+ ILC
A. alternata
D
BIC Papain + 2W1S
Naive
Papain only
Papain + 2W1S
E
Mef2d flox
Cd4 Cre
Mef2d CD4KO
F
Cd4Cre Papain + 2W1S
PBS
Cd4Cre
Mef2d CD4KO

fig. S19: ILC2- or CD4-specific Mef2d deficiency did not affect type-1 or type-17 cytokine expression

(A) Schematic of the generation of *Mef2d*^{ILC2KO} mice by intercrossing *Mef2d*^{flox} mice with Boolean-ILC2-Cre (BIC) mice.

(B) Quantification of lung eosinophils, M2 macrophages and Arg1^{+} CD11b^{+} DCs from PBS or *Alternaria*-treated BIC or Mef2d^{ILC2KO} mice. Data are pooled from 3 independent experiments with n=15 mice in each group; mean ± SD.

(C) Quantification of IFN-γ- or IL-17A-expressing innate and T_H lymphocytes from PBS or *Alternaria*-treated BIC or Mef2d^{ILC2KO} mice. Data are pooled from 3 independent experiments with n=15 mice in each group; mean ± SD.

(D) Quantification of IFN-γ- or IL-17A-expressing innate and T_H lymphocytes from naive, papain or papain+2W1S treated BIC or Mef2d^{ILC2KO} mice. Data are pooled from 2 independent experiments and represent mean ± SD; n=3 in naïve and papain only groups, n=11 in papain+2W1S-treated control group, n=8 in papain+2W1S-treated Mef2d^{ILC2KO} group.

(E) Schematic of the generation of *Mef2d*^{CD4KO} mice by intercrossing *Mef2d*^{flox} mice with *Cd4*^{Cre} mice.

(F) Quantification of IFN-γ- or IL-17A-expressing innate and T_H lymphocytes from PBS, papain or papain+2W1S *Cd4*^{Cre} or *Mef2d*^{CD4KO} treated mice. Data are representative of 2 independent experiments and represent mean ± SD; n=3 in naïve and papain only groups, n=6 in papain+2W1S-treated groups.

Significance in (B) - (F) was determined using one-way ANOVA with Šidák's multiple comparisons test between the indicated groups; ns, not significant; individual data point denotes biological replicates.

Fig. S20

A *In vitro* cultured ILC2s

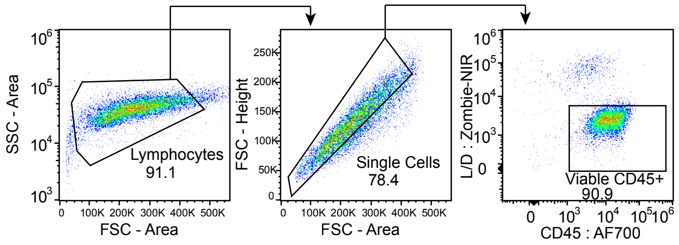

B

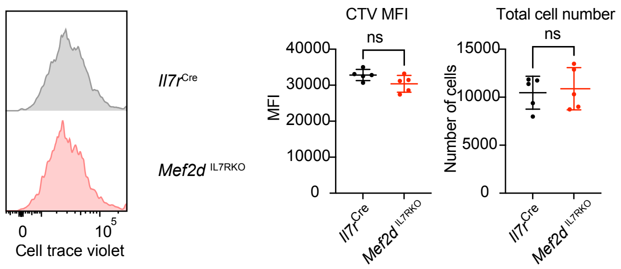

C

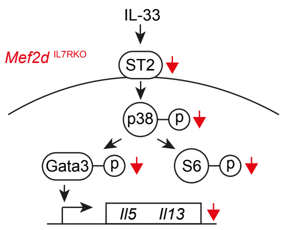

D Phenotyping of intestinal innate lymphocytes

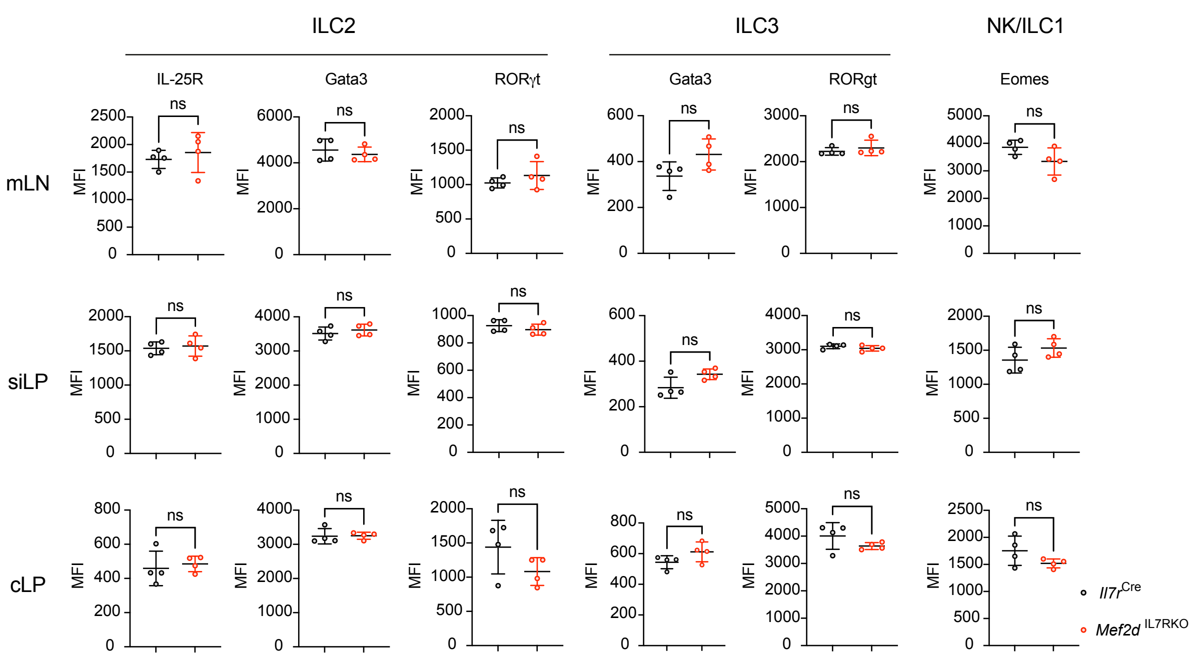

E Phenotyping of ILC2 from various tissues

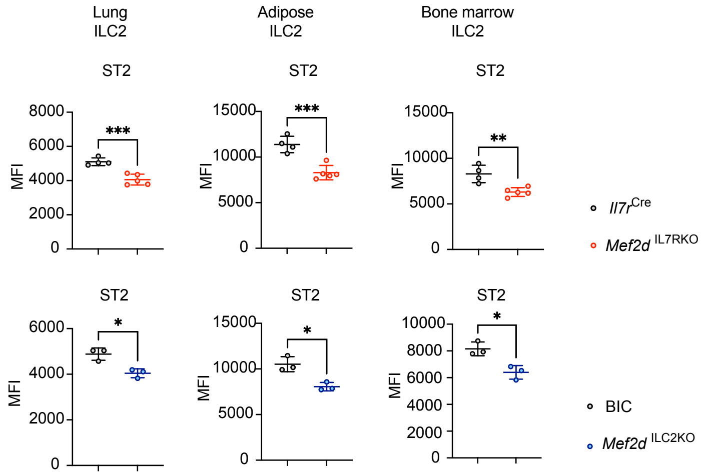

fig. S20: Mef2d-deficient ILC2 are defective in ST2 expression but retained normal IL-25R expression.

(A) Representative gatin strategy for the analysis of live cultured ILC2s.

(B) Flow cytometric quantification of cell trace violet MFI and cell numbers of following 3 days of culturing purified ILC2s in the presence of IL-7 & IL-33. Data are representative of 2 independent experiments with n=5 biologically independent samples in each experiment; mean ± SD.

(C) Schematic to indicate Mef2d-deficiency in ILC2s is associated with a reduction in the activation of downstream signalling molecules including phospho-p38 (Fig. 4C), phospho-S6 (Fig. 4D) and phospho-GATA3 (Fig. 4E), which are known downstream mediators in the IL-33/ST2-mediated signalling cascade.

(D) Quantification of transcription factor and IL-25R expression by intestinal ILC subsets of *Il7r*^{Cre} or *Mef2d*$^{\mathrm{IL7RKO}}$ mice. Data are representative of 2 independent experiments with n=4 mice per group in each experiment; mean ± SD.

(E) Quantification of ST2 expression by ILC2 from the indicated tissues comparing *Il7r*^{Cre} versus *Mef2d*$^{\mathrm{IL7RKO}}$ mice (left), or comparing BIC versus *Mef2d*$^{\mathrm{ILC2KO}}$ mice (right). Data are representative of 2 independent experiments with n=3-5 mice per group in each experiment; mean ± SD.

Significance in (A) - (D) was determined using unpaired two-sided t-test; ns, not significant; *P<0.05; **P<0.01; ***P<0.001; individual data point denotes biological replicates.

Fig. S21

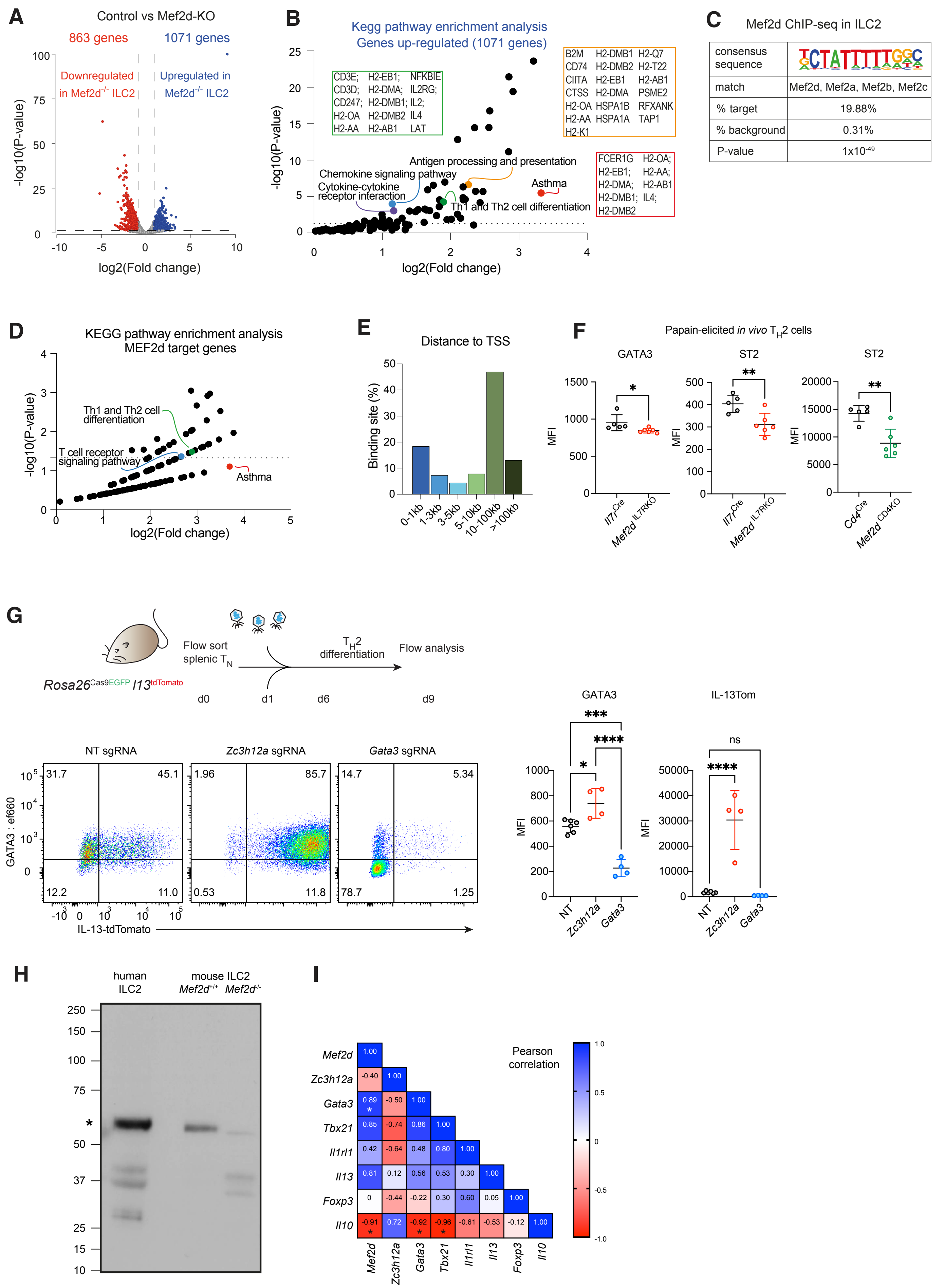

A
Control vs Mef2d-KO
863 genes
1071 genes
Downregulated in Mef2d-/- ILC2
Upregulated in Mef2d-/- ILC2
-log10(P-value)
log2(Fold change)
B
Kegg pathway enrichment analysis
Genes up-regulated (1071 genes)
CD3E; H2-EB1; NFKBIE
CD3D; H2-DMA; IL2RG;
CD247; H2-DMB1; IL2;
H2-OA H2-DMB2 IL4
H2-AA H2-AB1 LAT
B2M H2-DMB1 H2-Q7
CD74 H2-DMB2 H2-T22
CIITA H2-EB1 H2-AB1
CTSS H2-DMA PSME2
H2-OA HSPA1B RFXANK
H2-AA HSPA1A TAP1
H2-K1
FCER1G H2-OA;
H2-EB1; H2-AA;
H2-DMA; H2-AB1
H2-DMB1; IL4;
H2-DMB2
Antigen processing and presentation
Chemokine signaling pathway
Cytokine-cytokine receptor interaction
Asthma
Th1 and Th2 cell differentiation
C
Mef2d ChIP-seq in ILC2
consensus sequence
match
Mef2d, Mef2a, Mef2b, Mef2c
% target
19.88%
% background
0.31%
P-value
1x10-49
D
KEGG pathway enrichment analysis
MEF2d target genes
Th1 and Th2 cell differentiation
T cell receptor signaling pathway
Asthma
E
Distance to TSS
Binding site (%)
0-1kb
1-3kb
3-5kb
5-10kb
10-100kb
>100kb
F
Papain-elicited in vivo TH2 cells
GATA3
ST2
ST2
MFI
Il7rCre
Mef2d IL7RKO
Cd4Cre
Mef2d CD4KO
G
Rosa26Cas9EGFP Il13tdTomato
Flow sort splenic TN
TH2 differentiation
Flow analysis
d0
d1
d6
d9
NT sgRNA
Zc3h12a sgRNA
Gata3 sgRNA
31.7
45.1
12.2
11.0
1.96
85.7
0.53
11.8
14.7
5.34
78.7
1.25
GATA3 : ef660
IL-13-tdTomato
GATA3
IL-13Tom
ns
NT
Zc3h12a
Gata3
H
human ILC2
mouse ILC2
Mef2d+/+
Mef2d-/-
250
150
100
75
50
37
25
15
10
I
Pearson correlation
Mef2d
Zc3h12a
Gata3
Tbx21
Il1rl1
Il13
Foxp3
Il10

fig. S21: The Mef2d-Regnase-1-GATA3 pathway in mouse T_H2 cells, and the correlation of Mef2d and GATA3 expression in human ILC2s

(A) Volcano plot showing differentially expressed genes from RNA-sequencing analysis of *Mef2d*^{IL7RKO} versus *Il7r*^{Cre} control ILC2s.

(B) KEGG pathway analysis of genes upregulated in *Mef2d*^{IL7RKO} ILC2s versus *Il7r*^{Cre} control ILC2s.

(C) Motif enrichment in Mef2d ChIP-seq peaks using *de novo* motif analysis. Enrichment was assessed using a one-sided cumulative binomial distribution in HOMER.

(D) KEGG pathway analysis of the genes located less than 10kb from a Mef2d binding site.

(E) Distribution of the distance of Mef2d ChIP-seq peaks to the closest transcription start site (TSS).

(F) Quantification of ST2 and GATA3 MFI of T_H2 cells from control or conditional Mef2d-deficient papain and 2W1S-challenged mice. Data are representative of 2 independent experiments and represent mean ± SD; unpaired two-sided t-test; *P<0.05; **P<0.01; n=5 control mice and n=6 Mef2d^{IL7RKO} mice; individual data point denotes biological replicates.

(G) Schematic of the experimental procedure to produce *Gata3* or *Zc3h12a* CRISPR-KO T_H2 cells and flow cytometric quantification of their GATA3 and IL-13Tom MFI. Data are representative of 2 independent experiments with n=2 biologically independent samples in each experiment and 4 different sgRNAs targeting each gene; mean ± SD; one-way ANOVA with Tukey's post-hoc test; ns, not significant; *P<0.05; ***P<0.001; ****P<0.0001; individual data point denotes different sgRNAs targeting the indicated gene.

(H) Western blot analysis of Mef2d protein in expanded human ILC2 lysate. Mouse ILC2s sufficient and deficient in Mef2d serve as positive and negative controls, respectively.

(I) Pearson correlation analysis of the indicated transcripts expressed by human ILC2s cultured in a variety of polarising conditions. Significant correlations are indicated with an asterisk (*).

Fig. S22

A

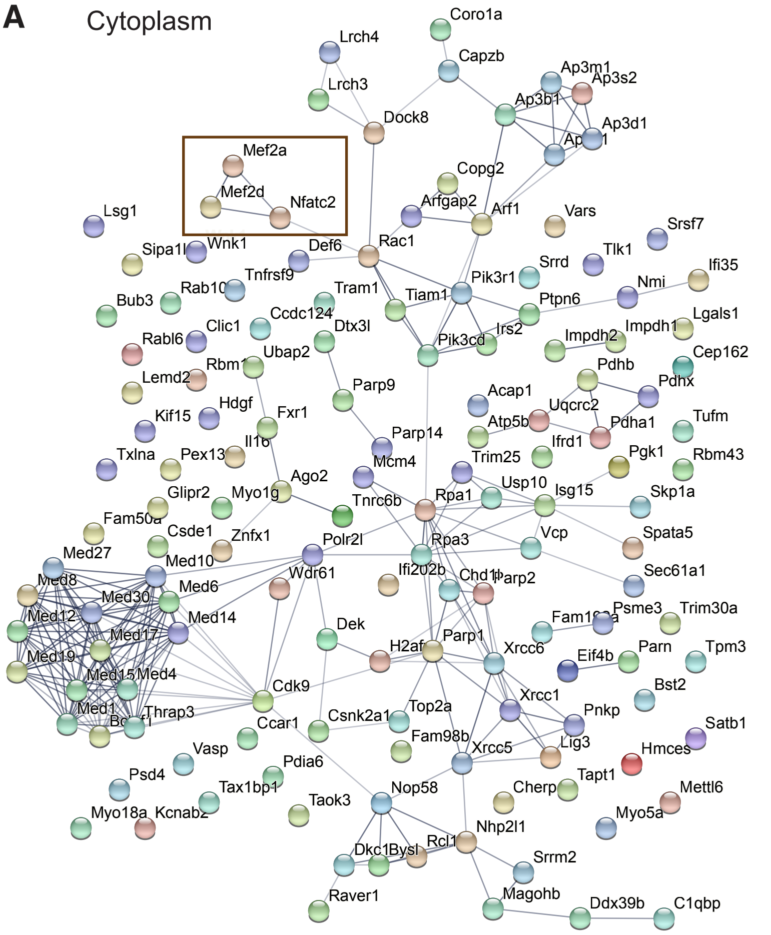

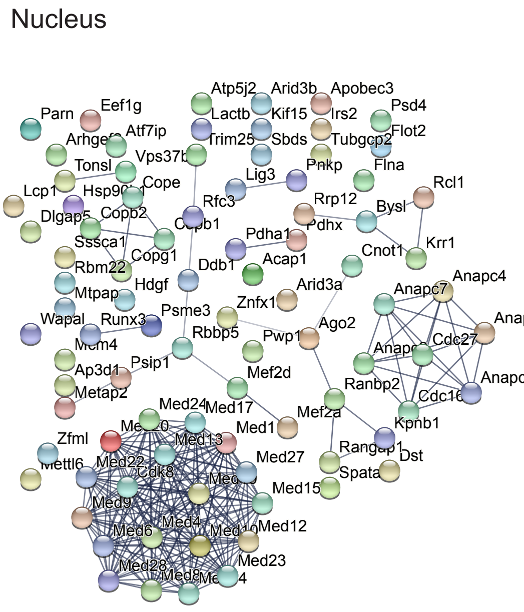

B

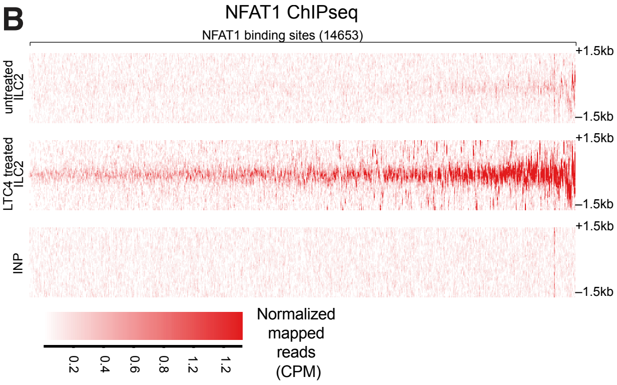

C

NFAT1 ChIP-seq in ILC2

consensus sequence	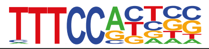
match	NFATC1, NFATC2, NFATC3
% target	10.03%
% background	3.41%
P-value	1x10-332

D

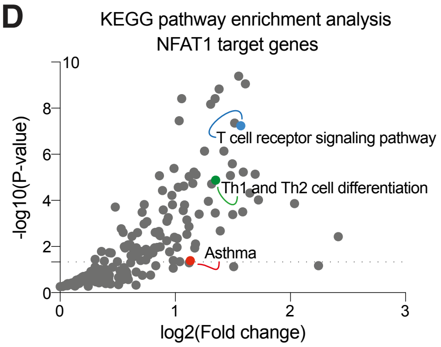

E

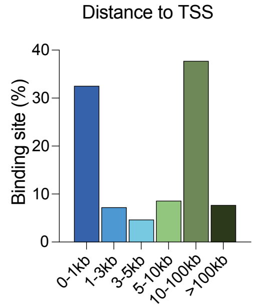

fig. S22: Unbiased identification of Mef2d-interacting proteins in ILC2s identifies a role for Mef2d-NFAT1 complexes in ILC2 function

(A) Network analysis of Mef2d-interacting proteins identified using mass spectrometry of proteins co-immunoprecipitated with anti-Mef2d antibody from ILC2 cytoplasmic (left) or nuclear (right) lysate.

(B) Heatmap representation of NFAT1 binding around the centre (±1.5 kb) of the NFAT1 peaks in ILC2s with and without LTC4 stimulation, ordered according to LTC4 treated sample.

(C) Top enriched motif in NFAT1 ChIP-seq peaks using in *de novo* motif analysis. Enrichment was assessed using a one-sided cumulative binomial distribution in HOMER.

(D) KEGG pathway analysis of the genes located less than 10kb from a NFAT1 binding site.

(E) Distribution of the distance of NFAT1 ChIP-seq peaks to the closest transcription start site (TSS).

Fig. S23

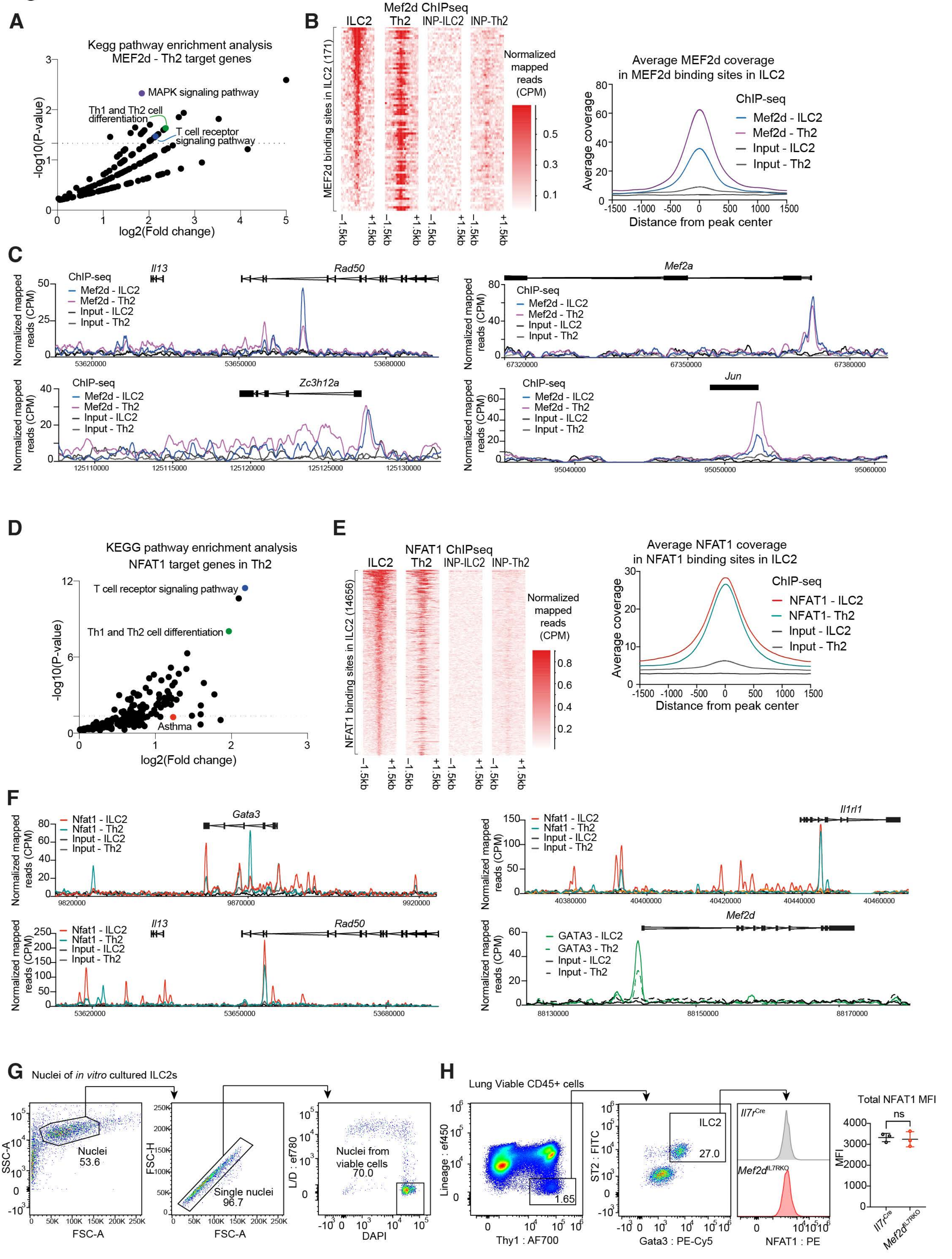

A
Kegg pathway enrichment analysis
MEF2d - Th2 target genes
MAPK signaling pathway
Th1 and Th2 cell differentiation
T cell receptor signaling pathway
-log10(P-value)
log2(Fold change)
B
Mef2d ChIPseq
ILC2
Th2
INP-ILC2
INP-Th2
MEF2d binding sites in ILC2 (171)
Normalized mapped reads (CPM)
Average MEF2d coverage in MEF2d binding sites in ILC2
ChIP-seq
Mef2d - ILC2
Mef2d - Th2
Input - ILC2
Input - Th2
Average coverage
Distance from peak center
C
Il13
Rad50
Mef2a
Zc3h12a
Jun
Normalized mapped reads (CPM)
D
KEGG pathway enrichment analysis
NFAT1 target genes in Th2
T cell receptor signaling pathway
Th1 and Th2 cell differentiation
Asthma
E
NFAT1 ChIPseq
NFAT1 binding sites in ILC2 (14656)
Average NFAT1 coverage in NFAT1 binding sites in ILC2
NFAT1 - ILC2
NFAT1- Th2
F
Nfat1 - ILC2
Nfat1 - Th2
Gata3
Il1rl1
Mef2d
GATA3 - ILC2
GATA3 - Th2
G
Nuclei of in vitro cultured ILC2s
Nuclei 53.6
Single nuclei 96.7
Nuclei from viable cells 70.0
SSC-A
FSC-A
FSC-H
L/D : ef780
DAPI
H
Lung Viable CD45+ cells
Lineage : ef450
Thy1 : AF700
1.65
ST2 : FITC
Gata3 : PE-Cy5
ILC2 27.0
Il7r Cre
Mef2d IL7RKO
NFAT1 : PE
Total NFAT1 MFI
ns
MFI

fig. S23: T_H2 cells and ILC2s share striking similarities in their genome-wide binding profiles of Mef2d, GATA3 and NFAT1.

(A) KEGG pathway analysis of the genes located less than 10kb from a Mef2d binding site in T_H2 cells.

(B) Left: heatmap representation of Mef2d binding around the centre (±1.5 kb) of the Mef2d peaks in ILC2s or T_H2 cells, ordered according to the ILC2 sample. Right: average ChIP-seq signal over all ILC2-Mef2d peaks in ILC2s and T_H2 cells. Data are representative of 2 biological replicates.

(C) Representative binding profiles of Mef2d in ILC2s (blue) or T_H2 cells (purple) at the *Il13*, *Rad50, Mef2a, Zc3h12a* and *Jun* loci. Data representative of 2 biological replicates.

(D) KEGG pathway analysis of the genes located less than 10kb from a NFAT1 binding site in T_H2 cells.

(E) Left: heatmap representation of NFAT1 binding around the centre (±1.5 kb) of the NFAT1 peaks in ILC2s or T_H2 cells, ordered according to the ILC2 sample. Right: average ChIP-seq signal over all ILC2-NFAT1 peaks in ILC2s and T_H2 cells. Data are representative of 2 biological replicates.

(F) Representative binding profiles of NFAT1 in ILC2s (red) or T_H2 cells (blue) at the *Gata3*, *Il1rl1*, *Il13* and *Rad50* loci, and GATA3 in ILC2s (filled line) or T_H2 cells (dashed line) at the *Mef2d* locus. Data representative of 2 biological replicates.

(G) Representative flow cytometric gating strategy for the analysis of nuclei isolated from ILC2s cultured *in vitro* following stimulation of LTC4.

(H) Flow cytometric analysis of total NFAT1 protein in lung ILC2s from *Il7r*^{Cre} and *Mef2d*^{IL7RKO} mice. Data from 1 experiment with n=3 mice in each group; mean ± SD; unpaired two-sided t-test; ns, not significant; individual data point denotes biological replicates.

Fig. S24

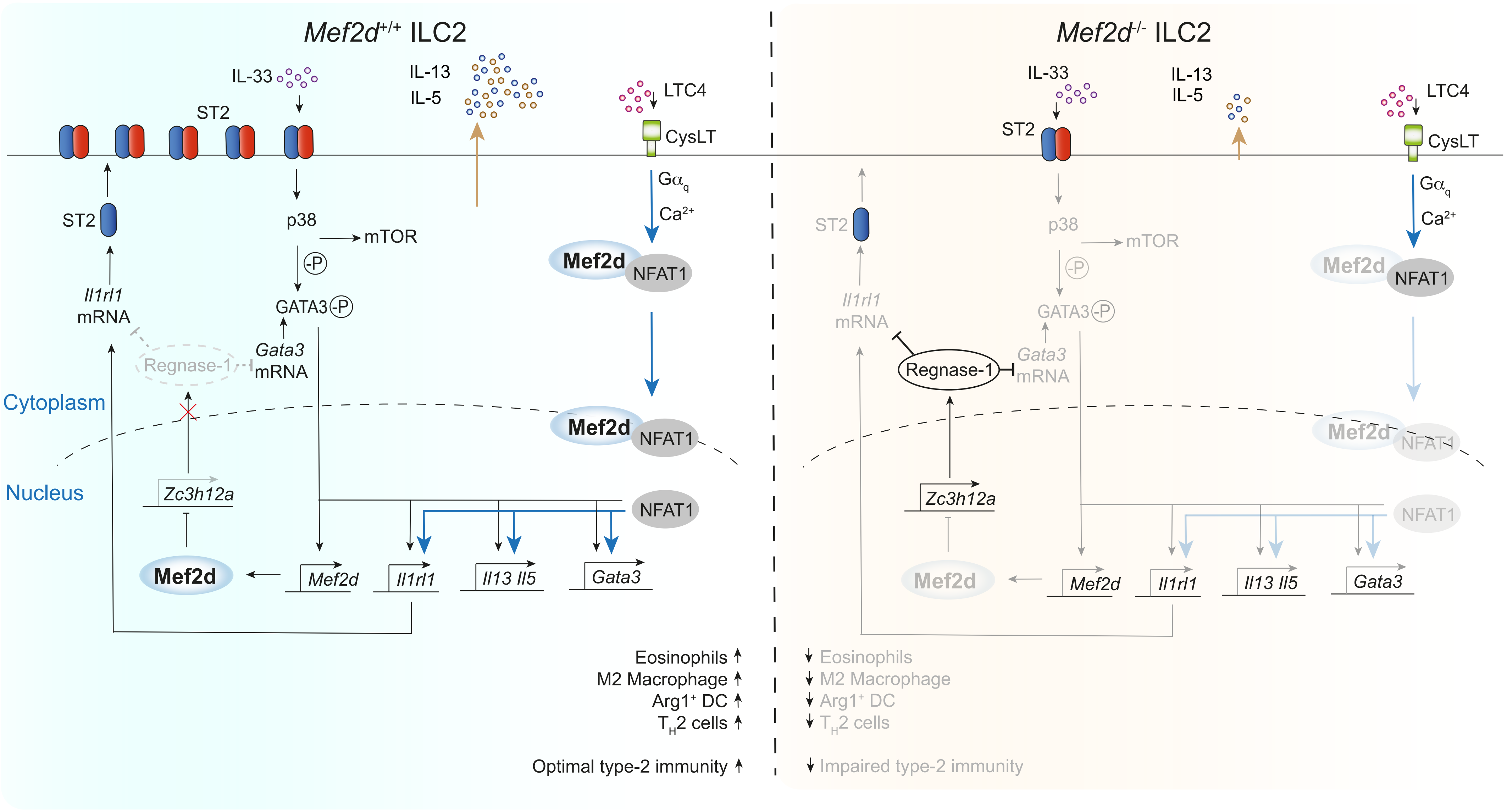

Mef2d+/+ ILC2
IL-33
ST2
IL-13
IL-5
LTC4
CysLT
Gαq
Ca2+
p38
mTOR
-P
GATA3-P
ST2
Il1rl1
mRNA
Regnase-1
Gata3
mRNA
Mef2d
NFAT1
Cytoplasm
Nucleus
Zc3h12a
Mef2d
Il1rl1
Il13 Il5
Gata3
Eosinophils
M2 Macrophage
Arg1+ DC
TH2 cells
Optimal type-2 immunity
Mef2d-/- ILC2
Eosinophils
M2 Macrophage
Arg1+ DC
TH2 cells
Impaired type-2 immunity

fig. S24: Proposed model for the role of Mef2d in ILC2 function.
Mef2d regulates two pathways:1) Regnase-1/GATA3/ST2/IL-33 signalling and 2) calcium signalling which converge to control ILC2 function.

Table S1 – primers for generating the BIC alleles

Guide RNA target sequences

Locus	Guide	Orientation	Target Sequence (including PAM)
Icos	G1	Forward	tgcaggtacagctcccctta cgg
Il13	G1	Reverse	cacatcaccttgagtgtaac agg
Il13	G2	Forward	aggtgatgtgtagtgaatgg ggg
Cd28	G1	Reverse	gccggcgggcttctggatag ggg

Amplification and sequencing primer sequences

Locus	Primer	Sequence
Icos	5’ Probe forward primer	TGAGCCAAACTCAGCTAAAGG
Icos	5’ Probe reverse primer	TTCACTGCCAGGTGAGTTCC
Icos	3’ Probe forward primer	GGACCACAGGGCACCTGACTTG
Icos	3’ Probe reverse primer	GGCTGAGCTTCCTATTTGGAG
Il13	5’ Probe forward primer	ACTCTGGCCTTCTCTGTAGACCCCC
Il13	5’ Probe reverse primer	GGGGCTTAGAGAAAAGAAGCCAACAGCCAATAACCGCC
Il13	3’ Probe forward primer	TCAGGGACCGCTCAAACTCTGAATGACAGCAGGAGCC
Il13	3’ Probe reverse primer	CCAGCAGGAAAGATGGAGGAGACATCC
Cd28	3’ Probe forward primer	TGTTAGTATTTGTCAGGGTGGTAAGG
Cd28	3’ Probe reverse primer	ACTTGGATGAACAGTGACTTGCC
Cd28	Amplification primer P1	CCCAGCCATTTCCATCCCATTGG
Cd28	Amplification primer P2	GAACTCGTCGGGTTCCAGGTGTTCC
Cd28	Sequencing primer P3	AAGCCTTATTCTAAGCAG
Cd28	Sequencing primer P4	TAGATTAAACACTCCCAC
Cd28	Sequencing primer P5	GTTGATAGTCAGGCTTTG
Cd28	Sequencing primer P6	CAATATGGAAGGACTTAG

Table S2 – flow cytometry antibodies

Antibody target	Clone	Colour	Source	Catalog number	RRID
Anti-mouse CD3ε	145-2C11	FITC	BioLegend	100306	AB_312670
Anti-mouse CD3ε	145-2C11	BV510	BioLegend	100353	AB_2565879
Anti-mouse CD3ε	145-2C11	PE-Cy7	BioLegend	100320	AB_312684
Anti-mouse CD4	GK1.5	BV421	BioLegend	100438	AB_10900241
Anti-mouse CD4	GK1.5	BV605	BioLegend	100451	AB_2564591
Anti-mouse CD4	GK1.5	BV650	BioLegend	100469	AB_2783035
Anti-mouse CD4	GK1.5	PE-Cy7	BioLegend	100422	AB_312706
Anti-mouse CD4	RM4-5	BV785	BioLegend	100551	AB_11218992
Anti-mouse CD8α	53-6.7	BV421	BioLegend	100738	AB_2562558
Anti-mouse CD8α	53-6.7	BV605	BioLegend	100744	AB_2562609
Anti-mouse CD8α	53-6.7	BV785	BioLegend	100750	AB_11218801
Anti-mouse CD8α	53-6.7	Alexa Fluor 700	BioLegend	100730	AB_493703
Anti-mouse CD8α	53-6.7	APC-Fire750	BioLegend	100766	AB_2572113
Anti-mouse CD11b	M1/70	PE-Cy7	BioLegend	101216	AB_312799
Anti-mouse CD11b	M1/70	BV750	BioLegend	101267	AB_2810328
Anti-mouse CD19	6D5	BV605	BioLegend	115540	AB_11203538
Anti-mouse CD19	6D5	BV711	BioLegend	115555	AB_2565970
Anti-mouse CD19	6D5	Alexa Fluor 700	BioLegend	115528	AB_493735
Anti-mouse CD25	PC61	BV510	BioLegend	102042	AB_2562269
Anti-mouse CD28	37.51	BV421	BioLegend	102127	AB_2650628
Anti-mouse/human CD44	IM7	BV605	BioLegend	103047	AB_2562451
Anti-mouse/human CD44	IM7	PerCP/Cy5.5	BioLegend	103031	AB_2076204
Anti-mouse/human CD44	IM7	PerCP	BioLegend	103036	AB_10645506
Anti-mouse CD45	30-F11	BV510	BioLegend	103138	AB_2563061
Anti-mouse CD45	30-F11	BV785	BioLegend	103149	AB_2564590
Anti-mouse CD45	30-F11	APC-Fire810	BioLegend	103174	AB_2860600
Anti-mouse CD45.2	104	BV650	BioLegend	109836	AB_2563065
Anti-mouse CD51	RMV-7	Biotin	BioLegend	104104	AB_313073
Anti-mouse CD51	RMV-7	PE	BioLegend	104106	AB_2129493
Anti-mouse/rat CD61	2C9.G2	Biotin	BioLegend	104304	AB_313081

Anti-mouse/rat CD61	2C9.G2	FITC	BioLegend	104306	AB_313083
Anti-mouse CD62L	MEL-14	BV421	BioLegend	104436	AB_2562560
Anti-mouse CD62L	MEL-14	BV570	BioLegend	104433	AB_10900262
Anti-mouse CD90.2 (Thy1.2)	30-H12	Alexa Fluor 700	BioLegend	105320	AB_493725
Anti-mouse CD127/IL7Rα	SB/199	Biotin	BioLegend	121104	AB_493502
Anti-mouse CD103	QA17A24	PE-Fire810	BioLegend	156919	AB_2924488
Anti-mouse F4/80	BM8	BV605	BioLegend	123133	AB_2562305
Anti-mouse FcεR1	MAR-1	Alexa Fluor 700	BioLegend	134324	AB_2566734
Anti-mouse Flt3 (CD135)	A2F10	APC	BioLegend	135310	AB_1953264
Anti-human CD2	RPA-2.10	BV510	BioLegend	300218	AB_2566040
Anti-human CD4	OKT4	PE	BioLegend	317410	AB_571955
Anti-mouse IL4	11B11	BV711	BioLegend	504133	AB_2565950
Anti-mouse/human IL-5	TRFK5	APC	BioLegend	504306	AB_315330
Anti-mouse/rat/human ICOS	C398.4A	PerCP-Cy5.5	BioLegend	313518	AB_10641280
Anti-mouse IL-17A	TC11-18H10.1	BV650	BioLegend	506930	AB_2686975
Anti-mouse IL18Rα	A17071D	APC	BioLegend	157906	AB_2860735
Anti-mouse IFNγ	XMG1.2	BV785	BioLegend	505838	AB_11219004
Anti-mouse Ly-6G	1A8	BV650	BioLegend	127641	AB_2565881
Anti-mouse MHC Class II (I-A/I-E)	M5/114.15.2	BV510	BioLegend	107636	AB_2734168
Anti-mouse MHC Class II (I-A/I-E)	M5/114.15.2	BV650	BioLegend	107641	AB_2565975
Anti-mouse Perforin	S16009B	APC	BioLegend	154404	AB_2721465
Anti-mouse Ly-6A/E (Sca-1)	D7	BV605	Biolegend	108134	AB_2650926
Anti-mouse/human T-bet	4B10	BV605	BioLegend	644817	AB_11219388
Anti-mouse TCRγδ	GL3	BV605	BioLegend	118129	AB_2563356
Anti-mouse TER-119	TER-119	Alexa Fluor 700	BioLegend	116220	AB_528963
Anti-mouse TNFα	MP6-XT22	PE	BioLegend	506306	AB_315427
Anti-mouse TNFα	MP6-XT22	Alexa Fluor 700	BioLegend	506338	AB_2562918
Anti-mouse/human Phospho-p38 MAPK	A16016A	PE	BioLegend	690204	AB_2876743
Anti-mouse/human Phospho-ERK1/2	4B11B69	Alexa Fluor 647	BioLegend	675504	AB_2571894

Anti-mouse/human Arginase 1	A1exF5	APC	eBioscience	17-3697-82	AB_2734835
Anti-mouse/human Arginase 1	A1exF5	eFluor 450	eBioscience	48-3697-82	AB_2734837
Anti-mouse CD3	17A2	eFluor 450	eBioscience	48-0032-82	AB_1272193
Anti-mouse CD3	17A2	Alexa Fluor 700	eBioscience	56-0032-82	AB_529507
Anti-mouse CD4	GK1.5	FITC	eBioscience	11-0041-82	AB_464892
Anti-mouse CD4	GK1.5	PE-Cy5	eBioscience	15-0041-82	AB_468695
Anti-mouse CD4	GK1.5	eFluor 450	eBioscience	48-0041-82	AB_10718983
Anti-mouse CD4	GK1.5	Alexa Fluor 700	eBioscience	56-0041-82	AB_493999
Anti-mouse CD8α	53-6.7	eFluor 450	eBioscience	48-0081-80	AB_1272235
Anti-mouse CD8α	53-6.7	FITC	eBioscience	11-0081-82	AB_464915
Anti-mouse CD8α	53-6.7	PE-Cy5	eBioscience	15-0081-82	AB_468706
Anti-mouse CD8α	53-6.7	PE-Cy7	eBioscience	25-0081-82	AB_469584
Anti-mouse CD11b	M1/70	eFluor 450	eBioscience	48-0112-82	AB_1582236
Anti-mouse CD11b	M1/70	Alexa Fluor 700	eBioscience	56-0112-82	AB_657585
Anti-mouse CD11c	N418	eFluor 450	eBioscience	48-0114-82	AB_1548654
Anti-mouse CD11c	N418	PerCP-Cy5.5	eBioscience	45-0114-82	AB_925727
Anti-mouse CD11c	N418	PE-Cy7	eBioscience	25-0114-82	AB_469590
Anti-mouse CD11c	N418	AF700	eBioscience	56-0114-82	AB_493992
Anti-mouse CD19	eBio1D3	eFluor 450	eBioscience	48-0193-82	AB_2734905
Anti-mouse CD19	eBio1D3	PerCP-Cy5.5	eBioscience	45-0193-82	AB_1106999
Anti-mouse CD19	eBio1D3	PE-Cy7	eBioscience	25-0193-82	AB_657663
Anti-mouse CD25	PC61.5	PE-Cy7	eBioscience	25-0251-82	AB_469608
Anti-mouse/human CD44	IM7	FITC	eBioscience	11-0441-82	AB_465045
Anti-mouse/human CD44	IM7	APC	eBioscience	17-0441-82	AB_469390
Anti-mouse CD45	30-F11	FITC	eBioscience	11-0451-82	AB_465050
Anti-mouse CD45	30-F11	Alexa Fluor 700	eBioscience	56-0451-82	AB_891454
Anti-mouse FceR1	MAR-1	eFluor 450	eBioscience	48-5898-82	AB_2574086
Anti-mouse FceR1	MAR-1	PE-Cy7	eBioscience	25-5898-82	AB_2573493
Anti-mouse CD135 (Flt3)	A2F10	PE	eBioscience	12-1351-82	AB_465859
Anti-mouse CD135 (Flt3)	A2F10	PerCP-eFluor 710	eBioscience	46-1351-82	AB_10733393
Anti-mouse Eomes	Dan11mag	eFluor 450	eBioscience	48-4875-82	AB_2574062

Anti-mouse/bovine/canine/feline/porcine/rat FoxP3	FJK-16s	eFluor 660	eBioscience	50-5773-82	AB_11218868
Anti-mouse/bovine/canine/feline/porcine/rat FoxP3	FJK-16s	Alexa Fluor 532	eBioscience	58-5773-82	AB_11218870
Anti-mouse/bovine/canine/feline/porcine/rat FoxP3	FJK-16s	PE-Cy7	eBioscience	25-5773-82	AB_891552
Anti-mouse/human/porcine Gata-3	TWAJ	eFluor 450	eBioscience	48-9966-42	AB_2811834
Anti-mouse/human/porcine Gata-3	TWAJ	eFluor 660	eBioscience	50-9966-42	AB_10596663
Anti-mouse/human/porcine Gata-3	TWAJ	PerCP-EF710	eBioscience	46-9966-42	AB_10804487
Anti-mouse/human/porcine Gata-3	TWAJ	PE-Cy5	eBioscience	15-9966-42	AB_2811756
Anti-mouse/human/porcine Gata-3	TWAJ	PE	eBioscience	12-9966-42	AB_1963600
Anti-mouse Ly-6G/Ly-6C	RB6-8C5	eFluor 450	eBioscience	48-5931-82	AB_1548788
Anti-mouse Ly-6G/Ly-6C	RB6-8C5	PE-Cy7	eBioscience	25-5931-82	AB_469663
Anti-mouse Ly-6G/Ly-6C	RB6-8C5	Alexa Fluor 700	eBioscience	56-5931-82	AB_494007
Anti-human/chimpanzee/baboon/porcine CD2	RPA-2.10	APC	eBioscience	17-0029-42	AB_10805740
Anti-mouse/rat/ rhesus monkey CD278 (ICOS)	C398.4A	APC	eBioscience	17-9949-82	AB_11149880
Anti-mouse CD127 (IL7Ra)	A7R34	PE-Cy7	eBioscience	25-1271-82	AB_469649
Anti-mouse IL13	eBio13A	Alexa Fluor 488	eBioscience	53-7133-82	AB_2016708
Anti-mouse IL13	eBio13A	PE	eBioscience	12-7133-82	AB_763559
Anti-mouse IL13	eBio13A	PE-Cy7	eBioscience	25-7133-82	AB_2573530
Anti-mouse/human/rat/canine Ki67	SolA15	BUV615	eBioscience	366-5698-82	AB_2925428
Anti-mouse KLRG1	2F1	PerCP-EF710	eBioscience	46-5893-82	AB_10670282
Anti-mouse KLRG1	2F1	PE	eBioscience	12-5893-82	AB_10596642
Anti-mouse KLRG1	2F1	APC	eBioscience	17-5893-82	AB_469469

Anti-mouse MHC Class II (I-A/I-E)	M5/114.15.2	eFluor 450	eBioscience	48-5321-82	AB_1272204
Anti-mouse NK1.1	PK136	eFluor 450	eBioscience	48-5941-82	AB_2043877
Anti-mouse NK1.1	PK136	PE-Cy7	eBioscience	25-5941-82	AB_469665
Anti-mouse NK1.1	PK136	Alexa Fluor 700	eBioscience	56-5941-82	AB_2574505
Anti-mouse NKp46	29A1.4	PerCP-EF710	eBioscience	46-3351-82	AB_1834441
Anti-mouse/human RORγt	AFKJS-9	APC	eBioscience	17-6988-82	AB_10609207
Anti-mouse/human RORγt	AFKJS-9	PE	eBioscience	12-6988-82	AB_1834470
Anti-mouse RORγt	B2D	PerCP-EF710	eBioscience	46-6981-82	AB_10717956
Anti-mouse TCRβ	H57-597		eBioscience	48-5961-82	AB_11039532
Anti-mouse TER-119	TER-119	eFluor 450	eBioscience	48-5921-82	AB_1518808
Anti-mouse TER-119	TER-119	PE-eFluor 610	eBiosciences	61-5921-82	AB_2574638
Anti-mouse TER-119	TER-119	PE-Cy7	eBioscience	25-5921-82	AB_469661
Anti-mouse/human Phospho-S6	cupk43k	PE-Cy7	eBioscience	25-9007-42	AB_2637099
Donkey anti-rabbit IgG (H+L)	N/A	Alexa Fluor 647	eBioscience	A-31573	AB_2536183
Anti-mouse CD4	RM4-5	BUV737	BD Biosciences	612843	AB_2870165
Anti-mouse CD25	PC61	PE-CF594	BD Biosciences	562694	AB_2744346
Anti-mouse CD45	30-F11	BUV395	BD Biosciences	564279	AB_2651134
Anti-mouse CD117 (cKit)	2B8	BV480	BD Biosciences	566081	AB_2739493
Anti-mouse NK1.1	PK136	BUV395	BD Biosciences	564144	AB_2738618
Anti-mouse Siglec-F	E50-2440	Alexa Fluor 647	BD Biosciences	562680	AB_2687570
Anti-mouse TCRβ	H57-597	BUV496	BD Biosciences	749915	AB_2874154
Anti-mouse CD90.2 (Thy1.2)	30-H12	BUV805	BD Biosciences	741909	AB_2871223
Anti-mouse T1/ST2	DJ8	FITC	MD Bioproducts	101001F	AB_947549
Anti-mouse Phosph-Gata3	3A1	N/A	Antibodies.com	ABIN555742	
Anti-mosue/human NFAT1	D43B1	PE	Cell Signaling Technology	14335S	AB_2798454

Other flow cytometry reagents

Reagent	Colour	Source	Catalog number	RRID
Streptavidin	BV421	BioLegend	405225	
Streptavidin	BV785	BioLegend	405249	

Zombie NIR	NIR	BioLegend	423106	
Streptavidin	BUV737	BD Biosciences	564293	AB_2869560
Fixable viability dye	EF780	eBioscience	65-0865-14	
MR1-tetramer	BV421	NIH Tetramer core facility		
2W1S-tetramer	PE	NIH Tetramer core facility		
CD1-tetramer	APC	NIH Tetramer core facility		

Data File S1. (separate file)

Excel spreadsheet of the CRISPR screen results performed to identify regulators of GATA3 and IL-13 in ILC2s.
